# Depletion and replacement of tissue-resident macrophages in mice with germ-line deletion of a conserved enhancer in the *Csf1r* locus

**DOI:** 10.1242/dev.205655

**Published:** 2026-07-15

**Authors:** Yajun Liu, Sebastien Jacquelin, Isis Taylor, Emma K. Green, Omkar L. Patkar, Sahar Keshvari, Ginell Ranpura, Conan J. O. O'Brien, Eline Jessen, Emma Maxwell, Rachel Allavena, Alexandre Gallerand, Stoyan Ivanov, Antony Adamson, Neil E. Humphreys, Kim M. Summers, Katharine M. Irvine, David A. Hume

**Affiliations:** ^1^ Mater Research Institute-University of Queensland, Translational Research Institute, Woolloongabba, Brisbane, QLD 4102, Australia; ^2^ School of Veterinary Science, The University of Queensland, Gatton, QLD 4343, Australia; ^3^ Université Côte d'Azur, CNRS, LP2M Nice, France; ^4^ Faculty of Biology, Medicine and Health, University of Manchester, Manchester M13 9PL, UK

**Keywords:** CSF1R, Monocyte, Macrophage, Enhancer, Transgenic mouse, Quantitative signalling

## Abstract

Expression of the *Csf1r* gene is regulated by a conserved enhancer, the fms-intronic regulatory element (FIRE). In mice with a germ-line deletion of FIRE (*Fireko*), CSF1R expression is undetectable in bone marrow progenitors and classical monocytes, but monocytopoiesis and non-classical monocyte maturation are unaffected. The loss of CSF1R is overcome in part by CSF2 *in vitro* and inflammatory recruitment *in vivo. Fireko* mice lack microglia and subpopulations of tissue-resident macrophages in peritoneum, kidney, heart, adipose, liver, skeletal muscle, pancreas, pituitary, adrenal and gonads. Heterozygous mutation impacts CSF1-induced proliferation and postnatal expansion of tissue macrophages. Physiological functions of the heart and kidney were not affected by the absence of macrophages. In a model of renal injury, macrophage recruitment and histopathology in wild-type and *Fireko* mice were indistinguishable, but there was a male-specific increase in serum creatinine and urea in the *Fireko* mice. Tissue-resident macrophages depleted in *Fireko* mice, including microglia, were replaced by donor-derived cells following intraperitoneal transfer of wild-type bone marrow at weaning. The *Fireko* mouse provides a platform to dissect functions of tissue-resident macrophages in development, homeostasis and pathology.

## Teaser

**Summary:** A unique model of selective tissue-resident macrophage deficiency arising from dysregulated expression of the mouse *Csf1r* gene.

## INTRODUCTION

The mononuclear phagocyte system (MPS) is a family of cells including progenitors in bone marrow, circulating blood monocytes and tissue-resident macrophages in every organ in the body (Grabert et al., 2020; Hume et al., 2019). Proliferation, differentiation and survival of cells of the MPS depends upon signals from the macrophage colony-stimulating factor receptor, CSF1R (Chitu and Stanley, 2017; Hume et al., 2020). *Csf1r^−/−^* mice (Dai et al., 2002), rats (Carter-Cusack et al., 2024; Keshvari et al., 2021; Pridans et al., 2018) and humans (Guo et al., 2019; Guo and Ikegawa, 2021; Oosterhof et al., 2019) lack bone-resorbing osteoclasts in bone, and have reduced density of tissue-resident macrophages in most organs. In the rodent models, *Csf1r^−/−^* pups are indistinguishable from littermates at birth, but postnatal skeletal development, somatic growth, organ maturation and fertility are severely compromised, leading to early mortality (Carter-Cusack et al., 2024; Chitu and Stanley, 2017; Keshvari et al., 2021). On a pure C57BL/6J mouse background, homozygous *Csf1r* null mutation leads to perinatal lethality (Percin et al., 2018) whilst on the FVB/J background, few pups survive to weaning due to hydrocephalus (HC) (Erblich et al., 2011).

We recently described a hypomorphic mutation in the mouse *Csf1r* gene (Rojo et al., 2019). These mice harbour a deletion of a 300 bp enhancer region (the Fms intronic regulatory element; FIRE) that is conserved from reptiles and birds to humans (Hume et al., 2017). Homozygous mutant mice lack macrophages in the embryonic yolk sac and fetal liver (Munro et al., 2020) but, unlike *Csf1r*^−/−^ mice (Dai et al., 2002), *Csf1r*^ΔFIRE/ΔFIRE^ (hereafter *Fireko*) mice are not osteoclast deficient and/or osteopetrotic or growth-inhibited, and were viable and fertile as adults (Rojo et al., 2019). The analysis of *Fireko* mice has focused mainly on the impacts of microglial deficiency (reviewed by Hume, 2025). *Fireko* mice also lack resident macrophages in the peritoneal cavity, and omentum, epidermis, kidney, heart and salivary gland (Louwe et al., 2022; McKendrick et al., 2023; Rojo et al., 2019), whereas in other organs (liver, spleen, intestine and lung), CSF1R-dependent resident macrophages appeared unaffected and *Csf1r* mRNA was expressed normally. The molecular basis for the selective impact of the *Fireko* mutation is unknown.

Like homozygous *Csf1r* or *Csf1* mutation (Chitu and Stanley, 2017; Percin et al., 2018), the *Fireko* mutation was lethal on the C57BL/6J background (Taylor et al., 2025 preprint). The *Csf1r*^ΔFIRE^ allele was intercrossed with a *Csf1r-*EGFP transgenic line (Sasmono et al., 2003) also backcrossed to C57BL/6J. On this congenic background, *Fireko* mice were identified at 59% of expected Mendelian frequency at weaning. Survival was attributed to retention of non-C57BL/6J genomic sequences, despite the backcross (Taylor et al., 2025 preprint). Of the homozygous pups identified, around one-third developed severe HC. The remainder were long-lived, healthy and fertile.

In the present study, we take advantage of the congenic line to extend the investigation of the *Fireko* mouse to a more-comprehensive range of peripheral tissues. We explore the mechanisms that underlie selective loss of macrophages in some tissues but not in others and the impact of heterozygous mutation on CSF1 responsiveness. We demonstrate that intraperitoneal (IP) injection of wild-type marrow in *Fireko* mice at weaning leads to selective population of the vacant macrophage territories with donor-derived cells whilst making no contribution to blood monocytes.

## RESULTS

### The loss of CSF1R in progenitors does not alter myeloid lineage commitment or monocytopoiesis

**Fig. 1.**
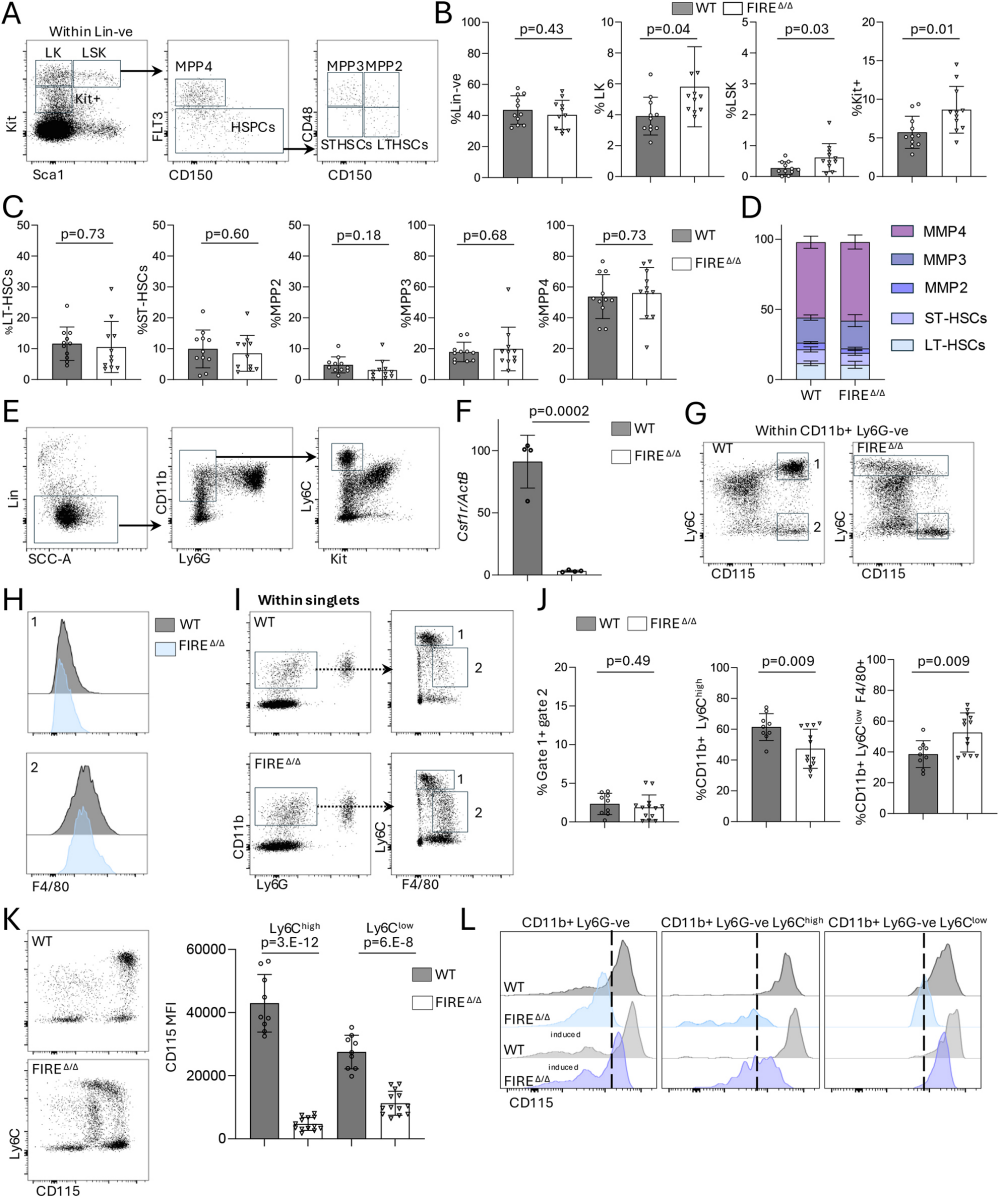
**CSF1R expression and monocytopoiesis in *Fireko* mice.** Bone marrow (BM) and blood cells from wild-type and *Fireko* (FIRE^Δ/Δ^) mice on the inbred C57BL/6J background were isolated and analysed by flow cytometry, as described in the Materials and Methods. (A) BM progenitors were defined as lineage-negative (Lin−) and further divided into multipotent progenitors (MPPs), long-term and short-term hematopoietic stem cells (LT-HSCs and ST-HSCs), based upon detection of KIT, SCA1, FLT3, CD150 and CD48, as shown in the gating strategy in Fig. S1. (B) Proportions of Lin− cells (% total BM cells) and of subpopulations (% of Lin− cells) in wild-type and FIRE^Δ/Δ^ BM. (C,D) Proportions of LT-HSCs, ST-HSCs, MPP2, MPP3 and MPP4 expressed as a percentage of the Lin^−^, Sca1^+^, Kit^+^ population. (E) BM Ly6C^high^ monocytes were defined as CD11b^+^, Ly6C^+^, Ly6G^−^, Kit^−^, as indicated in the gating strategy shown. (F) Ly6C^high^ monocytes were isolated from wild-type and FIRE^Δ/Δ^ BM, as described in the Materials and Methods, and *Csf1r* mRNA levels were determined by qRT-PCR (*n*=4). (G) Representative flow cytometry profiles of CD115 detection in CD11b^+^, Ly6G^−^ BM cells from wild-type and FIRE^Δ/Δ^ mice. (H) Representative profiles of expression of F4/80 in Ly6C^high^ (population 1) and Ly6C^−^ (population 2) subpopulations of CD11b^high^/Ly6G^−^ BM cells outlined in G. (I) Peripheral blood monocytes were defined as Ly6G^−^, CD11b^high^, F4/80^+^, as indicated in the gating strategy shown. (J) Left panel shows quantitation of monocytes (combined areas 1 and 2 in I) as a percentage of total cells in wild type and FIRE^Δ/Δ^. Middle and right panels show proportions of Ly6C^high^ and Ly6C^low^ cells within the combined monocyte gate (I). (K) Representative flow cytometry profiles and quantitation of mean fluorescence intensity (MFI) of CD115 detection in CD11b^+^, Ly6G^−^ cells in blood from wild-type and FIRE^Δ/Δ^ mice. (L) Representative profiles of CD115 detection in monocyte fractions defined as in I. Cells were analysed with (lower panels) or without (upper panels) preincubation for 90 min at 37°C. Data are mean±s.d. *P*-values were determined using an unpaired two-tailed *t*-test. Each point in the graphs is a result from an individual animal.

Given the evidence of an interaction between *Csf1r* mutations and genetic background, we first revisited expression of CSF1R and surface markers of progenitor populations and blood leukocytes on the inbred C57BL/6J congenic background. *Fireko* mice are not osteopetrotic, and the bone marrow cavity and growth plate appear histologically normal. On the original background, the total bone marrow total cellularity obtained by flushing the femur was unchanged compared to wild type (Rojo et al., 2019). We confirmed that this was the case on the C57BL/6 background (Fig. S1B). We therefore analysed bone marrow cellular composition as a percentage of total cells. During lineage commitment, CSF1R is first expressed on the MPP3 subset of progenitors (Maxwell et al., 2026). We found no significant differences between wild type and *Fireko* in the relative abundance of hematopoietic stem cells (HSCs) or multipotent progenitor (MPP) subsets defined by surface markers, including the growth factor receptors KIT and FLT3 (Fig. 1A-D, see gating strategy in Fig. S1A). As reported on the original background (Rojo et al., 2019), CD115 and *Csf1r* mRNA were almost undetectable in *Fireko* marrow monocytes (Fig. 1E,F). Mouse blood monocytes have been divided into classical and non-classical subpopulations based upon cell-surface markers, including Ly6C (Geissmann et al., 2010). The percentage of CD11b^high^/Ly6G^−^ cells in the bone marrow did not distinguish *Fireko* from wild type. Within that population, subpopulations were defined as Ly6C^high^ and Ly6C^low^/F4/80^+^, as previously described (Rojo et al., 2019). There was no significant difference in the relative proportion of Ly6C^low^/F4/80^+^ cells in the *Fireko* mouse compared to wild type (Fig. S1C). F4/80^high^ bone marrow resident macrophages associated with hematopoietic and/or erythroblastic islands are fragmented during tissue disaggregation and mainly appear as remnants associated with unrelated cells on flow cytometry (Millard et al., 2021). In wild-type marrow, we detected two populations of Ly6G^−^/CD11b^high^/CD115^+^ cells. Within the Ly6C^high^ cells (population 1), which we identify as classical monocytes, CD115 expression was selectively lost in the *Fireko* mouse (Fig. 1G). The Ly6C^high^ bone marrow cells expressed low levels of F4/80 (Fig. 1H). By contrast, the Ly6G^−^/CD11b^high^/Ly6C^−^ cells (population 2 in Fig. 1G) retained CD115 expression in the *Fireko* mice. These cells expressed high levels of F4/80 in both wild-type and *Fireko* mice (Fig. 1H), consistent with their identity as marrow-resident macrophages (Hume et al., 1983).

Mouse spleen can also provide a reservoir of blood monocytes that are mobilized in response to inflammation (Swirski et al., 2009). Conditional deletion of *Csf1* in mice did not alter the total number of splenic monocytes but led to an increase in the Ly6C^high^/Ly6C^low^ ratio (Emoto et al., 2022). The spleen to body weight ratio, cellular architecture and relative area of F4/80^+^ red pulp was not altered in the *Fireko* mice (Rojo et al., 2019). The *Fireko* mutation did not alter the percentage of CD11b^high^/Ly6G^−^ cells in spleen compared to wild type (Fig. S1D). As in marrow, the identification of Ly6C^low^ monocytes in spleen is confounded by fragmentation of the abundant F4/80^high^ red pulp macrophage population (Millard et al., 2021). Within the CD11b^high^/Ly6G^−^ population, cells defined as Ly6C^low^/F4/80^low^ (Swirski et al., 2009) were marginally increased relative to Ly6C^high^ in the *Fireko* mice (Fig. S1D).

On the original background, the *Fireko* mutation had no significant effect on total white cell, red cell or platelet count expressed/ml blood or on relative neutrophil, lymphocyte or monocyte counts defined by an automatic analyser (Rojo et al., 2019). This was also the case on the C57BL/6 background. As in marrow and spleen, the absolute monocyte count in peripheral blood was unchanged. Within the CD11b^high^/Ly6G^−^ population, we used F4/80 as a positive marker (MacDonald et al., 2010) to distinguish monocytes from CD11b^+^ NK cells (Chiossone et al., 2009). The relative proportion of F4/80^+^Ly6C^low^ versus Ly6C^high^ blood monocytes defined on this basis was significantly increased in the *Fireko* mice (Fig. 1I,J). Fig. 1K shows the profile of CD115 detection in the CD11b^+^/Ly6G^−^ population from wild-type and *Fireko* mice. In *Fireko* mice, we observed a complex pattern of down-regulation of CD115 compared to wild type, with selective depletion in Ly6C^high^ cells and the appearance of a Ly6C^int^/CD115^low^ population (Fig. 1K). In the *Fireko* mice, Ly6C^low/−^ cells retained significant CD115 expression. Based on evidence that Ly6C^high^ monocytes regulate CSF1 availability in blood (Yona et al., 2013), we reasoned that surface receptors might be masked by endogenous CSF1 in the *Fireko* mouse*.* To test this possibility, leukocytes were washed and incubated at 4°C for 90 min *in vitro* to allow ligand dissociation prior to staining. As shown in Fig. 1L, pre-incubation revealed increased detection of surface CD115 in CD11b^+^/Ly6G^−^ cells in both wild-type and *Fireko* mice. In the *Fireko* mice*,* the increase occurred predominantly in the Ly6C^low^ cells, but expression was still 5- to 10-fold lower than in wild type.

In overview, the analysis of the impact of the *Fireko* mutation on monocytes and progenitors on the C57BL/6J background revealed no substantial differences compared to the mixed background (Rojo et al., 2019). Importantly, the results indicate that *Csf1r* expression in Ly6C^high^ monocytes is not required for the transition to the Ly6C^low^ state. Furthermore, the mutation does not entirely prevent CSF1R expression in Ly6C^low^ monocytes or tissue-resident macrophages in bone marrow.

### *Fireko* mice are unresponsive to CSF1

Bone marrow cells from *Fireko* mice do not respond to CSF1 *in vitro* (Rojo et al., 2019). To confirm the CSF1 unresponsiveness of *Fireko* mice, we injected a human CSF1-mouse IgG2A Fc fusion protein. Recombinant human CSF1 injection in mice causes monocytosis and expansion of tissue macrophages (Hume et al., 1988); the fusion to Fc increases efficacy by extending the otherwise short circulating half-life. Administration of the mouse-human CSF1-Fc or a pig CSF1-Fc protein causes expansion of monocyte numbers in marrow, monocytosis, an increase in the number of tissue macrophages, and growth of the liver and spleen in wild-type mice (Gow et al., 2014; Keshvari et al., 2024). Cohorts of wild-type, *Csf1r*^ΔFIRE/WT^ and *Fireko* mice were treated with CSF1-Fc on 4 successive days and analysed on day 5. Prior to CSF1 administration, we assessed the body weight, fat mass and lean mass by NMR (Minispec) and found no significant difference between groups (Fig. S2A), despite the reported role of CSF1R-dependent macrophages in adipose tissue development (Cox et al., 2021). CSF1-induced increases in liver weight and bone marrow, and blood Ly6C^high^ monocyte numbers reported previously (Gow et al., 2014; Keshvari et al., 2024) were detected in both wild-type and heterozygous mice, and were entirely absent in *Fireko* mice (Fig. S2B-D). Responses to CSF1-Fc that were not affected by the *Csf1r* mutation include thrombocytopenia and splenomegaly (Fig. S2E,F), likely reflecting the fact that CSF1R expression in the spleen is not affected by the mutation (Rojo et al., 2019).

### CSF1R expression in *Fireko* mice is induced by CSF2

**Fig. 2.**
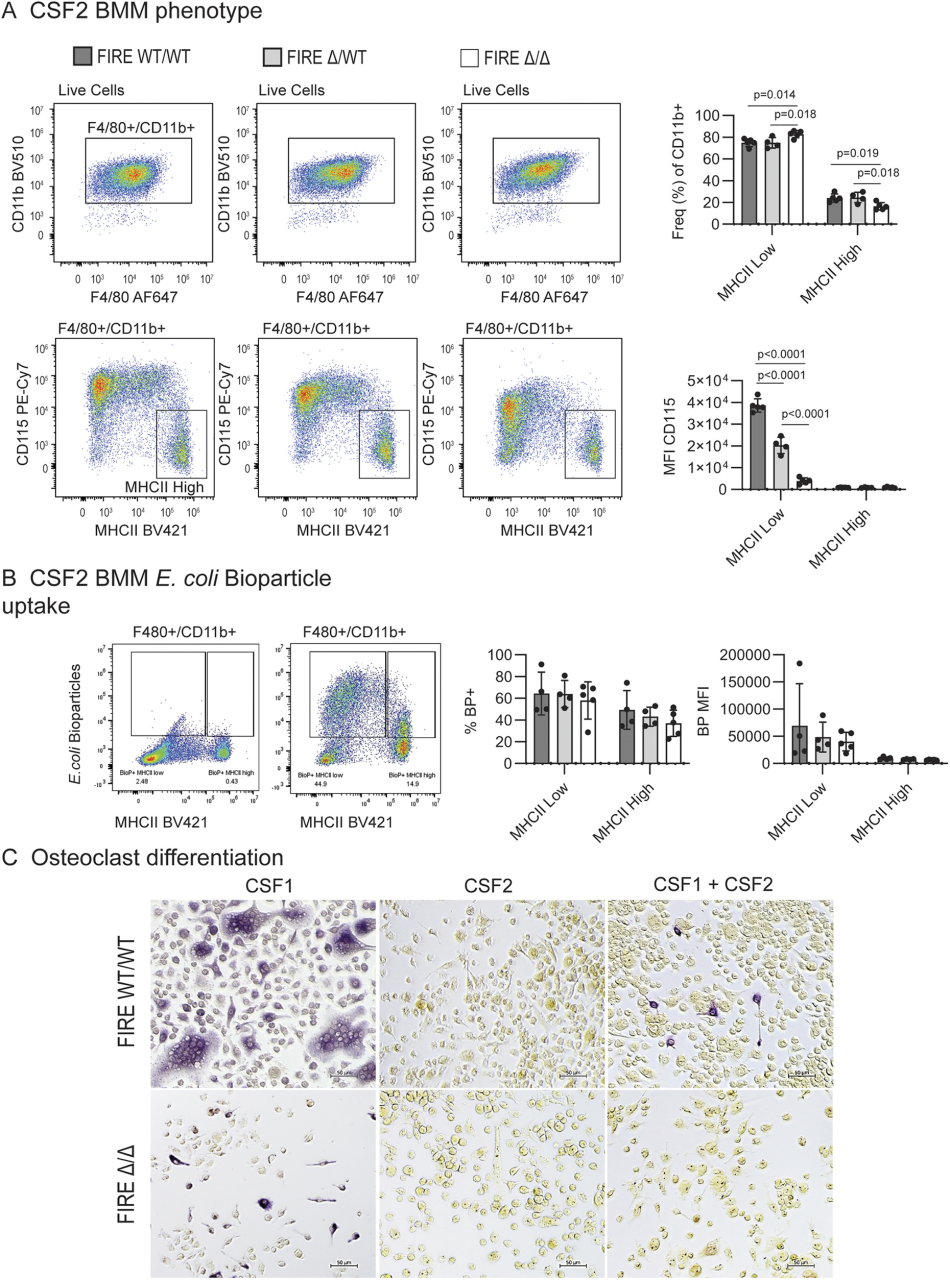
**Regulation of CSF1R expression and differentiation in *Fireko* mice by CSF2.** (A) Bone marrow-derived macrophages (BMMs) from wild-type and *Fireko* mice were generated by culture in CSF2 for 7 days followed by staining and flow cytometry analysis. F4/80/CD11b^+^ cells were gated based on MHCII expression. The relative proportions of MHCII high and low subsets, and their CSF1R mean fluorescence intensity (MFI) are shown. Data are mean±s.d. Data were analysed using two-way ANOVA with Šidák's multiple comparison test. (B) CSF2-derived BMMs were incubated with pH Rodo *E. coli* bioparticles (BPs) for 1 h followed by surface staining and analysis by flow cytometry for BP uptake (%) and MFI in cell subsets. Data are mean±s.d. (C) Representative images of bone marrow from wild-type or *Fireko* mice cultured in CSF1 or CSF2, or both, for 3 days prior to the addition of RANKL and continued culture until day 7. Cells were stained for tartrate-resistant acid phosphatase (TRAP, purple).

Consistent with absence of detectable CSF1R, bone marrow cells from *Fireko* mice did not respond to CSF1 in liquid culture, as shown previously (Rojo et al., 2019). By contrast, cells from wild-type, *Csf1r*^ΔFIRE/WT^ and *Fireko* mice responded equally to granulocyte-macrophage colony-stimulating factor (GM-CSF, CSF2) to generate a spectrum of myeloid cells. In wild-type cultures, two major subpopulations were identified: F4/80^high^/CSF1R^high^/MHCII^low^ and F4/80^low^/CSF1R^low^/MHCII^high^ (Fig. 2A). The latter population includes a mixture of classical dendritic cells and macrophage-like antigen-presenting cells (Helft et al., 2015). Both populations were able to internalise pH Rodo-labelled *E. coli* bioparticles but the uptake measured as median fluorescence intensity was greater in MHCII^low^ cells (Fig. 2B). Within the MHCII^low^ macrophage population, CSF1R expression in *Fireko* was detected at 10-15% of the wild-type level. Expression in *Csf1r*^ΔFIRE/+^ macrophages was intermediate between the two (Fig. 2A). These observations confirm that CSF2 can promote the expression of *Csf1r* in *Fireko* bone marrow progenitors but does not entirely overcome the effect of the enhancer deletion.

Mice with mutations in *Csf1* or *Csf1r* lack osteoclasts (OCLs) and are osteopetrotic (Dai et al., 2002), whereas *Fireko* mice have normal bone development. We next asked whether bone marrow cells from *Fireko* mice could generate OCL in response to stimulation with RANK ligand (Ovchinnikov et al., 2010). Bone marrow cells were cultured in CSF1 or CSF2, or a combination, for 3 days prior to addition of RANKL, and culture was continued until day 7. In wild-type bone marrow, addition of RANKL to cultures expanded in CSF1 led to the generation of multinucleated OCL-expressing tartrate-resistant acid phosphatase (TRAP). This response was absent in cells grown in CSF2 and prevented in the continued presence of CSF2 (Fig. 2C). Hence CSF2 is unlikely to be responsible for the generation of OCL in the *Fireko* mice*.*

### *Fireko* mice respond to inflammation in the peritoneal cavity

**Fig. 3.**
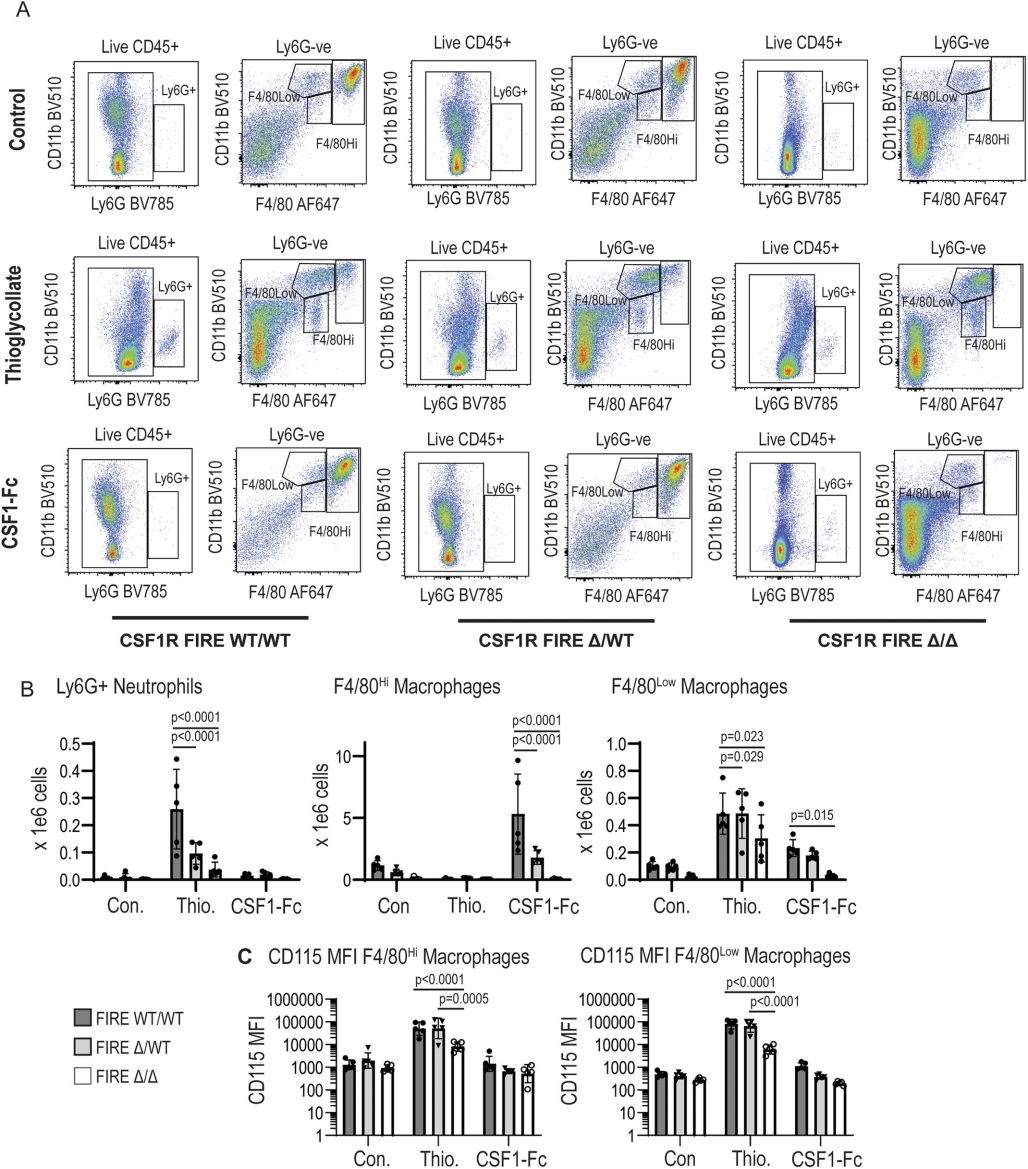
**Analysis of the effect of homozygous and heterozygous ΔFIRE mutation on resident and elicited peritoneal macrophages.** Wild-type, *Csf1r*^ΔFIRE/+^ and *Fireko* mice were administered 1 ml thioglycolate intraperitoneally, or given four subcutaneous injections of 1 mg/kg CSF1-Fc or saline control. Peritoneal lavage was collected 5 days post thioglycolate or CSF1-Fc administration, and analysed by flow cytometry. (A) Representative flow cytometry profiles of Ly6G^−^ populations defined by expression of CD11b and F4/80 following different treatment in each genotype. (B) Quantification of Ly6G^+^ neutrophils, and F4/80^High^ and F4/80^Low^ macrophages in control and post thioglycolate lavage. Each point is a result from an individual animal. (C) Quantification of CD115 mean fluorescence intensity (MFI) on F4/80^High^ and F4/80^Low^ macrophages in control and post thioglycolate lavage. Separate panels in B and C show quantification of Ly6G^+^ neutrophils, and F4/80^High^ and F4/80^Low^ macrophages in peritoneal lavage from control and CSF1-Fc-treated mice. Each point is a result from an individual animal of the indicated genotype. Data are mean±s.d. A two-way ANOVA with Šidák's multiple comparison test was used to test for significance.

The effect of CSF1/CSF1R inhibition on the generation of an inflammatory exudate in the peritoneal cavity has varied in previous studies that used different antibodies and treatment regimens (Louis et al., 2015; MacDonald et al., 2010). However, thioglycolate-elicited peritoneal macrophages (TEPMs) are autocrine for CSF1R signalling (Irvine et al., 2006) and ChIP-seq analysis of TEPM revealed multiple active regulatory elements within the *Csf1r* locus, in addition to FIRE (Fonseca et al., 2019). We therefore asked whether resident peritoneal macrophages, which are absent in *Fireko* mice, are required to initiate the response, and whether inflammatory recruitment could enable expression of CSF1R. Thioglycolate injection led to the loss of large F4/80^High^ peritoneal macrophages, i.e. the macrophage disappearance reaction (Barth et al., 1995), in wild-type mice, as expected, and accumulation of F4/80^Low^ macrophages (derived from recruited monocytes) regardless of genotype (Fig. 3A,B). Surface CD115 was detected on *Fireko* TEPMs but at significantly lower levels than in wild-type and heterozygous mice (Fig. 3C). Interestingly, granulocyte recruitment was reduced in the *Fireko* mice (Fig. 3B), suggesting a role for resident macrophages in acute inflammation.

We also analysed the peritoneal macrophage populations in mice treated with CSF1-Fc (Fig. 3B,C). CSF1-Fc treatment in wild-type mice caused an increase in total yield of both F4/80^high^ and F4/80^low^ peritoneal macrophages. The expansion of both peritoneal macrophage populations in response to CSF1-Fc was significantly attenuated in the heterozygous mutant mice. Consistent with the lack of effect on blood monocytes, CSF1-Fc treatment did not expand the F4/80^low^ peritoneal population and it did not overcome the complete absence of F4/80^high^ peritoneal macrophages in the *Fireko* mice*.*

### The conserved AP1 element in FIRE contributes to the rate of *Csf1r* transcription but is not essential for maintenance of tissue macrophages

**Fig. 4.**
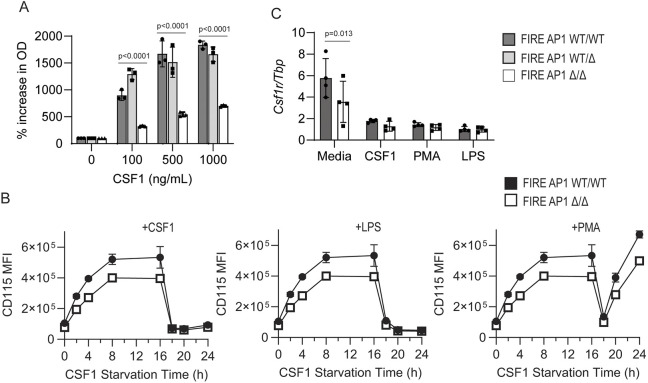
**The *Csf1r*^ΔAP1-FIRE/ΔAP1-FIRE^ mutation regulates CSF1R expression.** (A) Bone marrow cells from *Csf1r*^+/+^ (FIRE AP1 WT/WT), *Csf1r*^+/ΔAP1^ (FIRE AP1 WT/Δ) or *Csf1r*^ΔAP1/ΔAP1^ (FIRE AP1 Δ/Δ) mice were cultured for 7 days in recombinant human CSF1 at the indicated concentrations and metabolic activity was measured by resazurin assay. (B) CSF1-differentiated bone marrow macrophages were starved of CSF1 for 2, 4, 8, 16 and 24 h, or starved of CSF1 for 16 h followed by stimulation with CSF1, LPS or PMA for 2, 4 and 8 h, and surface expression of CSF1R (CD115) was analysed by flow cytometry. MFI, median fluorescence intensity. (C) CSF1-differentiated bone marrow macrophages were starved of CSF1 for 16 h followed by stimulation with medium control, CSF1, PMA or LPS for 8 h. *Csf1r* mRNA expression was analysed by qPCR. Data are mean±s.d. A two-way ANOVA with Šidák's multiple comparisons test was used to test for significance.

The FIRE sequence includes a core AP1-ETS element conserved from reptiles to mammals (Hume et al., 2017). ChIP-seq analysis revealed binding of multiple AP1 family members to FIRE (Fonseca et al., 2019). The AP1 sequence contains the transcription start site of an anti-sense enhancer RNA that is induced by stimuli that repress *Csf1r* transcription (Sauter et al., 2013). To test the function of this element, we mutated the AP1 site in the mouse germ line to a sequence that does not bind AP1, as described in detail in the Materials and Methods. The core motif (TGAATCA) in FIRE was substituted with TCTAGAG, which also created an Xba1 site to enable genotyping. The mutation was confirmed by sequencing and bred to homozygosity. Comprehensive analysis of tissue macrophage populations in *Csf1r*^ΔAP1-FIRE/ΔAP1-FIRE^ mice revealed no loss of any of the populations missing in *Fireko* mice, including microglia (Fig. S3A). Peritoneal macrophage populations and their expression of surface CSF1R were unchanged compared to wild type (Fig. S3B). We next asked whether mutation impacted CSF1 responses *in vitro. Csf1r*^ΔAP1-FIRE/ΔAP1-FIRE^ bone marrow cells were significantly less responsive to CSF1 (Fig. 4A). Bone marrow-derived macrophages (BMDM) were cultured in CSF1 to maximally downregulate surface protein and *Csf1r* mRNA, then washed to remove the ligand; reappearance of surface CSF1R (CD115 antigen) was measured with time. The *Csf1r*^ΔAP1-FIRE/ΔAP1-FIRE^ mutation reduced the rate of resynthesis of the receptor following CSF1 removal and compromised expression of *Csf1r* mRNA (Fig. 4B). Surface CSF1R on BMDM is acutely downregulated via ectodomain cleavage following protein kinase C (PKC) activation using phorbol myristate acetate (PMA) and by TLR4 activation following addition of lipopolysaccharide (LPS) (Sester et al., 1999). In the case of PMA, the effect is transient, due to degradation of PKC, and CSF1R reappears. The AP1 mutation also decreased the rate of reappearance of CSF1R in PMA-treated cells (Fig. 4B). However, the mutation did not allow CSF1R protein, or *Csf1r* mRNA, to be re-expressed in the presence of LPS.

### *Fireko* mice lack defined subpopulations of tissue-resident macrophages: evidence for haploinsufficiency in heterozygous mutants

**Fig. 5.**
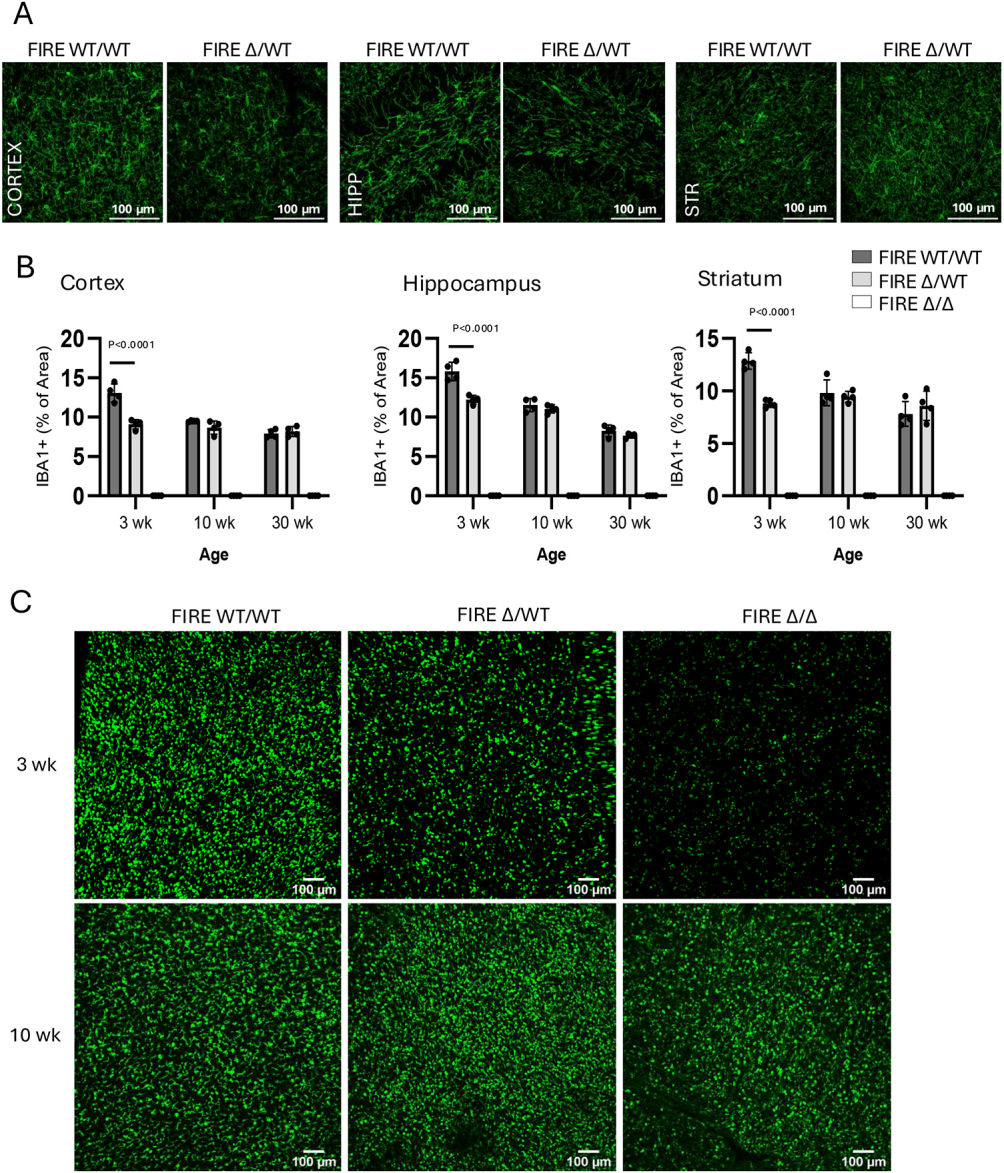
**Depletion of microglia and lung macrophages in *Csf1r*^ΔFIRE/+^ mice.** Brain sections from wild-type, *Csf1r*^ΔFIRE/+^ and *Fireko* mice at 3, 10 or 30 weeks of age were stained for IBA1 in the cortex, hippocampus and striatum. (A) Representative images from 3-week-old mice. (B) Quantification of the IBA1^+^ stained area by image analysis. Each point is a result from an individual animal. Data are mean±s.d. Data were analysed using a two-way ANOVA with Tukey's multiple comparisons test. (C) Representative whole-mount images of *Csf1r-*EGFP in wild-type, *Csf1r*^ΔFIRE/+^ and *Fireko* mice at 3 and 10 weeks.

*Csf1r* mRNA and surface CD115 are each reduced by 50% in monocytes and macrophages in heterozygous *Csf1r*^ΔFIRE/+^ mice compared to wild type (Rojo et al., 2019). Tissue-resident macrophages, including microglia, undergo a CSF1-dependent expansion in the first weeks of postnatal life (Summers and Hume, 2017) that is delayed in mice with a heterozygous kinase-dead *Csf1r* mutation (Stables et al., 2022). In view of this evidence and the apparent haploinsufficiency, we speculated that *Csf1r*^ΔFIRE/WT^ mice might exhibit a similar delay. We found that microglial density was significantly reduced in *Csf1r*^ΔFIRE/WT^ mice compared to wild type at 3 weeks of age (Fig. 5A,B). In contrast to the microgliosis reported in *Csf1r^+/−^* mice on the C57BL/6J background (Chitu et al., 2015), there was no significant difference between wild-type and *Csf1r*^ΔFIRE/WT^ mice at 10 or 30 weeks (Fig. 5A,B).

Subsequent to the original description of the *Fireko* mice (Rojo et al., 2019), others described partial macrophage deficiency in salivary gland (McKendrick et al., 2023) and omentum (Louwe et al., 2022). To fully evaluate the extent of macrophage deficiency in these mice, we used whole-mount imaging of the *Csf1r*-EGFP reporter (Sasmono et al., 2003). *Csf1*^op/op^ mice have a deficiency in alveolar macrophages at 3 weeks that resolves with age (Shibata et al., 2001). The abundance of SiglecF-positive alveolar macrophages was previously found to be unaffected in adult *Fireko* mice (Rojo et al., 2019). A separate population of interstitial macrophages is poorly recovered following tissue disaggregation (Hume et al., 2023). In adult mice, *Csf1r-*EGFP is uniformly expressed by isolated alveolar macrophages (Sasmono et al., 2003). In wild-type embryonic lung, *Csf1r-*EGFP is expressed by two morphologically distinct populations of interstitial macrophages, distinguished by mutually exclusive expression of MAC2 (*Lgals3*) and F4/80, respectively. *Csf1r-*EGFP^+^ interstitial (F4/80^+^) and alveolar (MAC2^+^) macrophages expand during the first 3 weeks of postnatal life (Jones et al., 2013; Tan and Krasnow, 2016). At 3 weeks of age, *Fireko* mice were deficient in *Csf1r-*EGFP^+^ cells in the lung and heterozygous mutant mice appeared intermediate in phenotype (Fig. 5C). By 10 weeks of age there was no longer a clear distinction between genotypes, consistent with the previous analysis of adult *Fireko* mice (Rojo et al., 2019).

The testis contains two distinct macrophage populations with distinct ontogeny: interstitial macrophages that interact with Leydig cells and regulate steroidogenesis; and peritubular macrophages that interact with Sertoli cells (Gu et al., 2023). Both are readily imaged in adult testis using the *Csf1r-*EGFP reporter, and the stellate peritubular population is selectively depleted in *Fireko* mice (Fig. S4A). The loss of peritubular cells was confirmed by localisation of F4/80 (Fig. S4A). Langerhans cells (LCs), the macrophages of the epidermis, are also derived from embryonic progenitors and expand rapidly in the postnatal period (Mass et al., 2023). We confirmed that LCs were depleted in the ear of adult mice, as reported previously (Fig. S4B) (Rojo et al., 2019). At 3 weeks of age, *Fireko* mice were devoid of detectable LCs in the more-stratified epidermis of the footpad (data not shown) but this deficiency was fully resolved by 10 weeks (Fig. S4B).

The macrophages of the pancreas have not yet been examined in detail in the *Fireko* mice. Three distinct populations of macrophages have been identified in *Fireko* mice: two interstitial macrophages within the exocrine pancreas that differ in self-renewal capacity; and a population within the islets (Calderon et al., 2015). Based upon detection of *Csf1r-EGFP* in whole mounts, interstitial macrophages were reduced in number in *Fireko* mice at 10 weeks but partly replenished at 30 weeks (Fig. S4C). *Fireko* mice lack cardiac muscle macrophages on the inbred background (see below). The abundance of *Csf1r-EGFP* macrophages was also greatly reduced in skeletal muscle in the diaphragm, which can be readily imaged in wholemounts (Fig. S4D). In adipose tissue at 10 weeks, we noted an apparent reduction in stellate interstitial *Csf1r-*EGFP-positive macrophages in epididymal fat pads (Fig. S4E).

The liver contains a subcapsular network of CSF1-dependent macrophages that can be visualized with the *Csf1r* reporter transgene (Sierro et al., 2017). These cells were not detected in the *Fireko* liver, whereas the underlying F4/80^+^ Kupffer cells were retained (Fig. S5A). By contrast, the CD169^+^ marginal zone metallophils of the spleen, which are also CSF1/CSF1R dependent (Stables et al., 2024; Witmer-Pack et al., 1993) were unaffected in *Fireko* mice (Fig. S5B).

**Fig. 6.**
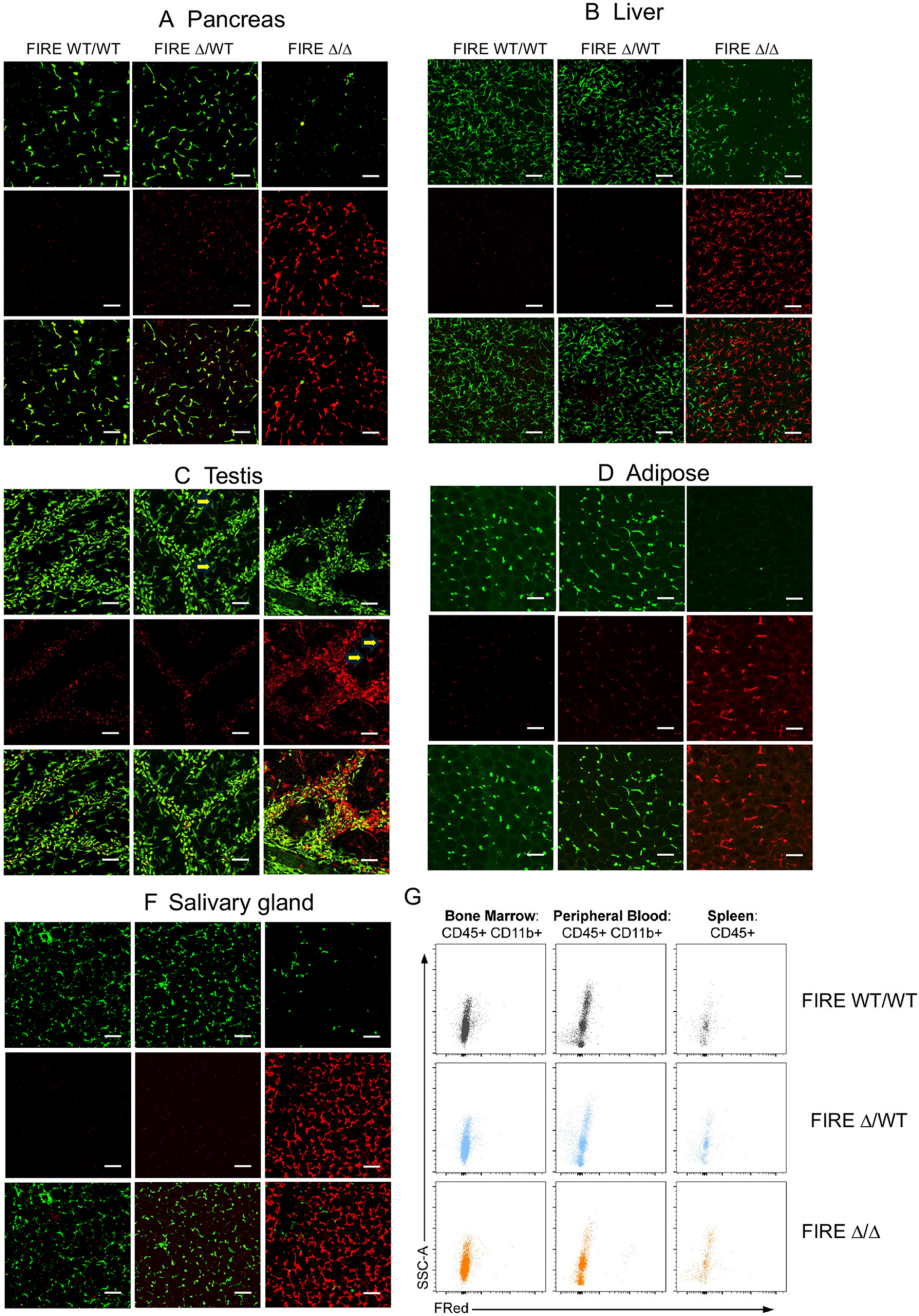
**Donor cell population of tissue macrophages in *Fireko* mice following transfer of wild-type bone marrow.** *Csf1r-*EGFP^+^ wild-type, *Csf1r*^ΔFIRE/+^ and *Fireko* mice were injected intraperitoneally at weaning with wild-type *Csf1r-*FRed donor cells, and chimerism was assessed by whole-mount imaging at 12 weeks post transfer. (A-F) Images of selected tissues are representative of at least five bone marrow transfer recipients analysed. Arrows in C indicate peritubular macrophages. The three rows in each panel show EGFP (top), Fred (middle) and merged (bottom) fluorescence detection on the same section. Scale bars: 50 μm. (G) Typical flow cytometry profiles of bone marrow, blood and spleen cells, demonstrating the absence of detectable FRed^+^ donor cells following bone marrow transfer.

Fig. S6 contains additional representative immunohistochemistry images and quantitation of tissue-resident macrophages in adult mice from the congenic C57BL/6J *Fireko* line. F4/80^+^ macrophages were present in white and brown adipose tissue in both male and female wild-type and *Fireko* mice (Fig. 6A). In contrast to reported depletion of F4/80^+^ macrophages in salivary glands on the original background (McKendrick et al., 2023) these cells were partly retained (Fig. S6B). Interstitial macrophages in the pancreas were not readily detected with F4/80, so IBA1 was used as a marker. This revealed a selective reduction in interstitial macrophages in the *Fireko* mice, consistent with EGFP detection, whereas a distinct population on the surface of lobules was increased (Fig. S6C). F4/80 was detected on macrophages within islets of Langerhans, and this population was absent in *Fireko* mice (Fig. S6D). In other endocrine tissues, the abundant F4/80^+^ macrophage population of the adrenal cortex and the microglia-like population of the medulla (Hume et al., 1984) were both absent in the *Fireko* mouse (Fig. S6E). In the *Fireko* ovary, interstitial F4/80^+^ macrophages were reduced, and the recruited macrophages that populate the wild-type corpus luteum (Hume et al., 1984) were absent (Fig. S6F). Finally, in the pituitary gland, the microglial-like cells of the posterior lobe were absent in the *Fireko* mouse and those of the anterior lobe were reduced in number, but a border-associated macrophage population was unaffected (Fig. S6G).

### Renal macrophage deficiency in *Fireko* mice does not impact basal kidney function or the development of chronic kidney disease

To analyse the impact of congenital macrophage deficiency on organ function, we focused on the kidney. In terms of gene expression profile, resident kidney macrophages share some features with microglia (Summers et al., 2020). Adult *Fireko* mice have previously been shown to lack F4/80^high^ kidney macrophages using flow cytometry analysis (Rojo et al., 2019). IBA1^+^ immunostaining highlighted the reduction in macrophages in cortex and medulla in both *Fireko* and heterozygous mutant mice (*Csf1r*^ΔFIRE/WT^) compared to wild type (Fig. S7A,B). In contrast to other organs, the deficiency in heterozygous mice did not resolve with age. Localization of F4/80 confirmed the complete absence of the abundant F4/80^high^ renal macrophage populations (Hume and Gordon, 1983) in *Fireko* mice (Fig. S7C). Residual IBA1^+^ cells in the *Fireko* kidney medulla are likely the retained F4/80^low^ monocytes and monocyte-derived cells detected by flow cytometry (Rojo et al., 2019). *Csf1r* knockout in rats is associated with impaired postnatal kidney development, including severe renal medullary hypoplasia (Keshvari et al., 2021). Fig. S7A,B includes localization of the vascular marker CD31 in 3- and 10-week-old wild-type and *Fireko* mice, highlighting glomeruli in the cortex and major vessels in the medulla. No gross anatomical differences were evident in the mutant mice. Whereas *Adgre1* mRNA was depleted in *Fireko* mice, consistent with loss of detectable F4/80, the apparently normal histology and function of *Fireko* kidneys (see below) was supported by unchanged detection of mRNA encoding markers of podocytes (*Nphs2*), proximal tubules (*Slc22a13*), distal tubules (*Umod*) or collecting ducts (*Aqp2* and *Scnn1b*) in total kidney mRNA (Fig. S7D). Conventional veterinary pathology analysis of serum electrolytes, proteins and renal functional markers (creatinine and urea) in serum revealed no significant difference between wild-type, *Csf1r*^ΔFIRE/WT^ and *Fireko* mice at 10 or 30 weeks of age (Fig. S7E).

Renal medullary macrophages were recently shown to have a role in monitoring urine contents to prevent accumulation of sedimentary particles (He et al., 2024). Macrophage depletion led to a large increase in kidney stone formation in a hyperoxaluria challenge model. Similarly, depletion of renal resident macrophages was reported to exacerbate pathology in acute tubular injury following cisplatin exposure (Salei et al., 2021). To analyse the function of resident kidney macrophages and CSF1R signalling in response to epithelial injury, we exposed mice to dietary adenine. Adenine metabolism in rodents leads to the production of 2,8-dihydroxyadenine crystals, which accumulate in the renal tubules, causing mechanical obstruction, tubular injury and inflammation, which is typically more severe in male mice (Diwan et al., 2018; Makhloufi et al., 2020; Miguel et al., 2021). Mice were administered normal chow or a diet containing 0.2% adenine for 4 weeks. Macrophages have been implicated in kidney development, including the renal vasculature (Alikhan et al., 2011; Munro et al., 2019; Rae et al., 2007). Despite profound macrophage deficiency, the kidneys of control male and female adult wild-type and *Fireko* mice were of similar size (Fig. S8B). Consistent with previous literature (Jean-Faucher et al., 1987), the kidneys of male mice were 30-40% larger than those of females, regardless of *Csf1r* genotype (Fig. S8B). Kidneys of wild-type and *Fireko* mice were not distinguished based upon evaluation of Haematoxylin and Eosin (H&E)-, periodic-acid-Schiff (PAS)- or Sirius-red-stained sections by a veterinary pathologist (R.A.) who was unaware of the genotype (Fig. S8C). *Cx3cr1* mutation or macrophage depletion led to accumulation of intratubular sediments in the medulla detectable by H&E or PAS staining (He et al., 2024). No such lesions were detected in *Fireko* control mice. Despite the paucity of F4/80^high^ macrophages in the *Fireko* kidneys at steady state, adenine injury promoted massive interstitial accumulation of F4/80^+^ macrophages in cortex and medulla in both wild-type and *Fireko* mice (Fig. S8C). Consistent with Fig. S7E, the *Fireko* mutation had no effect on markers of renal function, i.e. serum creatinine or urea, in the control chow-fed male or female mice (Fig. S8E). All adenine treatment groups lost weight but there was no difference between genotypes in body weight or kidney weight (Fig. S8A,B). Kidneys from adenine-treated mice exhibited severe tubular pathology (atrophy, adenine crystal, and cast and/or debris deposition), milder glomerular pathology (Bowman's capsule thickening) and fibrosis (Fig. S8D), which are associated with increased circulating creatinine and urea (Fig. S8E). Disease severity was indistinguishable between genotypes histologically, but circulating creatinine and urea levels in adenine-treated male *Fireko* mice were increased to a greater extent compared to wild type (Fig. S8E). We conclude that neither resident renal macrophages nor CSF1R expression in marrow progenitors or classical monocytes is absolutely required in the response to renal injury.

### Cardiac macrophage deficiency in *Fireko* mice does not affect steady-state heart function

The abundant tissue macrophages in the heart have been ascribed essential functions in electrical conductivity and metabolic homeostasis, with conditional depletion of macrophages leading to changes in cardiac haemodynamics and ventricular dysfunction within 3 weeks of treatment (Hulsmans et al., 2017; Nicolás-Ávila et al., 2020). We localized cardiac resident macrophages using IBA1 in combination with CD169 and CD206, which have been reported to define subpopulations of cardiac macrophages (Wong et al., 2021). Those populations were depleted in the *Fireko* mouse (Rojo et al., 2019). As observed in the kidney, the resident cardiac macrophages were also deficient in older *Csf1r*^ΔFIRE/+^ mice (Fig. S9A). To determine the effect of cardiac macrophage deficiency, we examined cardiac function in 13- to 19-week-old animals by cardiac ultrasound (Nicolás-Ávila et al., 2020). As shown in Fig. S9B, none of the measured parameters was significantly different between wild-type and *Fireko* mice.

### Vacant tissue macrophage niches are populated by donor cells following adoptive transfer of wild-type bone marrow cells

Taking advantage of the inbred genetic background, wild-type, *Csf1r*^ΔFIRE/+^ and *Fireko* mice with the *Csf1r-*EGFP transgene were injected intraperitoneally with wild-type bone marrow cells (bone marrow transfer, BMT) from *Csf1r-*FusionRed (FRed^+^) donors at weaning (3 weeks). Chimerism was assessed 12 weeks post BMT. In the periphery, whole-mount imaging revealed the presence of donor FRed^+^ macrophages within the vacant territories identified in the *Fireko* mutation recipients. Selected tissues are shown in Fig. 6 and Fig. S10. Surprisingly, although adipose, pancreas, ovary, diaphragm and salivary gland in adult *Fireko* mice retained populations of F4/80^+^/IBA1^+^ macrophages, in the BMT recipients, macrophages with a similar density and stellate morphology were entirely of donor (FRed^+^) origin. In the testis, we observed replacement of peritubular macrophages as well as foci of FRed^+^ interstitial macrophages (Fig. 6C).

In the liver, donor cells were detected amongst the capsular population, whereas underlying Kupffer cells remained of recipient origin (EGFP^+^, Fig. 6B). Similarly, in the lung, consistent with the selective reduction of interstitial *Csf1r-*GFP^+^ cells at weaning (Fig. 5C), donor cells populated the stellate interstitial population, whereas, consistent with their retention in adult *Fireko* mice (Rojo et al., 2019), the putative alveolar macrophages identified by a more-rounded morphology and location within the airways remained EGFP^+^ (Fig. S10). We also detected donor cells on the surface of the spleen, whereas the red pulp macrophages remained EGFP^+^ (Fig. S10). Importantly, no FRed^+^ cells were detected by flow cytometry analysis of CD11b^+^ myeloid cells in bone marrow, blood or spleen cells of transplanted *Fireko* mice (Fig. 6G).

**Fig. 7.**
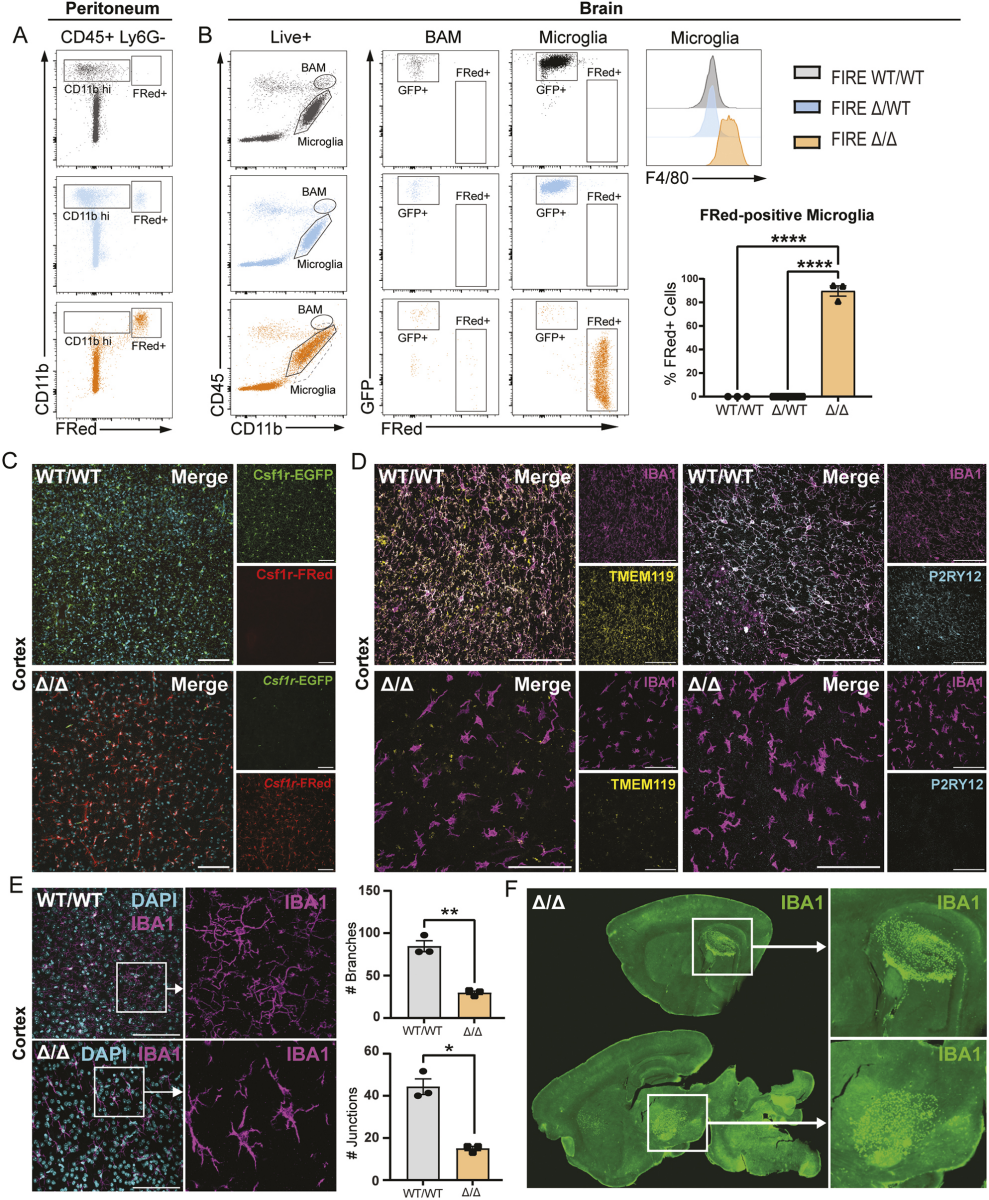
**Repopulation of the brain of *Fireko* mice with wild-type donor cells following bone marrow transfer.** *Fireko*/*Csf1r-*EFGP mice were injected intraperitoneally with *Csf1r*-FusionRed (Fred) donor bone marrow cells at weaning (3 weeks), and engraftment was analysed 12 weeks post-transfer. (A) Representative flow cytometry profiles of peritoneal cells harvested from wild-type (+/+), *Csf1r*^ΔFIRE/+^ (Δ/+) and *Fireko* (Δ/Δ) recipients. (B) Representative flow cytometry profiles of brain myeloid cells harvested from the same cohort. The dotted line in the CD45/CD11b profile highlights the shift in location of the microglial gate from wild-type to *Fireko* bone marrow transfer (BMT) recipients. Proportions of FRed^+^ microglia are quantified on the right. Data are mean±s.e.m. from at least three mice per genotype. *P* values were determined using ordinary one-way ANOVA followed by Tukey's multiple comparison test (****P<0.0001). Histogram panel shows F4/80 staining on microglial cells isolated from BMT recipients. (C) Representative images of sections of brain cortex from wild-type (+/+) and *Fireko* (Δ/Δ) BMT recipients show the repopulation of the cortex with donor (FRed^+^ cells). Scale bars: 100 μm. (D) Representative images of sections of brain cortex from wild-type (+/+) and *Fireko* (Δ/Δ) BMT recipients stained for IBA1 (magenta), TMEM119 (yellow) and P2RY12 (teal). Scale bars: 100 μm. (E) Representative images of sections of brain cortex stained for IBA1 (magenta) and co-stained with DAPI. Highlighted regions (outlined) are shown at higher resolution on the right to highlight the morphological difference between wild-type microglia and microglia repopulated in *Fireko* mice. Microglial ramification was quantified in ImageJ. Data are mean±s.e.m. of three mice per genotype. *P* values were determined using an unpaired *t*-test with Welch's correction. *P<0.05; **P<0.01. Scale bars: 100 μm. (F) *Fireko* (Δ/Δ) recipients were sacrificed at 3 weeks post BMT. Serial free-floating sections were stained to detect repopulation with IBA1^+^ cells. Every section examined contained multiple foci of IBA1^+^ cells, panels show typical examples.

The CD11b^high^ large peritoneal macrophage population, which is absent in *Fireko* mice, was populated by FRed^+^ donor cells (Fig. 7A). No donor cells were detected in any organ in wild-type recipients (Fig. 6). However, in heterozygous recipients, partial chimerism was detected in the peritoneum (Fig. 7A) and in organs with direct access to the peritoneal cavity (diaphragm, adipose, pancreas and liver capsule; Fig. S10B). In the *Fireko* kidney, F4/80^high^ resident macrophages are selectively depleted, whereas F4/80^low^ populations are retained (Rojo et al., 2019). Each of these populations expresses FRed in the donor, but in BMT recipients only the F4/80^high^ macrophages were of donor origin (Fig. S10C).

The brain is of particular importance because of the neuropathology associated with *CSF1R* mutations in humans (Chitu et al., 2022) and the potential for treatment by microglial replacement. The brains of *Fireko* mice were populated with FRed^+^ donor-derived cells 12 weeks post-BMT (Fig. 7B,C). No residual *Csf1r*-EGFP^+^ cells were detected in the brain parenchyma of BMT recipients, indicating that recipient border-associated macrophages (BAMs) were also replaced. No FRed^+^ donor cells were detected in the brains of wild-type or heterozygous BMT recipients (Fig. 7B). Previous evidence indicated that bone marrow-derived microglia-like cells do not fully recapitulate homeostatic gene expression (Bastos et al., 2025; Bennett et al., 2018; Carter-Cusack et al., 2024; Cronk et al., 2018; Lund et al., 2018; Shemer et al., 2018; Shibuya et al., 2022). In BMT recipients, FRed^+^ donor-derived cells had relatively increased CD45 and F4/80 expression compared to wild-type microglia (Fig. 7B). Donor-derived cells were uniformly strongly FRed^+^ and achieved a similar density and regular distribution to wild-type microglia. However, they did not replicate the extensive ramification characteristic of normal microglia (Fig. 7C) and expression of homeostatic microglia markers P2RY12 and TMEM119 was barely detectable in the donor-derived cells (Fig. 7D). The difference in morphology between resident and BM-derived cells in the cortex is shown in more detail by IBA1 staining and quantitative image analysis in Fig. 7E. To gain insight into the mechanism of repopulation of the microglial niche, we examined brains at 3 and 5 weeks post BMT. Within individual brains, we observed multiple large clusters of IBA1^+^ cells with a concentrated ‘wave front’ similar to the pattern detected following intracerebral microglial transplantation (Chadarevian et al., 2024) (Fig. 7F).

## DISCUSSION

### Selective loss of resident tissue macrophage populations in *Fireko* mice

The *Csf1r*-EGFP transgene (Sasmono et al., 2003) is expressed by all myeloid cells and some B cells. In most tissues it is co-expressed with F4/80 and provides a convenient marker to enable visualization of tissue mononuclear phagocytes. In the current study, we combined the *Csf1r-*EGFP reporter with the *Csf1r*^ΔFIRE^ allele on the congenic C57BL6/J background to extend the previous analysis of *Fireko* mice by flow cytometry on disaggregated tissues (Rojo et al., 2019). In the kidney, we confirmed the absence of F4/80^high^ resident macrophages in the cortex and the more abundant population lining the tubules in the medulla (Hume and Gordon, 1983). Based upon the loss of both *Csf1r-*EGFP and F4/80, several additional tissue macrophage populations were found to be depleted in the tissues of *Fireko* mice, including subpopulations in liver, lung, skeletal muscle, testis, ovary, pituitary, adrenal and pancreas. The testis contains two distinct macrophage populations occupying interstitial and peritubular locations (DeFalco et al., 2015; Gu et al., 2023, 2022; Mossadegh-Keller et al., 2017). The CSF1R^low^ population was selectively lost in the mutant mice, confirming that the peritubular cells occupy a separate niche and that the two testis populations are regulated separately. The lung contains an interstitial macrophage population that is enriched in the periphery of the organ (Tan and Krasnow, 2016) and is readily imaged in whole mounts. This population was selectively depleted in the juvenile *Fireko* mice. We speculate that the perinatal lethality associated with *Csf1r* mutations on the C57BL/6J background (Chitu and Stanley, 2017) may be related to neonatal lung macrophage deficiency and a consequent defect in the alveoli (Abe et al., 2025; Jones et al., 2013).

The original description of the *Fireko* mice described the loss of epidermal LCs in the ear skin. In thicker stratified squamous epithelia, LCs spread in three dimensions through multiple layers of epidermal cells. We found that sustained loss of LCs in the *Fireko* mice was specific to the epidermis of the ear, where LCs are located in the basal cell layer of individual squame piles (Hume et al., 1983). This may relate in part to ontogeny. Mass et al. (2023) have reviewed evidence of a monocytic contribution to the generation and maintenance of LCs and dermal macrophages in different locations.

### The heterozygous phenotype in *Csf1r^ΔFIRE/+^* mice indicates quantitative CSF1R signalling

The rapid postnatal expansion of resident macrophage population in all mouse tissues is detectable in total tissue transcriptomic profiles (Summers and Hume, 2017). This expansion is CSF1/CSF1R dependent (Stables et al., 2022). Here, we have found that heterozygous *Csf1r*^ΔFIRE^ mutation was sufficient to compromise postnatal expansion of macrophage populations in tissues affected by the homozygous mutation (brain, kidney and heart). These findings indicate that receptor availability is limiting for response. This, in turn, depends on continuous replacement of CSF1R on the surface as ligand-bound receptor is internalized and degraded (Stanley and Chitu, 2014). The highly conserved AP1 element of FIRE contributes to the kinetics of receptor re-expression, paralleling the requirement for a similar conserved ETS/AP1 element in an enhancer of the CSF1R target gene *Plau* (Fowles et al., 1998). The homozygous AP1 mutation led to a reduction in the rate of re-appearance of CSF1R following growth factor removal and reduced CSF1-dependent proliferation. The transient macrophage depletion seen in juvenile heterozygotes cannot be sustained because of the simple homeostatic feedback mechanism; reduced numbers of CSF1R^+^ macrophages consume less of the available local growth factor (Sehgal et al., 2021). The continued depletion of macrophages in kidney and heart in heterozygous mice may indicate that they depend on circulating rather than local CSF1. In the context of CSF1R-related leukoencephalopathy, the results do indicate a possible impact of haploinsufficiency that could interact with genetic background or an environmental trigger to initiate disease (Hume, 2025).

### Mechanisms contributing to niche-specific loss of tissue-resident macrophages

In locations where the F4/80^high^ resident population is depleted in *Fireko* mice, the residual macrophage populations are predominantly F4/80^low^ monocyte-derived cells (McKendrick et al., 2023; Rojo et al., 2019; Weinberger et al., 2024). The kidney, which, like the brain, is macrophage deficient in *Fireko* mice, is one clear exception, with embryonic macrophage populations being replaced by bone marrow-derived cells in the first few weeks of life (Salei et al., 2020). *Fireko* mice are macrophage deficient in the embryo (Munro et al., 2020). Even those organs that retain macrophages in adult *Fireko* mice, such as liver, lung, intestine and spleen, are macrophage deficient in the embryo and remain partly macrophage deficient at 3 weeks of age. Delayed development of resident macrophages in *Fireko* mice may explain why donor cells transferred at weaning were able to populate organs that were not macrophage-deficient in adults (Fig. 6).

*Fireko* mice lack *Csf1r* expression in bone marrow progenitors and monocytes, and are unresponsive to CSF1 *in vitro* or *in vivo* but they are not monocyte deficient in marrow or blood. This observation is consistent with previous evidence that a blocking anti-CSF1R antibody does not deplete monoblasts in the marrow (Sudo et al., 1995) or blood monocytes (Louis et al., 2015; MacDonald et al., 2010), and the lack of effect of conditional *Csf1* deletion on marrow monocytes (Emoto et al., 2022; Thierry et al., 2024). Blood monocyte subsets defined by markers such as Ly6C, CCR2 and CX3CR1 have been considered to be a developmental series that depends upon CSF1R signalling (Guilliams et al., 2018; Thierry et al., 2024). Ly6C^high^ classical monocytes may regulate the survival of their Ly6C^low^ progeny by acting as a sink for CSF1 (Yona et al., 2013). Treatment with anti-CSF1R antibody in mice leads to the loss of non-classical monocytes and accumulation of classical monocytes (Louis et al., 2015; MacDonald et al., 2010). However, despite the almost complete loss of CSF1R in progenitors and monocytes in bone marrow, the relative abundance of circulating Ly6C^low^ monocytes was not reduced in *Fireko* mice. Expression of the *Csf1r-*FRed reporter is around fivefold higher in Ly6C^low^ compared to Ly6C^high^ monocytes (Grabert et al., 2020). The Ly6C^low^ monocytes had detectable, albeit reduced, CSF1R expression, when incubated without CSF1 *in vitro*. Both decreased CSF1R and an increased abundance of Ly6C^low^ monocytes are likely due to the absence of CSF1R on Ly6C^high^ monocytes effectively removing the competition for endothelial CSF1 (Thierry et al., 2024). The macrophage populations of the major organs that retain tissue macrophages in *Fireko* mice, i.e. liver, spleen, intestine and lung, are also partly retained in *Csf1r* knockout mice (Dai et al., 2002; Jacquelin et al., 2026) and rats (Carter-Cusack et al., 2024; Keshvari et al., 2021), suggesting that these organs provide factors that can maintain survival and induce CSF1R expression in infiltrating monocytes. CSF2 in lung and intestine is one such factor (Kvedaraite et al., 2024; van de Laar et al., 2016; Varol et al., 2009); intestinal CSF2 in portal blood could also contribute to the retention of liver Kupffer cells. Surprisingly, the analysis in Fig. 1G indicates that F4/80^+^ hematopoietic island macrophages are also retained and can express CSF1R, in contrast to Ly6C^high^ marrow monocytes in the same tissue environment. Two recent studies have provided evidence that conditional deletion of *Csf1* expression in marrow adipogenic stromal cells leads to loss of osteoclasts and resident macrophages without affecting monocytes (Inoue et al., 2023; Zhong et al., 2023).

### The absence of tissue-resident macrophages in specific organs does not compromise development and homeostasis

Macrophages have been attributed numerous functions in embryonic and postnatal development and physiology (Mass et al., 2023; Matsudaira and Prinz, 2022; McNamara et al., 2023; Zhao et al., 2024). Thus far, analysis of the *Fireko* mice has not revealed any essential physiological function of resident macrophages. In contrast to the infertility of *Csf1* and *Csf1r* mutant mice (Dai et al., 2002), and despite loss of macrophages in the gonads, *Fireko* mice are fertile. Renal medullary macrophages were recently shown to have a role in monitoring urine contents to prevent accumulation of sedimentary particles (He et al., 2024). We found no histological evidence of spontaneous tubular blockage or renal dysfunction in macrophage-deficient *Fireko* mice, nor was there any increase in tubular lesions in the adenine diet model (Fig. S7D). We also did not replicate the reported impact of macrophage depletion on cardiac function (Hulsmans et al., 2017; Nicolás-Ávila et al., 2020). Whereas depletion of tissue macrophages leads to alteration in lipid storage (Cox et al., 2021) and adrenal macrophages have been implicated in metabolic homeostasis (Dolfi et al., 2022; Xu et al., 2024), the body composition of adult *Fireko* mice was no different from wild type (Fig. S2A). Rather, resident macrophages appear to be essential for mitigating age-dependent neuropathology (Chadarevian et al., 2024; Kiani Shabestari et al., 2022; Munro et al., 2024) or tissue injury, e.g. in radiation-induced injury in the salivary gland or in myocardial ischemia models (McKendrick et al., 2023; Weinberger et al., 2024). This role was less evident in the acute renal injury model in the current study. Further studies will focus on possible functions in recovery and repair (Alikhan et al., 2011; Cochrane et al., 2005).

### Repopulation of vacant territories in *Fireko* mice by wild-type bone marrow cells

The vacant macrophage territories of *Fireko* mice were occupied selectively by donor cells following intra-peritoneal wild-type bone marrow cell transfer at weaning. Donor cells do not make any detectable contribution to bone marrow or circulating blood monocytes in this model. Repopulation of a vacant microglial niche has previously been demonstrated following neonatal adoptive transfer of monocytes purified from bone marrow (Bastos et al., 2025; Bennett et al., 2018). Based upon these observations and on related findings in rats (Carter-Cusack et al., 2024; Keshvari et al., 2021; Sehgal et al., 2023) and chickens (Garceau et al., 2015) it seems likely that bone marrow contains a CSF1R-dependent macrophage progenitor population that gives rise directly to resident tissue macrophages. Future experiments will explore the relationship to the clonogenic monocyte and/or macrophage-restricted progenitor defined by Hettinger et al. (2013). Hematopoietic stem and progenitor cells and/or committed progeny have been shown to migrate in the circulation and lymphatics, and to be capable of giving rise directly to tissue-resident myeloid cells (Massberg et al., 2007). One interesting question is whether the macrophage-depleted peritoneal cavity provides a unique trophic niche for expansion of these cells that might be exploited therapeutically.

### Conclusions

Analysis of the *Fireko* mice has revealed surprising levels of redundancy in mononuclear phagocyte biology, in the function of the enhancer, in the expression of CSF1R in progenitors and monocytes, and in the roles of resident macrophages in tissue development and homeostasis. It is likely that tissue-resident macrophages will prove to be more important to innate and acquired immune responses and repair following tissue injury.

## MATERIALS AND METHODS

### Animals

The 418 bp deletion containing the Fms intronic regulatory element (FIRE) to produce the *Csf1r*^ΔFIRE^ allele was generated using CRISPR-Cas9 in C57Bl/6J×CBA/J F1 zygotes (Rojo et al., 2019). The allele was backcrossed for 10 generations to C57BL/6J, then transferred to Australia, where it was crossed to *Csf1r*-EGFP reporter mice (Sasmono et al., 2003) on the C57Bl/6J/Arc background. Residual non-C57BL/6J contributions to the genome in this line were found to partly mitigate perinatal lethality and hydrocephalus (Taylor et al., 2025 preprint). To restore resilience and generate larger cohorts for functional analysis, *Csf1r*^ΔFIRE/+^ mice were mated with wild-type CBA/J mice (Animal Resource Centre/WA) and F1 mice were crossed to generate homozygotes (*Fireko*) on the F2 background. The *Csf1r-*FusionRed knock-in reporter transgenic line has been described elsewhere (Grabert et al., 2016) and is also maintained on the C57Bl/6J/Arc background.

We used CRISPR/Cas9 to mutate the AP1-binding site in the FIRE sequence within intron 2 of the mouse *Csf1r* gene. The site is located at Chr18:6124034 (GRCm39). A sgRNA was designed, aactgattcacagcctctga-ggg, that directly targets the AP1 tgattca motif and a ssODN donor (tgccagcaatgtgtttccgcccacacaggccgggggcgcctgccaggccctcagaggctgTCTAGAgttctcacttccccccttcccccctatttcaagcctgggaaaaatgctgacaccacacag) with 60 nt homology flanking a TCTAGA sequence to substitute this AP1 site. In addition to destroying the AP1 site, this integrates an XbaI restriction enzyme site used for rapid genotyping of founders.

The sgRNA was produced as an Alt-R crRNA (IDT) oligo and resuspended in sterile RNase free injection buffer [Tris HCl 1 mM (pH 7.5) and EDTA 0.1 mM] and annealed with trans-activating crispr RNA (tracrRNA; IDT) by combining 2.5 μg crRNA with 5 μg tracrRNA and heating to 95°C. The mix was left to slowly cool to room temperature. After annealing the complex, an equimolar amount was mixed with 1000 ng Cas9 recombinant protein (NEB; final concentration 20 ng/μl) and incubated at room temperature for 15 min, before adding Cas9 messenger RNA (mRNA) (final concentration 20 ng/μl) and the ssODN polyacrylamide gel electrophoresis (PAGE) purified repair template (IDT; final concentration 50 ng/μl) in a total injection buffer volume of 50 μl. The injection mix was centrifuged for 10 min at room temperature and the top 40 μl removed for microinjection.

The mix was microinjected into 1-day-old single-cell mouse embryos (C57BL/6JOlaHsd). The zygotes were cultured overnight and the resulting 2-cell embryos implanted into the oviduct of day 0.5 post-coitum pseudopregnant mice. Genotyping was performed using the Sigma redextract-n-amp tissue PCR kit. In brief, ear clips were digested, then PCR was performed at an annealing temperature of 62°C with forward (5′ ctgtcactgtgtaggaagggt) and reverse primers (5′ gaaaccgttgtgtcatcccc). The DNA was then digested with 2 μl CutSmart Buffer (NEB, R0137L) and 1 μl XbaI enzyme (NEB) at 37°C for 1 h. The products were analysed on a Qiaxcel automated gel system. A candidate founder was Sanger sequenced to confirm the desired genetic change, and a colony established and transferred to Australia.

All animal experiments in Australia were approved by The University of Queensland Health and Sciences Ethics Committee and performed in accordance with the Australian Code of Practice for the Care and Use of Animals for Scientific Purposes and Queensland Animal Care and Protection Act (2001). Animals were housed in individually ventilated cages with a 12 h light/dark cycle, and food and water available *ad libitum*. For animal breeding in the UK, ethical approval was obtained from The Roslin Institute's and The University of Edinburgh's Protocols and Ethics Committees, under the authority of a UK Home Office Project License under the regulations of the Animals (Scientific Procedures) Act 1986.

### Cell isolation and culture

Peripheral blood was collected into EDTA-coated tubes via cardiac puncture and analysed using an automated haematology analyser, Mindray BC5000 Haematology Analyser (TRI Flow Cytometry Facility). Blood was then subjected to red blood cell lysis for 2 min in lysis buffer [150 mM NH_4_Cl, 10 mM KHCO_3_ and 0.1 mM EDTA (pH 7.4)], followed by two PBS washes and resuspension in FACS buffer (1×PBS with 2% FBS) for staining. Peritoneal cells were collected by lavage with 5 ml of PBS, centrifuged at 400 ***g*** for 5 min at 4°C, and resuspended in 1 ml of FACS buffer for staining. To generate elicited macrophages, a 4% sterile thioglycollate broth solution (Brewer) was prepared and administered as a single intraperitoneal (i.p.) injection on day 1. The control group received an equivalent volume of sterile saline via i.p. injection. Peritoneal cells were harvested by lavage on day 5.

Bone marrow (BM) cells were prepared by flushing femurs and tibias with 10 ml of PBS into a tube on ice, then centrifuging at 400 ***g*** for 5 min at 4°C. The pellet was resuspended in 1 ml of FACS buffer for staining. Following red cell lysis, single-cell suspensions were stained with fluorophore-conjugated antibodies on ice and analysed on an LSR Fortessa, (BD, Australia). Cell-surface markers used to define hematopoietic cell types and antibodies are provided in Table S1. Data were analysed using FlowJo (BD). Cell counts were determined using a Mindray BC5000 Haematology Analyser (TRI Flow Cytometry Facility).

For bulk cultures to generate bone marrow-derived macrophages (BMMs), ∼10^7^ BM cells were seeded on 100 mm square bacteriological plastic (Sterilin, Thermo-Fisher, Australia) in 25 ml of complete medium [RPMI+10% FBS, 25 U/ml penicillin and 25 μg/ml streptomycin (Gibco, Thermo-Fisher)] and differentiated for 7 days with the addition of recombinant CSF1-Fc (100 ng/ml) or recombinant mouse CSF2 (GM-CSF, R&D Systems; 50 ng/ml). For osteoclast culture, bone marrow cells were harvested as described above and seeded at 5×10^4^ cells/ml in 12-well tissue-culture plates in complete medium with CSF1 (100 ng/ml) for 3 days. From day 3 the medium was replaced daily with medium containing CSF1-Fc (100 ng/ml) and RANKL (R&D Systems, 40 ng/ml) until day 7. Tartrate-resistant acid phosphatase (TRAP) staining was performed *in situ* using a Leukocyte Acid Phosphatase (TRAP) kit (387A, Sigma) according to the manufacturer's instructions.

### Whole-mount imaging

Whole-mount imaging of the *Csf1r*-EGFP and *Csf1r-*FusionRed reporters was performed using a FV3000 microscope (Olympus) and FLUOVIEW (Olympus) software. Tissue was harvested and kept in ice-cold PBS until imaging. Tissue was placed flat on a glass petri dish. *Z*-stack images were taken using the AF488 and AF594 lasers, and combined to produce maximum intensity projections. Images were taken at 10× or 20× magnification and processed on ImageJ software v1.54.

### Immunofluorescence staining

For cryosections, spleens were dissected and immersion fixed in 4% PFA for 4 h, before cryoprotection in 15% sucrose in PBS overnight and then 30% sucrose in PBS for 48 h. Spleens were then embedded in OCT, frozen and sectioned at 10 μm using a ThermoFisher HM525NX cryostat. For staining, sections were rehydrated in Tris-buffered saline [TBS; 10 mM Tris and 150 mM NaCl (pH8.0)] for 10 min and washed three times for 1 min each in TBST (0.01% Tween-20 in TBS). Blocking buffer (3% BSA and 5% serum in TBST) was applied for 30 min at room temperature, before sections were incubated with the primary antibodies rat anti-CD169 (1:100, Biolegend, 142402) and rabbit anti-CD209B (1:150, Abcam, ab308457) diluted in TBST for 1.5 h at room temperature. Sections were washed three times for 5 min each in TBST before incubation in the secondary antibodies donkey anti-rabbit AF647 (1:500, Invitrogen, A31573) and goat anti-rat AF594 (1:400, Invitrogen, A11007) diluted in TBST for 45 min protected from light. Sections were washed three times for 5 min each in in PBS, stained with DAPI and mounted for imaging.

### F4/80 immunohistochemistry

10 μm paraffin sections were dewaxed, rehydrated and incubated for 30 min in 10 mM Tris/EDTA (pH 9) buffer maintained at 90-100°C using a microwave for antigen retrieval. Slides were cooled on ice for 10 min, incubated in 0.3% hydrogen peroxide (Sigma) for 15 min to block endogenous peroxidases and washed in TBS. Slides were incubated in 1% BSA in TBS for 30 min followed by incubation with primary antibody (anti-F4/80, 1:1000, rabbit, Abcam) for 1.5 h in a humidified chamber. Slides were washed with TBS, incubated sequentially with HRP secondary reagent (anti-rabbit, Agilent) for 30 min, DAB peroxidase substrate (Agilent) for 5 min and Haematoxylin for 40 s, then dehydrated and mounted. Imaging was performed on an Olympus BX50 microscope, using bright-field settings.

### Quantification of mRNA expression

Tissues for qRT-PCR analysis were snap frozen on dry ice and stored in a −80°C freezer. Approximately 50-100 mg of frozen tissue was homogenized in 1 ml of ice-cold TRIzol reagent using a FastPrep homogenizer (40 s at 5 m/s). The homogenates were centrifuged at 12,000 ***g*** for 5 min at 4°C, and the clear supernatant was transferred to a fresh tube. Following a 5 min incubation to dissociate nucleoprotein complexes, 0.2 ml of ice-cold chloroform was added per ml of TRIzol reagent, and the mixture was inverted 5-10 times, incubated for 3 min and centrifuged at 12,000 ***g*** for 15 min at 4°C. The RNA-containing aqueous phase was carefully transferred to a new tube, mixed with 0.5 ml of isopropanol and incubated for 10 min to precipitate RNA. The RNA pellet was washed twice with 75% ethanol, air-dried for 5-10 min and dissolved in 20-50 μl of RNase-free water. Dissolved RNA was incubated at 55-60°C for 10-15 min to ensure complete solubilization. RNA quantity was measured using a NanoDrop Spectrophotometer (ThermoFisher Scientific).

Complementary DNA (cDNA) was synthesised from 1 μg of total RNA using the SensiFAST cDNA Synthesis Kit (Bioline). RNA was first treated with DNase I (Sigma) to remove genomic DNA contamination by incubating the RNA with DNase I (0.2 μl) and 10× buffer (1 μl) in a total volume of 10 μl at room temperature for 15 min, followed by inactivation with 1 μl of 25 mM EDTA at 65°C for 10 min. Reverse transcription was performed in a 20 μl reaction containing 11 μl RNA, 4 μl 5× TransAmp buffer, 0.25 μl reverse transcriptase and 4.75 μl RNase-free water. Reactions were incubated at 24°C for 10 min (primer annealing), 42°C for 15 min (reverse transcription) and 85°C for 5 min (enzyme inactivation). The resulting cDNA was diluted 1:10 with RNase-free water for subsequent quantitative PCR analyses and stored at −20°C.

Gene expression analysis was performed using quantitative real-time PCR (qRT-PCR) with SYBR Green PCR Master Mix. Each reaction was conducted in a 10 μl volume containing 2 μl of diluted cDNA, 2 μl of forward and reverse primer mix (1-2 μM final concentration), 5 μl of SYBR Green Master Mix and 1 μl of RNase-free water. Thermal cycling was carried out on a real-time PCR system with the following conditions: initial denaturation at 95°C for 2 min, followed by 40 cycles of 95°C for 15 s and 60°C for 1 min, and a melt curve analysis to confirm specificity. Relative expression levels of target genes were normalised to the expression of a housekeeping gene using the ΔΔCt method. All reactions were performed in triplicate to ensure reproducibility.

Primer sequences used are provided in Table S2.

### Treatment with CSF1 *in vivo*

To assess the *in vivo* response to CSF1, mice were injected with a recombinant human CSF1-mouse Fc conjugate provided by Novartis (Switzerland). In this protein, the N-terminal 36-190 amino acids of human CSF1 encoding the biologically active protein are fused via a flexible linker to the Fc region of mouse IgG2A containing the L234A/L235A mutation to reduce Fc receptor binding (Arduin et al., 2015). Mice received one injection per day of 1 mg/kg of CSF1-Fc for 4 days between Zeitgeber time (ZT) 2-3. Mice were placed in the Minispec LF5OH (Brucker) at ZT2 to measure their body composition and blood glucose prior to the first injection. Their body weight was monitored daily. On day 5, body composition and blood glucose were again measured, and mice were euthanised with CO_2_ prior to tissue collection.

### Thioglycollate-elicited peritoneal macrophages

1 ml of thioglycolate broth was injected into the peritoneal cavity of adult male wild-type and *Fireko* mice. Peritoneal cells were harvested by lavage after 5 days.

### Adoptive transfer of wild-type bone marrow cells

Bone marrow cells were harvested from the tibia and femur of *Csf1r*-FusionRed female mice. The bones were flushed with a solution containing 0.9% sodium chloride and 2% heat-inactivated FBS. The cell suspension was then filtered through a 70 μm cell strainer and centrifuged at 500 ***g*** for 5 min at 5°C. The pelleted cells were resuspended in 1× sterile PBS supplemented with 2% heat-inactivated FBS (10 ml per bone) for cell counting using a haemocytometer (Reichert, USA). Following counting, cells were centrifuged and resuspended in 1×sterile Dulbecco's phosphate-buffered saline (DPBS, Gibco, 10010-023). 2×10^7^ *Csf1r*-FusionRed cells were injected i.p. at weaning and recipients were analysed 12 weeks post transfer.

### Adenine chronic kidney disease model

Male and female 8-week-old wild-type and *Fireko* mice were exposed to 0.2% adenine in the drinking water for 4 weeks as described previously (Diwan et al., 2018). Bodyweights were measured weekly. At the point of euthanasia, blood was taken for measurement of urea, creatinine and phosphate by University of Queensland Veterinary Pathology, and tissues processed for histology and immunohistochemistry.

Kidneys were fixed in 4% PFA for 4 h, dehydrated and embedded in paraffin wax. Thin sections (5 μm) of the kidney were cut and stained with H&E. An Olympus VS120 Slidescanner Microscope (TRI) was used for imaging, and image morphometry was analysed by a veterinary pathologist (Dr Rachel Allavena), who was unaware of sample identity. Percent tubulointerstitial atrophy and percent tubules with casts/cell debris were assessed morphometrically in four fields/kidney at ×20 digitally scanned magnification. Apoptosis was counted using structural characteristics in the tubular epithelium of four fields/kidney at ×20 digitally scanned magnification. The inflammatory infiltrate was assessed on a 4 point scale: 1=normal; 2=little infiltrate (<10% of field), 3=moderate infiltrate (10–50% of field) and 4=extensive infiltrate (>50% of field, and usually ≫50%/field). Glomerular pathology (sclerosis and thickening of Bowman's capsule) was assessed as the mean of individual glomeruli from four scanned cortical fields per kidney section. Loss of brush borders was assessed using periodic acid-Schiff (PAS) staining. Paraffin wax-embedded tissue sections were deparaffinized and hydrated in deionized water. Sections were incubated in periodic acid solution (1 g/dl) (Sigma Aldrich, 3951) for 5 min at room temperature and rinsed in several changes of distilled water. Slides were then immersed in Schiff's reagent (Sigma Aldrich, 3952) for 15 min at room temperature, followed by washing in running tap water for 5 min. Collagen deposition was assessed by incubating sections with a Picro-sirius Red solution (0.2 g Sirius Red F3B in 500 ml saturated picric acid) for 1 h in a glass or plastic container. After staining, sections were washed in two changes of acidified water (5 ml glacial acetic acid in 1 l distilled water) with gentle agitation (20-22 s per wash). Sections were further washed in 100% ethanol for 5 min twice to prevent cytoplasmic destaining. After dehydration through three changes of 100% ethanol, sections were cleared in xylene and mounted using DPX Mountant (Sigma Aldrich, 06522). Under bright-field microscopy, collagen appeared red on a pale yellow background, with bi-refringent collagen fibres visible under polarised light. Slides were then scanned using the Olympus VS120 Slidescanner Microscope (TRI).

### Cardiac ultrasound

Cardiac ultrasound was performed using the Vevo 2100 Imaging System. Mice were anesthetised then positioned on a platform with electrocardiogram and temperature monitoring, and their hair was hair removed for optimal imaging. Probe selection was tailored to the size of the animal and its heart (e.g. MS400 for mice), ensuring optimal image acquisition. The parasternal long and short axis views in echocardiography were utilised to visualise cardiac anatomy and assess function. The parasternal long axis (PLAX) view provided a sagittal section of the heart, revealing structures such as the left and right ventricles, left atrium, aorta, mitral and aortic valves, and pulmonary artery. This view was employed to measure parameters including ejection fraction and cardiac output. To obtain this view, the probe was positioned vertically and rotated slightly counterclockwise. In contrast, the parasternal short axis (PSAX) view offered a transverse section of the heart, enabling visualisation of the ventricles, intraventricular septum, papillary muscles and other structures. This view was achieved by rotating the probe 90° clockwise from the PLAX orientation. These complementary views formed the basis of a comprehensive evaluation of cardiac function, including wall motion, valve function and blood flow dynamics. The Simpson method was employed to assess left ventricular (LV) volumes and stroke volume by measuring the LV at multiple planes during diastole and systole. Short-axis images were acquired at three levels: near the apex (SimpAreaDist), at the mid-section near the papillary muscle (SimpAreaMid) and just below the base of the aorta (SimpAreaProx). At each level, the endocardial border was traced in both diastole and systole. Additionally, a parasternal long-axis view was obtained to measure the LV length from the aortic annulus to the endocardial border at the apex during diastole and systole. These measurements were used to calculate diastolic and systolic volumes, as well as the stroke volume, using the Vevo 2100 Imaging System's calculation tools.

## Supplementary Material



10.1242/develop.205655_sup1 Supplementary information

## References

[DEV205655C1] Abe, Y., Spann, N. J., Tang, W., Zeng, F., Seymour, C., Jansky, S., Guo, J. L., Huff, R., Chanthavixay, K., Richard, J. L. C. et al. (2025). Genetic variation in the activity of a TREM2-p53 signaling axis determines oxygen-induced lung injury. *Nat. Immunol.* 26, 1287-1298. 10.1038/s41590-025-02217-4 40715658 PMC12456008

[DEV205655C2] Alikhan, M. A., Jones, C. V., Williams, T. M., Beckhouse, A. G., Fletcher, A. L., Kett, M. M., Sakkal, S., Samuel, C. S., Ramsay, R. G., Deane, J. A. et al. (2011). Colony-stimulating factor-1 promotes kidney growth and repair via alteration of macrophage responses. *Am. J. Pathol.* 179, 1243-1256. 10.1016/j.ajpath.2011.05.037 21762674 PMC3157188

[DEV205655C3] Arduin, E., Arora, S., Bamert, P. R., Kuiper, T., Popp, S., Geisse, S., Grau, R., Calzascia, T., Zenke, G. and Kovarik, J. (2015). Highly reduced binding to high and low affinity mouse Fc gamma receptors by L234A/L235A and N297A Fc mutations engineered into mouse IgG2a. *Mol. Immunol.* 63, 456-463. 10.1016/j.molimm.2014.09.017 25451975

[DEV205655C4] Barth, M. W., Hendrzak, J. A., Melnicoff, M. J. and Morahan, P. S. (1995). Review of the macrophage disappearance reaction. *J. Leukoc. Biol.* 57, 361-367. 10.1002/jlb.57.3.361 7884305

[DEV205655C5] Bastos, J., O'Brien, C., Vara-Perez, M., Mampay, M., van Olst, L., Barry-Carroll, L., Kancheva, D., Leduc, S., Lievens, A. L., Ali, L. et al. (2025). Monocytes can efficiently replace all brain macrophages and fetal liver monocytes can generate bona fide SALL1(+) microglia. *Immunity* 58, 1269-1288.e1212. 10.1016/j.immuni.2025.04.006 40311613 PMC12094688

[DEV205655C6] Bennett, F. C., Bennett, M. L., Yaqoob, F., Mulinyawe, S. B., Grant, G. A., Hayden Gephart, M., Plowey, E. D. and Barres, B. A. (2018). A combination of ontogeny and CNS environment establishes microglial identity. *Neuron* 98, 1170-1183.e1178. 10.1016/j.neuron.2018.05.014 29861285 PMC6023731

[DEV205655C7] Calderon, B., Carrero, J. A., Ferris, S. T., Sojka, D. K., Moore, L., Epelman, S., Murphy, K. M., Yokoyama, W. M., Randolph, G. J. and Unanue, E. R. (2015). The pancreas anatomy conditions the origin and properties of resident macrophages. *J. Exp. Med.* 212, 1497-1512. 10.1084/jem.20150496 26347472 PMC4577842

[DEV205655C8] Carter-Cusack, D., Huang, S., Keshvari, S., Patkar, O., Sehgal, A., Allavena, R., Byrne, R. A. J., Morgan, B. P., Bush, S. J., Summers, K. M. et al. (2024). Wild-type bone marrow cells repopulate tissue resident macrophages and reverse the impacts of homozygous CSF1R mutation. *PLoS Genet.* 21, e1011525. 10.1371/journal.pgen.1011525 PMC11785368 39869647

[DEV205655C9] Chadarevian, J. P., Hasselmann, J., Lahian, A., Capocchi, J. K., Escobar, A., Lim, T. E., Le, L., Tu, C., Nguyen, J., Kiani Shabestari, S. et al. (2024). Therapeutic potential of human microglial transplantation in a chimeric model of CSF1R-related leukoencephalopathy. *Neuron* 112, 2686-2707.e8. 10.1016/j.neuron.2024.05.023 38897209 PMC12357648

[DEV205655C10] Chiossone, L., Chaix, J., Fuseri, N., Roth, C., Vivier, E. and Walzer, T. (2009). Maturation of mouse NK cells is a 4-stage developmental program. *Blood* 113, 5488-5496. 10.1182/blood-2008-10-187179 19234143

[DEV205655C11] Chitu, V. and Stanley, E. R. (2017). Regulation of embryonic and postnatal development by the CSF-1 receptor. *Curr. Top. Dev. Biol.* 123, 229-275. 10.1016/bs.ctdb.2016.10.004 28236968 PMC5479137

[DEV205655C12] Chitu, V., Gokhan, S., Gulinello, M., Branch, C. A., Patil, M., Basu, R., Stoddart, C., Mehler, M. F. and Stanley, E. R. (2015). Phenotypic characterization of a Csf1r haploinsufficient mouse model of adult-onset leukodystrophy with axonal spheroids and pigmented glia (ALSP). *Neurobiol. Dis.* 74, 219-228. 10.1016/j.nbd.2014.12.001 25497733 PMC4323933

[DEV205655C13] Chitu, V., Gökhan, S. and Stanley, E. R. (2022). Modeling CSF-1 receptor deficiency diseases - how close are we? *FEBS J.* 289, 5049-5073. 10.1111/febs.16085 34145972 PMC8684558

[DEV205655C14] Cochrane, A. L., Kett, M. M., Samuel, C. S., Campanale, N. V., Anderson, W. P., Hume, D. A., Little, M. H., Bertram, J. F. and Ricardo, S. D. (2005). Renal structural and functional repair in a mouse model of reversal of ureteral obstruction. *J. Am. Soc. Nephrol.* 16, 3623-3630. 10.1681/ASN.2004090771 16221872

[DEV205655C15] Cox, N., Crozet, L., Holtman, I. R., Loyher, P. L., Lazarov, T., White, J. B., Mass, E., Stanley, E. R., Elemento, O., Glass, C. K. et al. (2021). Diet-regulated production of PDGFcc by macrophages controls energy storage. *Science* 373, eabe9383. 10.1126/science.abe9383 34210853 PMC9558257

[DEV205655C16] Cronk, J. C., Filiano, A. J., Louveau, A., Marin, I., Marsh, R., Ji, E., Goldman, D. H., Smirnov, I., Geraci, N., Acton, S. et al. (2018). Peripherally derived macrophages can engraft the brain independent of irradiation and maintain an identity distinct from microglia. *J. Exp. Med.* 215, 1627-1647. 10.1084/jem.20180247 29643186 PMC5987928

[DEV205655C17] Dai, X.-M., Ryan, G. R., Hapel, A. J., Dominguez, M. G., Russell, R. G., Kapp, S., Sylvestre, V. and Stanley, E. R. (2002). Targeted disruption of the mouse colony-stimulating factor 1 receptor gene results in osteopetrosis, mononuclear phagocyte deficiency, increased primitive progenitor cell frequencies, and reproductive defects. *Blood* 99, 111-120. 10.1182/blood.V99.1.111 11756160

[DEV205655C18] DeFalco, T., Potter, S. J., Williams, A. V., Waller, B., Kan, M. J. and Capel, B. (2015). Macrophages contribute to the spermatogonial niche in the adult testis. *Cell Rep.* 12, 1107-1119. 10.1016/j.celrep.2015.07.015 26257171 PMC4545310

[DEV205655C19] Diwan, V., Brown, L. and Gobe, G. C. (2018). Adenine-induced chronic kidney disease in rats. *Nephrology (Carlton)* 23, 5-11. 10.1111/nep.13180 29030945

[DEV205655C20] Dolfi, B., Gallerand, A., Firulyova, M. M., Xu, Y., Merlin, J., Dumont, A., Castiglione, A., Vaillant, N., Quemener, S., Gerke, H. et al. (2022). Unravelling the sex-specific diversity and functions of adrenal gland macrophages. *Cell Rep.* 39, 110949. 10.1016/j.celrep.2022.110949 35705045 PMC9210345

[DEV205655C21] Emoto, T., Lu, J., Sivasubramaniyam, T., Maan, H., Khan, A. B., Abow, A. A., Schroer, S. A., Hyduk, S. J., Althagafi, M. G., McKee, T. D. et al. (2022). Colony stimulating factor-1 producing endothelial cells and mesenchymal stromal cells maintain monocytes within a perivascular bone marrow niche. *Immunity* 55, 862-878.e868. 10.1016/j.immuni.2022.04.005 35508166

[DEV205655C22] Erblich, B., Zhu, L., Etgen, A. M., Dobrenis, K. and Pollard, J. W. (2011). Absence of colony stimulation factor-1 receptor results in loss of microglia, disrupted brain development and olfactory deficits. *PLoS ONE* 6, e26317. 10.1371/journal.pone.0026317 22046273 PMC3203114

[DEV205655C23] Fonseca, G. J., Tao, J., Westin, E. M., Duttke, S. H., Spann, N. J., Strid, T., Shen, Z., Stender, J. D., Sakai, M., Link, V. M. et al. (2019). Diverse motif ensembles specify non-redundant DNA binding activities of AP-1 family members in macrophages. *Nat. Commun.* 10, 414. 10.1038/s41467-018-08236-0 30679424 PMC6345992

[DEV205655C24] Fowles, L. F., Martin, M. L., Nelsen, L., Stacey, K. J., Redd, D., Clark, Y. M., Nagamine, Y., McMahon, M., Hume, D. A. and Ostrowski, M. C. (1998). Persistent activation of mitogen-activated protein kinases p42 and p44 and ets-2 phosphorylation in response to colony-stimulating factor 1/c-fms signaling. *Mol. Cell. Biol.* 18, 5148-5156. 10.1128/MCB.18.9.5148 9710599 PMC109100

[DEV205655C25] Garceau, V., Balic, A., Garcia-Morales, C., Sauter, K. A., McGrew, M. J., Smith, J., Vervelde, L., Sherman, A., Fuller, T. E., Oliphant, T. et al. (2015). The development and maintenance of the mononuclear phagocyte system of the chick is controlled by signals from the macrophage colony-stimulating factor receptor. *BMC Biol.* 13, 12. 10.1186/s12915-015-0121-9 25857347 PMC4369834

[DEV205655C26] Geissmann, F., Manz, M. G., Jung, S., Sieweke, M. H., Merad, M. and Ley, K. (2010). Development of monocytes, macrophages, and dendritic cells. *Science* 327, 656-661. 10.1126/science.1178331 20133564 PMC2887389

[DEV205655C27] Gow, D. J., Sauter, K. A., Pridans, C., Moffat, L., Sehgal, A., Stutchfield, B. M., Raza, S., Beard, P. M., Tsai, Y. T., Bainbridge, G. et al. (2014). Characterisation of a novel Fc conjugate of macrophage colony-stimulating factor. *Mol. Ther.* 22, 1580-1592. 10.1038/mt.2014.112 24962162 PMC4435485

[DEV205655C28] Grabert, K., Michoel, T., Karavolos, M. H., Clohisey, S., Baillie, J. K., Stevens, M. P., Freeman, T. C., Summers, K. M. and McColl, B. W. (2016). Microglial brain region-dependent diversity and selective regional sensitivities to aging. *Nat. Neurosci.* 19, 504-516. 10.1038/nn.4222 26780511 PMC4768346

[DEV205655C29] Grabert, K., Sehgal, A., Irvine, K. M., Wollscheid-Lengeling, E., Ozdemir, D. D., Stables, J., Luke, G. A., Ryan, M. D., Adamson, A., Humphreys, N. E. et al. (2020). A transgenic line that reports CSF1R protein expression provides a definitive marker for the mouse mononuclear phagocyte system. *J. Immunol.* 205, 3154-3166. 10.4049/jimmunol.2000835 33139489

[DEV205655C30] Gu, X., Li, S. Y. and DeFalco, T. (2022). Immune and vascular contributions to organogenesis of the testis and ovary. *FEBS J.* 289, 2386-2408. 10.1111/febs.15848 33774913 PMC8476657

[DEV205655C31] Gu, X., Heinrich, A., Li, S. Y. and DeFalco, T. (2023). Testicular macrophages are recruited during a narrow fetal time window and promote organ-specific developmental functions. *Nat. Commun.* 14, 1439. 10.1038/s41467-023-37199-0 36922518 PMC10017703

[DEV205655C32] Guilliams, M., Mildner, A. and Yona, S. (2018). Developmental and functional heterogeneity of monocytes. *Immunity* 49, 595-613. 10.1016/j.immuni.2018.10.005 30332628

[DEV205655C33] Guo, L. and Ikegawa, S. (2021). From HDLS to BANDDOS: fast-expanding phenotypic spectrum of disorders caused by mutations in CSF1R. *J. Hum. Genet.* 66, 1139-1144. 10.1038/s10038-021-00942-w 34135456

[DEV205655C34] Guo, L., Bertola, D. R., Takanohashi, A., Saito, A., Segawa, Y., Yokota, T., Ishibashi, S., Nishida, Y., Yamamoto, G. L., Franco, J. F. d. S. et al. (2019). Bi-allelic CSF1R mutations cause skeletal dysplasia of dysosteosclerosis-pyle disease spectrum and degenerative encephalopathy with brain malformation. *Am. J. Hum. Genet.* 104, 925-935. 10.1016/j.ajhg.2019.03.004 30982609 PMC6507048

[DEV205655C35] He, J., Cao, Y., Zhu, Q., Wang, X., Cheng, G., Wang, Q., He, R., Lu, H., Weng, Y., Mao, G. et al. (2024). Renal macrophages monitor and remove particles from urine to prevent tubule obstruction. *Immunity* 57, 106-123.e107. 10.1016/j.immuni.2023.12.003 38159573

[DEV205655C36] Helft, J., Böttcher, J., Chakravarty, P., Zelenay, S., Huotari, J., Schraml, B. U., Goubau, D. and Reis e Sousa, C. (2015). GM-CSF mouse bone marrow cultures comprise a heterogeneous population of CD11c(+)MHCII(+) macrophages and dendritic cells. *Immunity* 42, 1197-1211. 10.1016/j.immuni.2015.05.018 26084029

[DEV205655C37] Hettinger, J., Richards, D. M., Hansson, J., Barra, M. M., Joschko, A. C., Krijgsveld, J. and Feuerer, M. (2013). Origin of monocytes and macrophages in a committed progenitor. *Nat. Immunol.* 14, 821-830. 10.1038/ni.2638 23812096

[DEV205655C38] Hulsmans, M., Clauss, S., Xiao, L., Aguirre, A. D., King, K. R., Hanley, A., Hucker, W. J., Wulfers, E. M., Seemann, G., Courties, G. et al. (2017). Macrophages facilitate electrical conduction in the heart. *Cell* 169, 510-522.e520. 10.1016/j.cell.2017.03.050 28431249 PMC5474950

[DEV205655C39] Hume, D. A. (2025). Life without microglia. *Trends Neurosci.* 48, 560-569. 10.1016/j.tins.2025.06.006 40651862

[DEV205655C40] Hume, D. A. and Gordon, S. (1983). Mononuclear phagocyte system of the mouse defined by immunohistochemical localization of antigen F4/80. Identification of resident macrophages in renal medullary and cortical interstitium and the juxtaglomerular complex. *J. Exp. Med.* 157, 1704-1709. 10.1084/jem.157.5.1704 6854206 PMC2186998

[DEV205655C41] Hume, D. A., Robinson, A. P., MacPherson, G. G. and Gordon, S. (1983). The mononuclear phagocyte system of the mouse defined by immunohistochemical localization of antigen F4/80. Relationship between macrophages, Langerhans cells, reticular cells, and dendritic cells in lymphoid and hematopoietic organs. *J. Exp. Med.* 158, 1522-1536. 10.1084/jem.158.5.1522 6355361 PMC2187139

[DEV205655C42] Hume, D. A., Halpin, D., Charlton, H. and Gordon, S. (1984). The mononuclear phagocyte system of the mouse defined by immunohistochemical localization of antigen F4/80: macrophages of endocrine organs. *Proc. Natl. Acad. Sci. USA* 81, 4174-4177. 10.1073/pnas.81.13.4174 6377311 PMC345391

[DEV205655C43] Hume, D. A., Pavli, P., Donahue, R. E. and Fidler, I. J. (1988). The effect of human recombinant macrophage colony-stimulating factor (CSF-1) on the murine mononuclear phagocyte system in vivo. *J. Immunol.* 141, 3405-3409. 10.4049/jimmunol.141.10.3405 3053899

[DEV205655C44] Hume, D. A., Wollscheid-Lengeling, E., Rojo, R. and Pridans, C. (2017). The evolution of the macrophage-specific enhancer (Fms intronic regulatory element) within the CSF1R locus of vertebrates. *Sci. Rep.* 7, 17115. 10.1038/s41598-017-15999-x 29215000 PMC5719456

[DEV205655C45] Hume, D. A., Irvine, K. M. and Pridans, C. (2019). The mononuclear phagocyte system: the relationship between monocytes and macrophages. *Trends Immunol.* 40, 98-112. 10.1016/j.it.2018.11.007 30579704

[DEV205655C46] Hume, D. A., Caruso, M., Ferrari-Cestari, M., Summers, K. M., Pridans, C. and Irvine, K. M. (2020). Phenotypic impacts of CSF1R deficiencies in humans and model organisms. *J. Leukoc. Biol.* 107, 205-219. 10.1002/JLB.MR0519-143R 31330095

[DEV205655C47] Hume, D. A., Millard, S. M. and Pettit, A. R. (2023). Macrophage heterogeneity in the single-cell era: facts and artifacts. *Blood* 142, 1339-1347. 10.1182/blood.2023020597 37595274

[DEV205655C48] Inoue, K., Qin, Y., Xia, Y., Han, J., Yuan, R., Sun, J., Xu, R., Jiang, J. X., Greenblatt, M. B. and Zhao, B. (2023). Bone marrow Adipoq-lineage progenitors are a major cellular source of M-CSF that dominates bone marrow macrophage development, osteoclastogenesis, and bone mass. *eLife* 12, e82118. 10.7554/eLife.82118 36779851 PMC10005769

[DEV205655C49] Irvine, K. M., Burns, C. J., Wilks, A. F., Su, S., Hume, D. A. and Sweet, M. J. (2006). A CSF-1 receptor kinase inhibitor targets effector functions and inhibits pro-inflammatory cytokine production from murine macrophage populations. *FASEB J.* 20, 1921-1923. 10.1096/fj.06-5848fje 16877523

[DEV205655C50] Jacquelin, S., Maxwell, E., Taylor, I., Green, E., O'Brien, C. J. O., Jessen, E. E., Ranpura, G., Guo, J., Nooru-Mohamed, F., Liu, Y. et al. (2026). Homozygous kinase-dead Csf1r mutation in outbred mice reveals essential and redundant functions of tissue resident macrophages. *J. Leukoc Biol.* 118, qiag074. 10.1093/jleuko/qiag074 42266149

[DEV205655C51] Jean-Faucher, C., Berger, M., Gallon, C., de Turckheim, M., Veyssière, G. and Jean, C. (1987). Sex-related differences in renal size in mice: ontogeny and influence of neonatal androgens. *J. Endocrinol.* 115, 241-246. 10.1677/joe.0.1150241 2963887

[DEV205655C52] Jones, C. V., Williams, T. M., Walker, K. A., Dickinson, H., Sakkal, S., Rumballe, B. A., Little, M. H., Jenkin, G. and Ricardo, S. D. (2013). M2 macrophage polarisation is associated with alveolar formation during postnatal lung development. *Respir. Res.* 14, 41. 10.1186/1465-9921-14-41 23560845 PMC3626876

[DEV205655C53] Keshvari, S., Caruso, M., Teakle, N., Batoon, L., Sehgal, A., Patkar, O. L., Ferrari-Cestari, M., Snell, C. E., Chen, C., Stevenson, A. et al. (2021). CSF1R-dependent macrophages control postnatal somatic growth and organ maturation. *PLoS Genet.* 17, e1009605. 10.1371/journal.pgen.1009605 34081701 PMC8205168

[DEV205655C54] Keshvari, S., Masson, J. J. R., Ferrari-Cestari, M., Bodea, L.-G., Nooru-Mohamed, F., Tse, B. W. C., Sokolowski, K. A., Batoon, L., Patkar, O. L., Sullivan, M. A. et al. (2024). Reversible expansion of tissue macrophages in response to macrophage colony-stimulating factor (CSF1) transforms systemic lipid and carbohydrate metabolism. *Am. J. Physiol. Endocrinol. Metab.* 326, E149-E165. 10.1152/ajpendo.00347.2023 38117267

[DEV205655C55] Kiani Shabestari, S., Morabito, S., Danhash, E. P., McQuade, A., Sanchez, J. R., Miyoshi, E., Chadarevian, J. P., Claes, C., Coburn, M. A., Hasselmann, J. et al. (2022). Absence of microglia promotes diverse pathologies and early lethality in Alzheimer's disease mice. *Cell Rep.* 39, 110961. 10.1016/j.celrep.2022.110961 35705056 PMC9285116

[DEV205655C56] Kvedaraite, E., Lourda, M., Mouratidou, N., Düking, T., Padhi, A., Moll, K., Czarnewski, P., Sinha, I., Xagoraris, I., Kokkinou, E. et al. (2024). Intestinal stroma guides monocyte differentiation to macrophages through GM-CSF. *Nat. Commun.* 15, 1752. 10.1038/s41467-024-46076-3 38409190 PMC10897309

[DEV205655C57] Louis, C., Cook, A. D., Lacey, D., Fleetwood, A. J., Vlahos, R., Anderson, G. P. and Hamilton, J. A. (2015). Specific contributions of CSF-1 and GM-CSF to the dynamics of the mononuclear phagocyte system. *J. Immunol.* 195, 134-144. 10.4049/jimmunol.1500369 26019271

[DEV205655C58] Louwe, P. A., Forbes, S. J., Bénézech, C., Pridans, C. and Jenkins, S. J. (2022). Cell origin and niche availability dictate the capacity of peritoneal macrophages to colonize the cavity and omentum. *Immunology* 166, 458-474. 10.1111/imm.13483 35437746 PMC7613338

[DEV205655C59] Lund, H., Pieber, M., Parsa, R., Han, J., Grommisch, D., Ewing, E., Kular, L., Needhamsen, M., Espinosa, A., Nilsson, E. et al. (2018). Competitive repopulation of an empty microglial niche yields functionally distinct subsets of microglia-like cells. *Nat. Commun.* 9, 4845. 10.1038/s41467-018-07295-7 30451869 PMC6242869

[DEV205655C60] MacDonald, K. P., Palmer, J. S., Cronau, S., Seppanen, E., Olver, S., Raffelt, N. C., Kuns, R., Pettit, A. R., Clouston, A., Wainwright, B. et al. (2010). An antibody against the colony-stimulating factor 1 receptor depletes the resident subset of monocytes and tissue- and tumor-associated macrophages but does not inhibit inflammation. *Blood* 116, 3955-3963. 10.1182/blood-2010-02-266296 20682855

[DEV205655C61] Makhloufi, C., Nicolas, F., McKay, N., Fernandez, S., Hache, G., Garrigue, P., Brunet, P., Guillet, B., Burtey, S. and Poitevin, S. (2020). Female AhR knockout mice develop a minor renal insufficiency in an adenine-diet model of chronic kidney disease. *Int. J. Mol. Sci.* 21, 2483. 10.3390/ijms21072483 32260098 PMC7177716

[DEV205655C62] Mass, E., Nimmerjahn, F., Kierdorf, K. and Schlitzer, A. (2023). Tissue-specific macrophages: how they develop and choreograph tissue biology. *Nat. Rev. Immunol.* 23, 563-579. 10.1038/s41577-023-00848-y 36922638 PMC10017071

[DEV205655C63] Massberg, S., Schaerli, P., Knezevic-Maramica, I., Kollnberger, M., Tubo, N., Moseman, E. A., Huff, I. V., Junt, T., Wagers, A. J., Mazo, I. B. et al. (2007). Immunosurveillance by hematopoietic progenitor cells trafficking through blood, lymph, and peripheral tissues. *Cell* 131, 994-1008. 10.1016/j.cell.2007.09.047 18045540 PMC2330270

[DEV205655C64] Matsudaira, T. and Prinz, M. (2022). Life and death of microglia: mechanisms governing microglial states and fates. *Immunol. Lett.* 245, 51-60. 10.1016/j.imlet.2022.04.001 35413354

[DEV205655C65] Maxwell, E., Taylor, I., Flegg, C., Liu, Y., Ranpura, G., Nooru-Mohamed, F., Green, E. K., Huang, S., Guo, J., Teakle, N. et al. (2026). A conserved upstream element in the mouse Csf1r locus contributes to transcription in hematopoietic and trophoblast cells. *J. Leukoc. Biol.* 118, qiag004. 10.1093/jleuko/qiag004 41540750

[DEV205655C66] McKendrick, J. G., Jones, G.-R., Elder, S. S., Watson, E., T'Jonck, W., Mercer, E., Magalhaes, M. S., Rocchi, C., Hegarty, L. M., Johnson, A. L. et al. (2023). CSF1R-dependent macrophages in the salivary gland are essential for epithelial regeneration after radiation-induced injury. *Sci. Immunol.* 8, eadd4374. 10.1126/sciimmunol.add4374 37922341 PMC7619197

[DEV205655C67] McNamara, N. B., Munro, D. A. D., Bestard-Cuche, N., Uyeda, A., Bogie, J. F. J., Hoffmann, A., Holloway, R. K., Molina-Gonzalez, I., Askew, K. E., Mitchell, S. et al. (2023). Microglia regulate central nervous system myelin growth and integrity. *Nature* 613, 120-129. 10.1038/s41586-022-05534-y 36517604 PMC9812791

[DEV205655C68] Miguel, V., Tituaña, J., Herrero, J. I., Herrero, L., Serra, D., Cuevas, P., Barbas, C., Puyol, D. R., Márquez-Expósito, L., Ruiz-Ortega, M. et al. (2021). Renal tubule Cpt1a overexpression protects from kidney fibrosis by restoring mitochondrial homeostasis. *J. Clin. Invest.* 131, e140695. 10.1172/JCI140695 33465052 PMC7919728

[DEV205655C69] Millard, S. M., Heng, O., Opperman, K. S., Sehgal, A., Irvine, K. M., Kaur, S., Sandrock, C. J., Wu, A. C., Magor, G. W., Batoon, L. et al. (2021). Fragmentation of tissue-resident macrophages during isolation confounds analysis of single-cell preparations from mouse hematopoietic tissues. *Cell Rep.* 37, 110058. 10.1016/j.celrep.2021.110058 34818538

[DEV205655C70] Mossadegh-Keller, N., Gentek, R., Gimenez, G., Bigot, S., Mailfert, S. and Sieweke, M. H. (2017). Developmental origin and maintenance of distinct testicular macrophage populations. *J. Exp. Med.* 214, 2829-2841. 10.1084/jem.20170829 28784628 PMC5626405

[DEV205655C71] Munro, D. A. D., Wineberg, Y., Tarnick, J., Vink, C. S., Li, Z., Pridans, C., Dzierzak, E., Kalisky, T., Hohenstein, P. and Davies, J. A. (2019). Macrophages restrict the nephrogenic field and promote endothelial connections during kidney development. *eLife* 8, e43271. 10.7554/eLife.43271 30758286 PMC6374076

[DEV205655C72] Munro, D. A. D., Bradford, B. M., Mariani, S. A., Hampton, D. W., Vink, C. S., Chandran, S., Hume, D. A., Pridans, C. and Priller, J. (2020). CNS macrophages differentially rely on an intronic Csf1r enhancer for their development. *Development* 147, dev194449. 10.1242/dev.194449 33323375 PMC7758622

[DEV205655C73] Munro, D. A. D., Bestard-Cuche, N., McQuade, A., Chagnot, A., Kiani Shabestari, S., Chadarevian, J. P., Maheshwari, U., Szymkowiak, S., Morris, K., Mohammad, M. et al. (2024). Microglia protect against age-associated brain pathologies. *Neuron* 112, 2732-2748. 10.1016/j.neuron.2024.05.018 38897208

[DEV205655C74] Nicolás-Ávila, J. A., Lechuga-Vieco, A. V., Esteban-Martínez, L., Sánchez-Díaz, M., Díaz-García, E., Santiago, D. J., Rubio-Ponce, A., Li, J. L., Balachander, A., Quintana, J. A. et al. (2020). A network of macrophages supports mitochondrial homeostasis in the heart. *Cell* 183, 94-109.e123. 10.1016/j.cell.2020.08.031 32937105

[DEV205655C75] Oosterhof, N., Chang, I. J., Karimiani, E. G., Kuil, L. E., Jensen, D. M., Daza, R., Young, E., Astle, L., van der Linde, H. C., Shivaram, G. M. et al. (2019). Homozygous mutations in CSF1R cause a pediatric-onset leukoencephalopathy and can result in congenital absence of microglia. *Am. J. Hum. Genet.* 104, 936-947. 10.1016/j.ajhg.2019.03.010 30982608 PMC6506793

[DEV205655C76] Ovchinnikov, D. A., DeBats, C. E., Sester, D. P., Sweet, M. J. and Hume, D. A. (2010). A conserved distal segment of the mouse CSF-1 receptor promoter is required for maximal expression of a reporter gene in macrophages and osteoclasts of transgenic mice. *J. Leukoc. Biol.* 87, 815-822. 10.1189/jlb.0809557 20123678

[DEV205655C77] Percin, G. I., Eitler, J., Kranz, A., Fu, J., Pollard, J. W., Naumann, R. and Waskow, C. (2018). CSF1R regulates the dendritic cell pool size in adult mice via embryo-derived tissue-resident macrophages. *Nat. Commun.* 9, 5279. 10.1038/s41467-018-07685-x 30538245 PMC6290072

[DEV205655C78] Pridans, C., Raper, A., Davis, G. M., Alves, J., Sauter, K. A., Lefevre, L., Regan, T., Meek, S., Sutherland, L., Thomson, A. J. et al. (2018). Pleiotropic impacts of macrophage and microglial deficiency on development in rats with targeted mutation of the Csf1r locus. *J. Immunol.* 201, 2683-2699. 10.4049/jimmunol.1701783 30249809 PMC6196293

[DEV205655C79] Rae, F., Woods, K., Sasmono, T., Campanale, N., Taylor, D., Ovchinnikov, D. A., Grimmond, S. M., Hume, D. A., Ricardo, S. D. and Little, M. H. (2007). Characterisation and trophic functions of murine embryonic macrophages based upon the use of a Csf1r-EGFP transgene reporter. *Dev. Biol.* 308, 232-246. 10.1016/j.ydbio.2007.05.027 17597598

[DEV205655C80] Rojo, R., Raper, A., Ozdemir, D. D., Lefevre, L., Grabert, K., Wollscheid-Lengeling, E., Bradford, B., Caruso, M., Gazova, I., Sánchez, A. et al. (2019). Deletion of a Csf1r enhancer selectively impacts CSF1R expression and development of tissue macrophage populations. *Nat. Commun.* 10, 3215. 10.1038/s41467-019-11053-8 31324781 PMC6642117

[DEV205655C81] Salei, N., Rambichler, S., Salvermoser, J., Papaioannou, N. E., Schuchert, R., Pakalniškyte, D., Li, N., Marschner, J. A., Lichtnekert, J., Stremmel, C. et al. (2020). The kidney contains ontogenetically distinct dendritic cell and macrophage subtypes throughout development that differ in their inflammatory properties. *J. Am. Soc. Nephrol.* 31, 257-278. 10.1681/ASN.2019040419 31932472 PMC7003301

[DEV205655C82] Salei, N., Ji, X., Pakalniškytė, D., Kuentzel, V., Rambichler, S., Li, N., Moser, M., Steiger, K., Buch, T., Anders, H.-J. et al. (2021). Selective depletion of a CD64-expressing phagocyte subset mediates protection against toxic kidney injury and failure. *Proc. Natl. Acad. Sci. USA* 118, e2022311118. 10.1073/pnas.2022311118 34518373 PMC8488624

[DEV205655C83] Sasmono, R. T., Oceandy, D., Pollard, J. W., Tong, W., Pavli, P., Wainwright, B. J., Ostrowski, M. C., Himes, S. R. and Hume, D. A. (2003). A macrophage colony-stimulating factor receptor-green fluorescent protein transgene is expressed throughout the mononuclear phagocyte system of the mouse. *Blood* 101, 1155-1163. 10.1182/blood-2002-02-0569 12393599

[DEV205655C84] Sauter, K. A., Bouhlel, M. A., O'Neal, J., Sester, D. P., Tagoh, H., Ingram, R. M., Pridans, C., Bonifer, C. and Hume, D. A. (2013). The function of the conserved regulatory element within the second intron of the mammalian Csf1r locus. *PLoS ONE* 8, e54935. 10.1371/journal.pone.0054935 23383005 PMC3561417

[DEV205655C85] Sehgal, A., Irvine, K. M. and Hume, D. A. (2021). Functions of macrophage colony-stimulating factor (CSF1) in development, homeostasis, and tissue repair. *Semin. Immunol.* 54, 101509. 10.1016/j.smim.2021.101509 34742624

[DEV205655C86] Sehgal, A., Carter-Cusack, D., Keshvari, S., Patkar, O., Huang, S., Summers, K. M., Hume, D. A. and Irvine, K. M. (2023). Intraperitoneal transfer of wild-type bone marrow repopulates tissue macrophages in the Csf1r knockout rat without contributing to monocytopoiesis. *Eur. J. Immunol.* 53, e2250312. 10.1002/eji.202250312 37059596

[DEV205655C87] Sester, D. P., Beasley, S. J., Sweet, M. J., Fowles, L. F., Cronau, S. L., Stacey, K. J. and Hume, D. A. (1999). Bacterial/CpG DNA down-modulates colony stimulating factor-1 receptor surface expression on murine bone marrow-derived macrophages with concomitant growth arrest and factor-independent survival. *J. Immunol.* 163, 6541-6550. 10.4049/jimmunol.163.12.6541 10586047

[DEV205655C88] Shemer, A., Grozovski, J., Tay, T. L., Tao, J., Volaski, A., Süß, P., Ardura-Fabregat, A., Gross-Vered, M., Kim, J.-S., David, E. et al. (2018). Engrafted parenchymal brain macrophages differ from microglia in transcriptome, chromatin landscape and response to challenge. *Nat. Commun.* 9, 5206. 10.1038/s41467-018-07548-5 30523248 PMC6284018

[DEV205655C89] Shibata, Y., Zsengeller, Z., Otake, K., Palaniyar, N. and Trapnell, B. C. (2001). Alveolar macrophage deficiency in osteopetrotic mice deficient in macrophage colony-stimulating factor is spontaneously corrected with age and associated with matrix metalloproteinase expression and emphysema. *Blood* 98, 2845-2852. 10.1182/blood.V98.9.2845 11675359

[DEV205655C90] Shibuya, Y., Kumar, K. K., Mader, M. M.-D., Yoo, Y., Ayala, L. A., Zhou, M., Mohr, M. A., Neumayer, G., Kumar, I., Yamamoto, R. et al. (2022). Treatment of a genetic brain disease by CNS-wide microglia replacement. *Sci Transl Med* 14, eabl9945. 10.1126/scitranslmed.abl9945 35294256 PMC9618306

[DEV205655C91] Sierro, F., Evrard, M., Rizzetto, S., Melino, M., Mitchell, A. J., Florido, M., Beattie, L., Walters, S. B., Tay, S. S., Lu, B. et al. (2017). A liver capsular network of monocyte-derived macrophages restricts hepatic dissemination of intraperitoneal bacteria by neutrophil recruitment. *Immunity* 47, 374-388.e376. 10.1016/j.immuni.2017.07.018 28813662

[DEV205655C92] Stables, J., Green, E. K., Sehgal, A., Patkar, O. L., Keshvari, S., Taylor, I., Ashcroft, M. E., Grabert, K., Wollscheid-Lengeling, E., Szymkowiak, S. et al. (2022). A kinase-dead Csf1r mutation associated with adult-onset leukoencephalopathy has a dominant inhibitory impact on CSF1R signalling. *Development* 149, dev200237. 10.1242/dev.200237 35333324 PMC9002114

[DEV205655C93] Stables, J., Pal, R., Bradford, B. M., Carter-Cusack, D., Taylor, I., Pridans, C., Khan, N., Woodruff, T. M., Irvine, K. M., Summers, K. M. et al. (2024). The effect of a dominant kinase-dead Csf1r mutation associated with adult-onset leukoencephalopathy on brain development and neuropathology. *Neurobiol. Dis.* 203, 106743. 10.1016/j.nbd.2024.106743 39581554

[DEV205655C94] Stanley, E. R. and Chitu, V. (2014). CSF-1 receptor signaling in myeloid cells. *Cold Spring Harb. Perspect. Biol.* 6, a021857. 10.1101/cshperspect.a021857 24890514 PMC4031967

[DEV205655C95] Sudo, T., Nishikawa, S., Ogawa, M., Kataoka, H., Ohno, N., Izawa, A., Hayashi, S. and Nishikawa, S. (1995). Functional hierarchy of c-kit and c-fms in intramarrow production of CFU-M. *Oncogene* 11, 2469-2476. 8545103

[DEV205655C96] Summers, K. M. and Hume, D. A. (2017). Identification of the macrophage-specific promoter signature in FANTOM5 mouse embryo developmental time course data. *J. Leukoc. Biol.* 102, 1081-1092. 10.1189/jlb.1A0417-150RR 28751473

[DEV205655C97] Summers, K. M., Bush, S. J. and Hume, D. A. (2020). Network analysis of transcriptomic diversity amongst resident tissue macrophages and dendritic cells in the mouse mononuclear phagocyte system. *PLoS Biol.* 18, e3000859. 10.1371/journal.pbio.3000859 33031383 PMC7575120

[DEV205655C98] Swirski, F. K., Nahrendorf, M., Etzrodt, M., Wildgruber, M., Cortez-Retamozo, V., Panizzi, P., Figueiredo, J. L., Kohler, R. H., Chudnovskiy, A., Waterman, P. et al. (2009). Identification of splenic reservoir monocytes and their deployment to inflammatory sites. *Science* 325, 612-616. 10.1126/science.1175202 19644120 PMC2803111

[DEV205655C99] Tan, S. Y. S. and Krasnow, M. A. (2016). Developmental origin of lung macrophage diversity. *Development* 143, 1318-1327. 10.1242/dev.129122 26952982 PMC4852511

[DEV205655C100] Taylor, I., Patkar, O. L., Liu, Y., Jacquelin, S., Ranpura, G., Carter-Cusack, D., Ewing, A., Kuurniawan, N. D., Li, M., Vasoya, D. et al. (2025). Repopulation of the brain with microglia-like cells following intraperitoneal bone marrow cell transfer in microglia-deficient mice. *bioRxiv* 2025, 2025.2001.2016.633478. 10.1101/2025.01.16.633478

[DEV205655C101] Thierry, G. R., Baudon, E. M., Bijnen, M., Bellomo, A., Lagueyrie, M., Mondor, I., Simonnet, L., Carrette, F., Fenouil, R., Keshvari, S. et al. (2024). Non-classical monocytes scavenge the growth factor CSF1 from endothelial cells in the peripheral vascular tree to ensure survival and homeostasis. *Immunity* 57, 2108-2121.e2106. 10.1016/j.immuni.2024.07.005 39089257

[DEV205655C102] van de Laar, L., Saelens, W., De Prijck, S., Martens, L., Scott, C. L., Van Isterdael, G., Hoffmann, E., Beyaert, R., Saeys, Y., Lambrecht, B. N. et al. (2016). Yolk sac macrophages, fetal liver, and adult monocytes can colonize an empty niche and develop into functional tissue-resident macrophages. *Immunity* 44, 755-768. 10.1016/j.immuni.2016.02.017 26992565

[DEV205655C103] Varol, C., Vallon-Eberhard, A., Elinav, E., Aychek, T., Shapira, Y., Luche, H., Fehling, H. J., Hardt, W. D., Shakhar, G. and Jung, S. (2009). Intestinal lamina propria dendritic cell subsets have different origin and functions. *Immunity* 31, 502-512. 10.1016/j.immuni.2009.06.025 19733097

[DEV205655C104] Weinberger, T., Denise, M., Joppich, M., Fischer, M., Garcia Rodriguez, C., Kumaraswami, K., Wimmler, V., Ablinger, S., Rauber, S., Fang, J. et al. (2024). Resident and recruited macrophages differentially contribute to cardiac healing after myocardial ischemia. *eLife* 12, RP89377. 10.7554/eLife.89377.4 38775664 PMC11111219

[DEV205655C105] Witmer-Pack, M. D., Hughes, D. A., Schuler, G., Lawson, L., McWilliam, A., Inaba, K., Steinman, R. M. and Gordon, S. (1993). Identification of macrophages and dendritic cells in the osteopetrotic (op/op) mouse. *J. Cell Sci.* 104, 1021-1029. 10.1242/jcs.104.4.1021 8314887

[DEV205655C106] Wong, N. R., Mohan, J., Kopecky, B. J., Guo, S., Du, L., Leid, J., Feng, G., Lokshina, I., Dmytrenko, O., Luehmann, H. et al. (2021). Resident cardiac macrophages mediate adaptive myocardial remodeling. *Immunity* 54, 2072-2088.e2077. 10.1016/j.immuni.2021.07.003 34320366 PMC8446343

[DEV205655C107] Xu, Y., Patterson, M. T., Dolfi, B., Zhu, A., Bertola, A., Schrank, P. R., Gallerand, A., Kennedy, A. E., Hillman, H., Dinh, L. et al. (2024). Adrenal gland macrophages regulate glucocorticoid production through Trem2 and TGF-beta. *JCI Insight* 9, e174746. 10.1172/jci.insight.174746 38869957 PMC11383592

[DEV205655C108] Yona, S., Kim, K. W., Wolf, Y., Mildner, A., Varol, D., Breker, M., Strauss-Ayali, D., Viukov, S., Guilliams, M., Misharin, A. et al. (2013). Fate mapping reveals origins and dynamics of monocytes and tissue macrophages under homeostasis. *Immunity* 38, 79-91. 10.1016/j.immuni.2012.12.001 23273845 PMC3908543

[DEV205655C109] Zhao, J., Andreev, I. and Silva, H. M. (2024). Resident tissue macrophages: key coordinators of tissue homeostasis beyond immunity. *Sci. Immunol.* 9, eadd1967. 10.1126/sciimmunol.add1967 38608039

[DEV205655C110] Zhong, L., Lu, J., Fang, J., Yao, L., Yu, W., Gui, T., Duffy, M., Holdreith, N., Bautista, C. A., Huang, X. et al. (2023). Csf1 from marrow adipogenic precursors is required for osteoclast formation and hematopoiesis in bone. *eLife* 12, e82112. 10.7554/eLife.82112 36779854 PMC10005765

