# Supplementary material for: Depletion and replacement of tissue-resident macrophages in mice with germ-line deletion of a conserved enhancer in the Csf1r locus
Source: Development. 2026 Jul 15;153(13):dev205655. doi: 10.1242/dev.205655 (PMC13405229; doi:10.1242/dev.205655)
Supplement: Supplementary information [file develop-153-205655-s1.pdf]

Figure S1

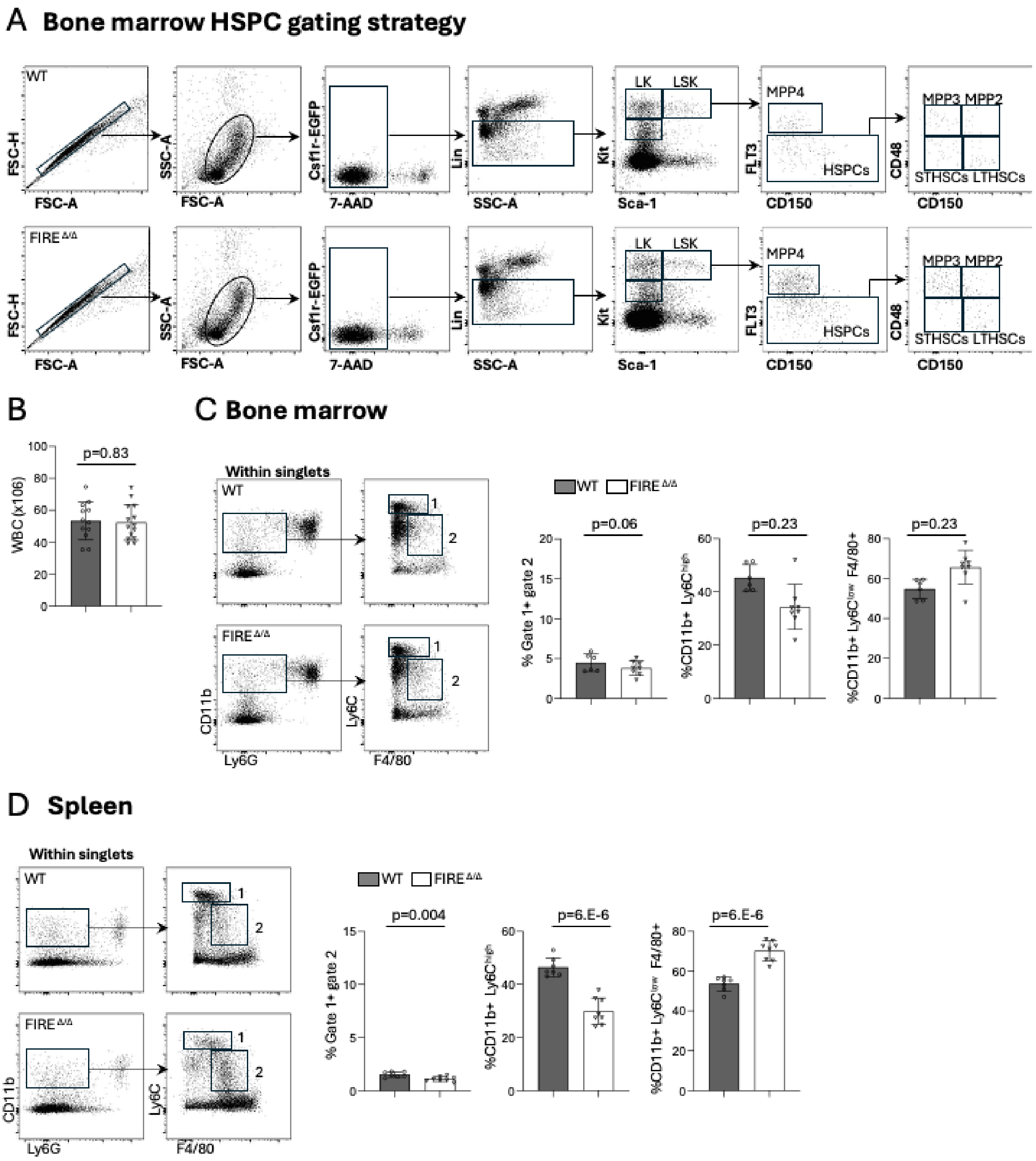

Development • Supplementary information

Fig. S1. Analysis of bone marrow progenitors and monocytes in WT and *Fireko* mice.

(A) Gating strategy for quantitation of hematopoietic stem and progenitor cell populations shown in Figure 1. Panels show representative profiles for WT and *Fireko* mice.

(B) Quantification of cellularity in bone marrow. Panel shows total white blood cells harvested from both hind legs (femur and tibia) of adult WT (N=12) and *Fireko* (N=14) mice on the C57BL/6 background.

(C) CD11b^{high}, Ly6G^{-} cells were subdivided into Ly6C^{high} (1) and Ly6C^{low}/F4/80^{+} (2) monocyte subpopulations as shown in typical flow cytometry profiles. Panels at right show quantification of cells in Gates 1+2 combined (total monocytes) and in the individual gates. Ly6C^{-}/F4/80^{+} cells are identified as resident macrophages. Data show means and standard deviation. *p* values were determined by unpaired two-tailed t-test.

(D) Quantification of myeloid subpopulations in spleen. CD11b^{high}, Ly6G^{-} cells were subdivided into Ly6C^{high} (1) and Ly6C^{low}/F4/80^{+} (2) monocyte subpopulations as shown in typical flow cytometry profiles. Panels at right show quantification of cells in Gates 1+2 combined (total monocytes) and in the individual gates. Ly6C^{-}/F4/80^{+} cells are identified as resident macrophages. Data show means and standard deviation. *p* values were determined by unpaired two-tailed t-test.

Figure S2

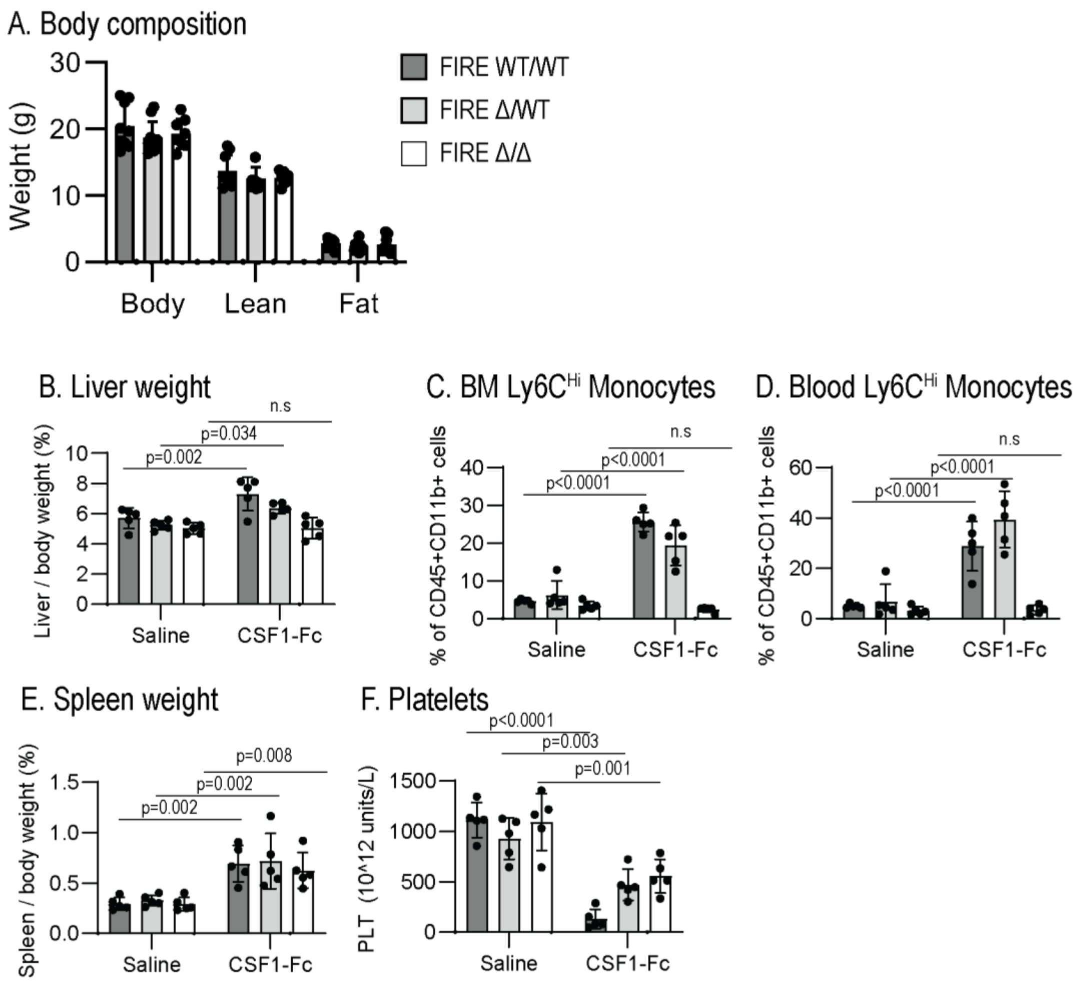

Fig. S2. CSF1 response in *Fireko* mice *in vivo*.

WT, *Csf1r*$^{\Delta FIRE/+}$ and *Fireko* mice on the C57BL/6J/CBA F2 background were injected with CSF1-Fc (1 mg/kg) or saline on each of 4 successive days and euthanised on day 5. (A) Prior to treatment, body composition was analysed using a Bruker Minispec. Each point is an individual animal. (B-F) C57/CBA mice were injected with CSF1-Fc (1 mg/kg) or saline on each of 4 successive days and euthanised on day 5. n = 5 per genotype (B) Liver/body weight ratio. Bone marrow (C) and peripheral blood (D) Ly6C^{high} monocytes were gated as CD45^{+}/Ly6G^{-}/CD11b^{+}/Ly6C^{high}. (E) Spleen/body weight ratio.(F) Platelet counts were determined using Mindray analyser. Data are mean ± SD; two-way ANOVA with Sidak's multiple comparison test for Saline vs CSF1-Fc, n.s. not significant.

Development • Supplementary information

Figure S3

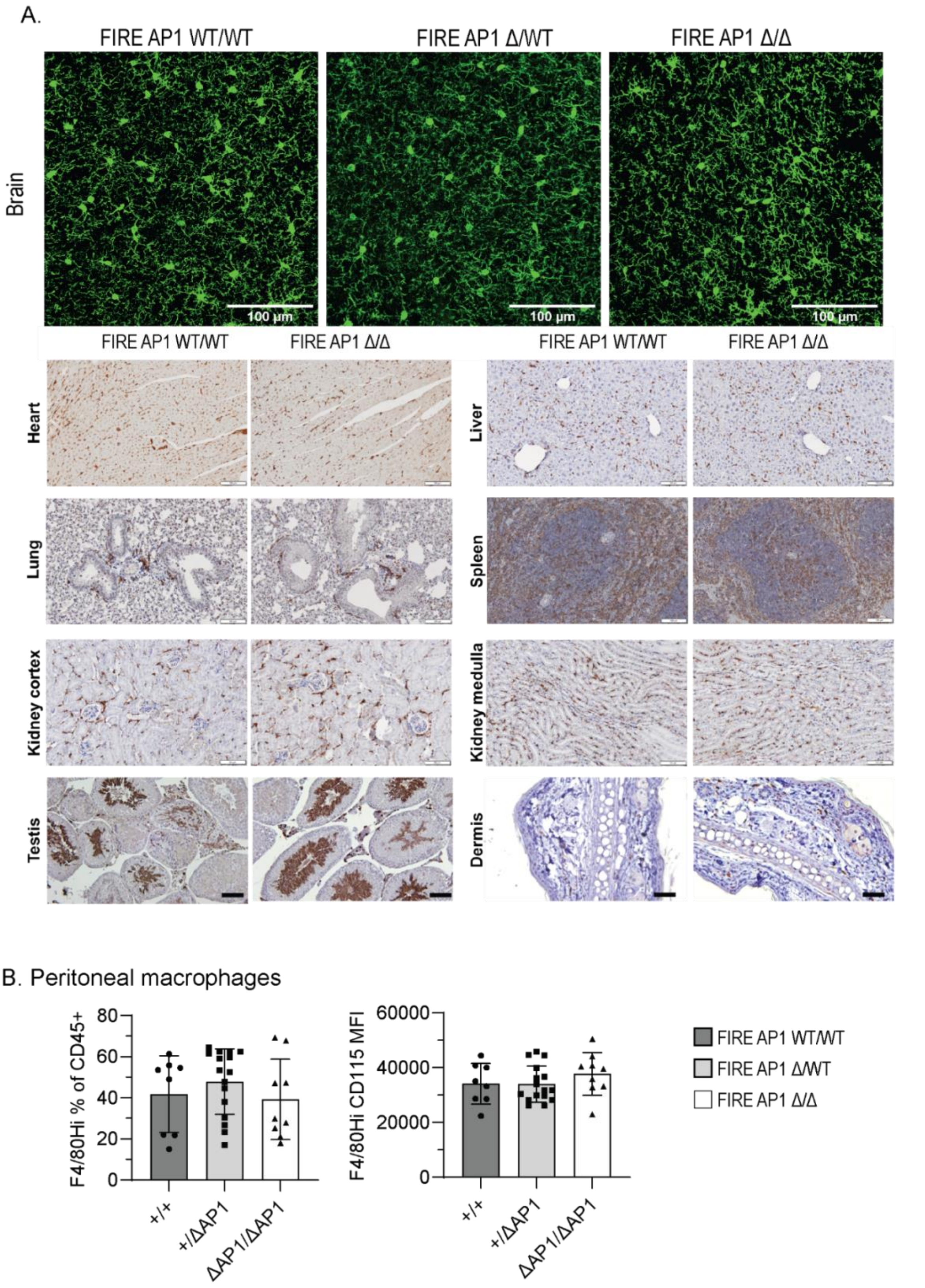

Fig. S3. *Csf1r* FIRE-AP1 mutation does not impact brain or peripheral macrophage populations.

Development • Supplementary information

(A) Brain cortex, heart, liver, lung, spleen, kidney, testis and dermis sections from 9 week old *Csf1r*$^{+/+}$, *Csf1r*$^{+/\Delta AP1}$ or *Csf1r*$^{\Delta AP1/\Delta AP1}$ mice were stained for IBA1 (representative images). Scale bar in peripheral tissues = 50 µm. (B) Peritoneal cells from 3 week old *Csf1r*$^{+/+}$, *Csf1r*$^{+/\Delta AP1}$ or *Csf1r*$^{\Delta AP1/\Delta AP1}$ mice were analysed by flow cytometry. Resident peritoneal macrophages were gated as CD45^{+}/Cd11b^{high}/F4/80^{high}. MFI: median fluorescence intensity.

Development • Supplementary information

Figure S4

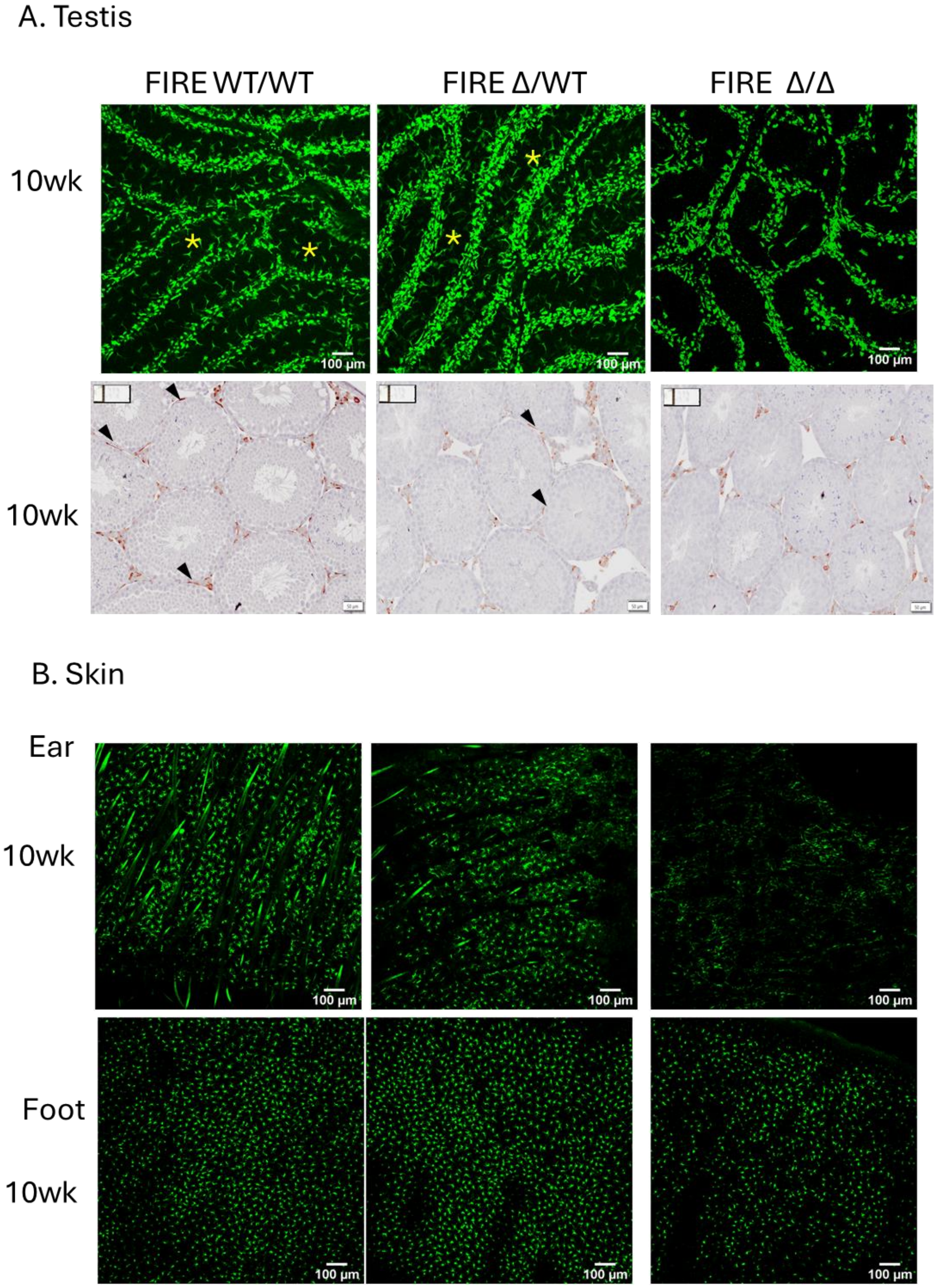
A. Testis
FIRE WT/WT
FIRE Δ/WT
FIRE Δ/Δ
10wk
100 μm
100 μm
100 μm
10wk
B. Skin
Ear
10wk
100 μm
100 μm
100 μm
Foot
10wk
100 μm
100 μm
100 μm

Figure S4

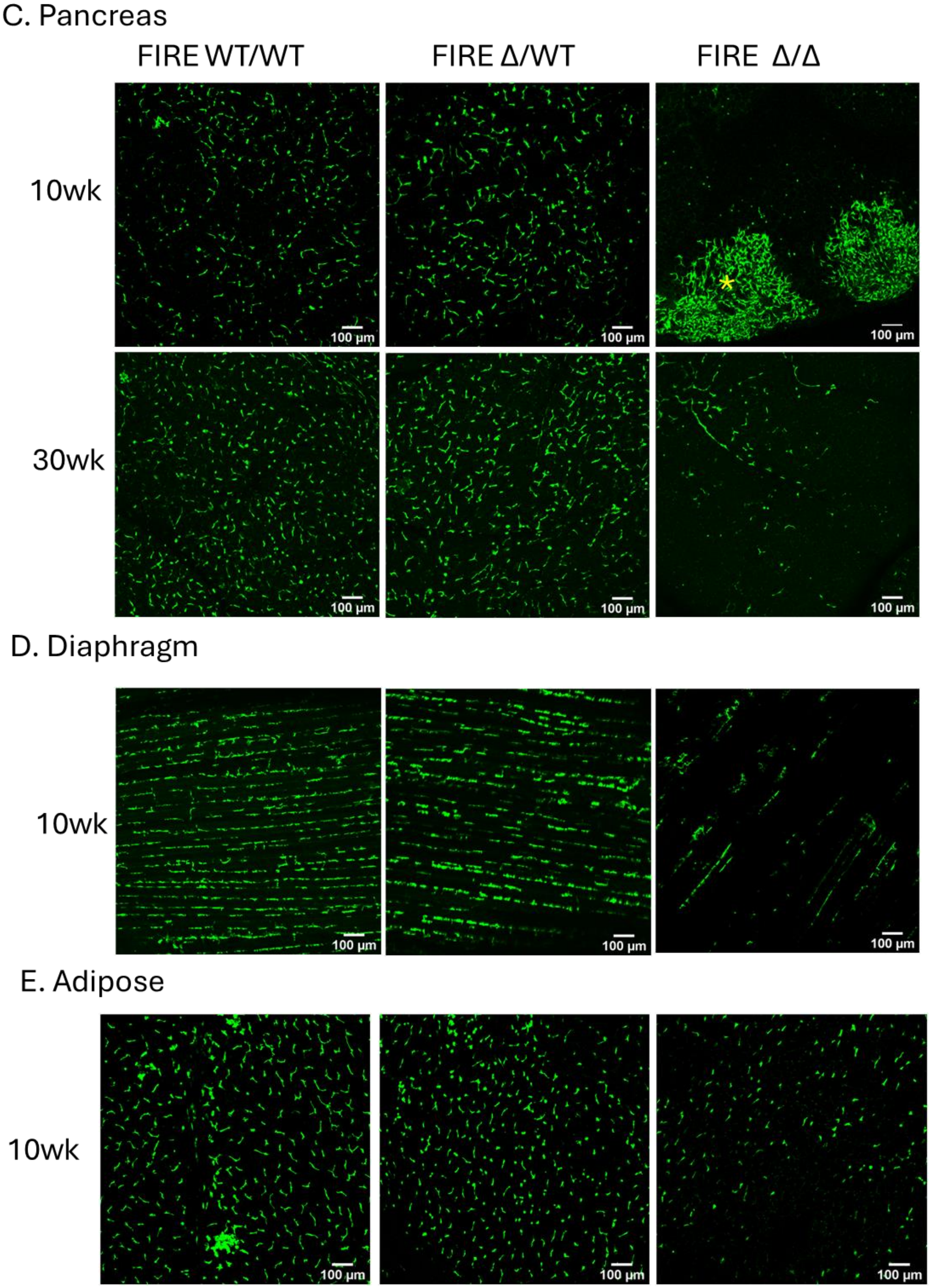
C. Pancreas
FIRE WT/WT
FIRE Δ/WT
FIRE Δ/Δ
10wk
30wk
100 µm
D. Diaphragm
10wk
E. Adipose
10wk

Fig. S4. Detection of tissue resident macrophages in *Fireko* mice.

Representative whole mount images of *Csf1r*-EGFP in indicated tissues from adult male WT, *Csf1r*$^{\Delta FIRE/+}$ and *Fireko* mice. In the testis, note the GFP^{+} macrophages spread on the surface of the tubules (asterisks) in WT and D/+ that are not detected in *Fireko* (D/D) whereas the interstitial macrophages associated with the interstitium are retained. The lower panel shows localisation of F4/80. The image of the *Fireko* pancreas at 10 weeks includes two lymphoid aggregates (asterisk) where EGFP^{+} cells are retained, whereas no signal is detected in the acinar tissue.

Development • Supplementary information

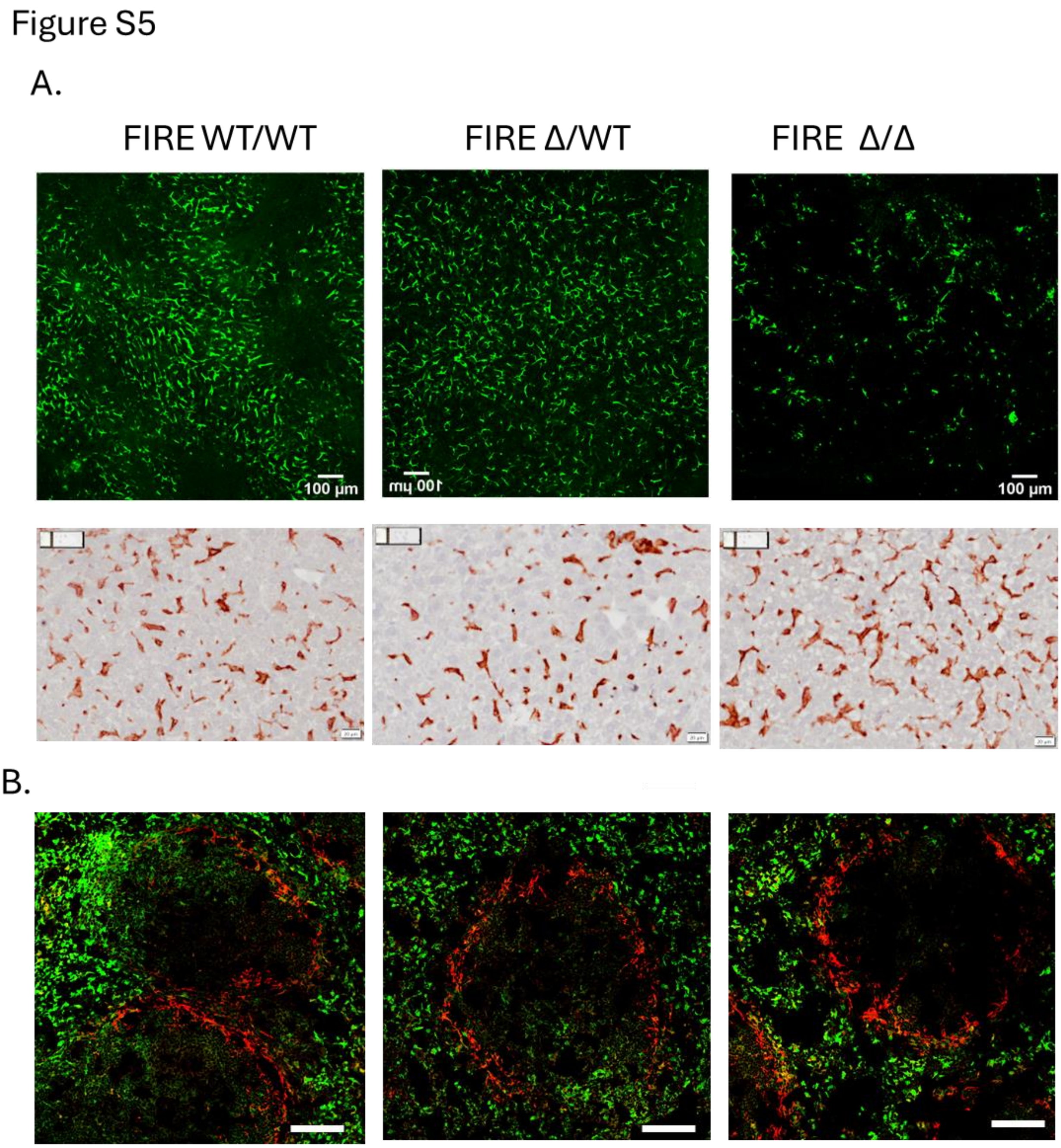

Fig. S5. Detection of tissue resident macrophages in *Fireko* mice.

(A) Representative whole mount images of *Csf1r*-EGFP and F4/80 staining of liver from adult male WT, *Csf1r*$^{\Delta FIRE/+}$ and *Fireko* mice. Note the depletion of EGFP^{+} capsular macrophages in *Fireko* (D/D) liver whereas F4/80^{+} Kupffer cells are unaffected. B) Representative images of *Csf1r*-EGFP and CD169 (red) immunofluorescence localisation in spleen from adult male WT, *Csf1r*$^{\Delta FIRE/+}$ and *Fireko* mice. (C) F4/80 staining in adrenal gland sections from adult WT, *Csf1r*$^{\Delta FIRE/+}$ and *Fireko* mice.

Development • Supplementary information

Figure S6

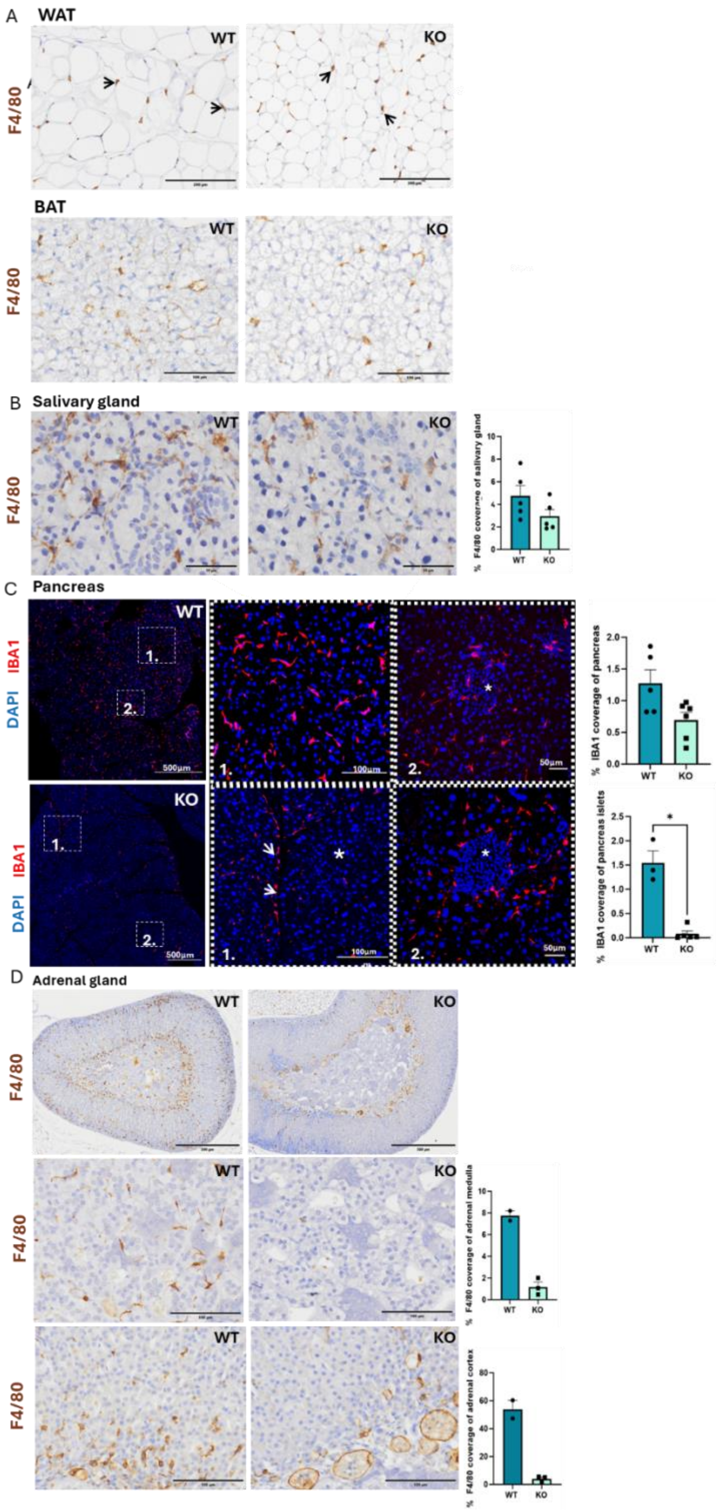
A WAT
F4/80
WT
KO
BAT
B Salivary gland
% F4/80 coverage of salivary gland
C Pancreas
DAPI IBA1
500µm
100µm
50µm
% IBA1 coverage of pancreas
% IBA1 coverage of pancreas islets
D Adrenal gland
% F4/80 coverage of adrenal medulla
% F4/80 coverage of adrenal cortex

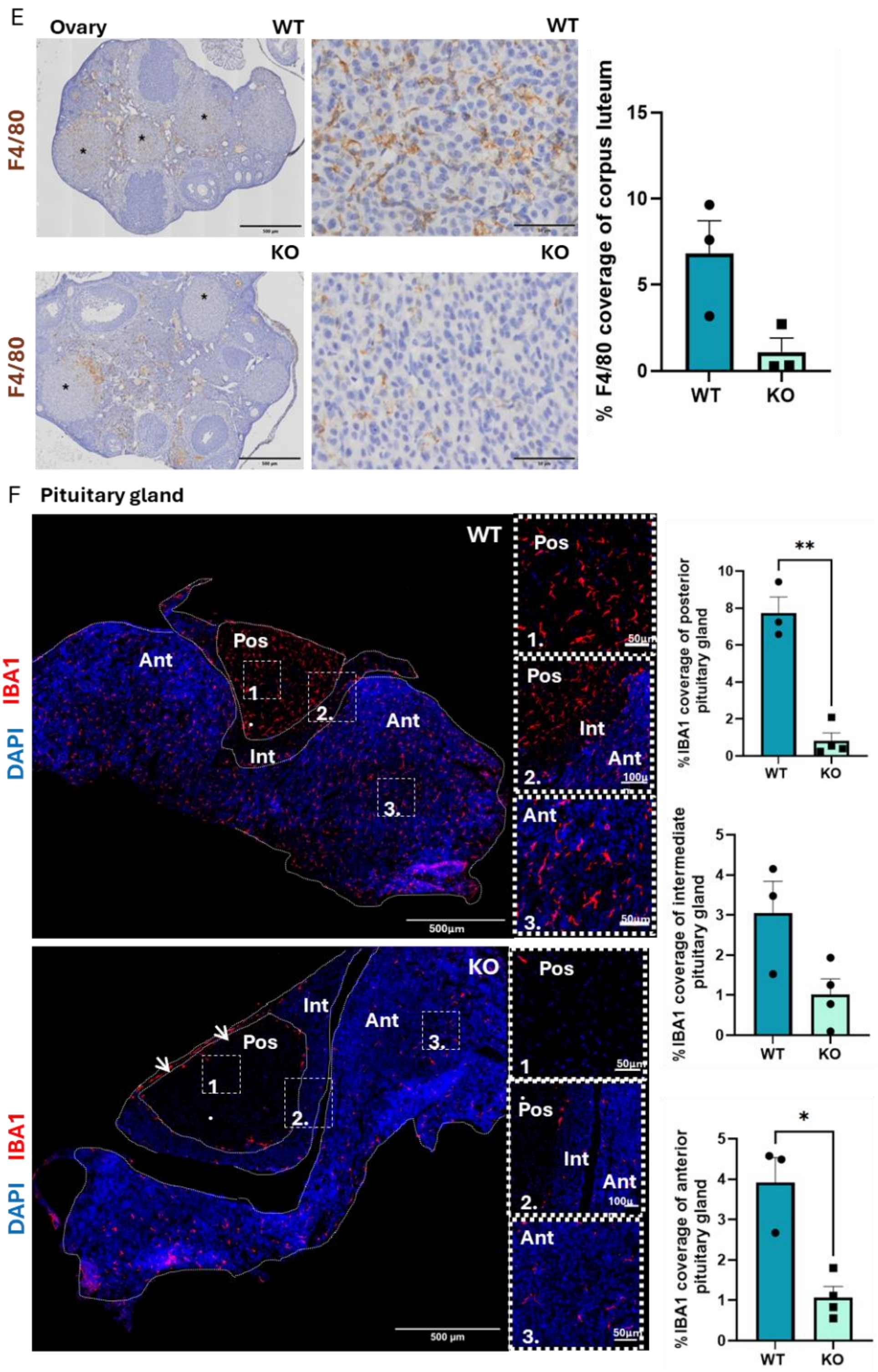

Fig. S6. Detection of tissue resident macrophages in *Fireko* mice.

(A) Representative images of F4/80 staining of white adipose tissue and brown adipose tissue from adult WT and *Fireko* mice. Arrows highlight individual F4/80^{+} interstitial macrophages. No quantitative diferennce was detected based upon genotype.

(B) Representative images of F4/80 staining of salivary gland from adult WT and *Fireko* mice and quantitation. N=5 per group.

Development • Supplementary information

(C) Localisation of IBA1$^+$ macrophages in the pancreas of WT and *Fireko* mice. Panels show typical higher magnification images of exocrine and endocrine (islet) tissue (asterisks). % stained area was quantified; n=5 per group. Note that although there was not a significant difference between WT and *Fireko* in exocrine tissue, in the *Fireko* pancreas IBA1$^+$ cells appeared restricted to interlobular connective tissue (arrows).

(D) Representative images of F4/80 staining of adrenal gland tissue from adult WT and *Fireko* mice. Higher magnifications show the medulla and cortex. % stained area was quantified; n=3 per group.

(E). Representative images of F4/80 staining of ovaries from adult WT and *Fireko* mice. Higher magnifications show corpus luteum (asterisks). % stained area in corpora lutea was quantified; n=3 per group.

(F) Localisation of IBA1$^+$ macrophages in the pituitary of WT and *Fireko* mice. Low and high magnification images show the separate posterior, intermediate and anterior pituitary lobes as indicated. % stained area in each area was quantified; Each point is an individual mouse. Note that posterior pituitary parenchyma lacks detectable IBA1$^+$ cells; IBA1$^+$ cells are retained only on the surface (arrows).

Development • Supplementary information

Figure S7

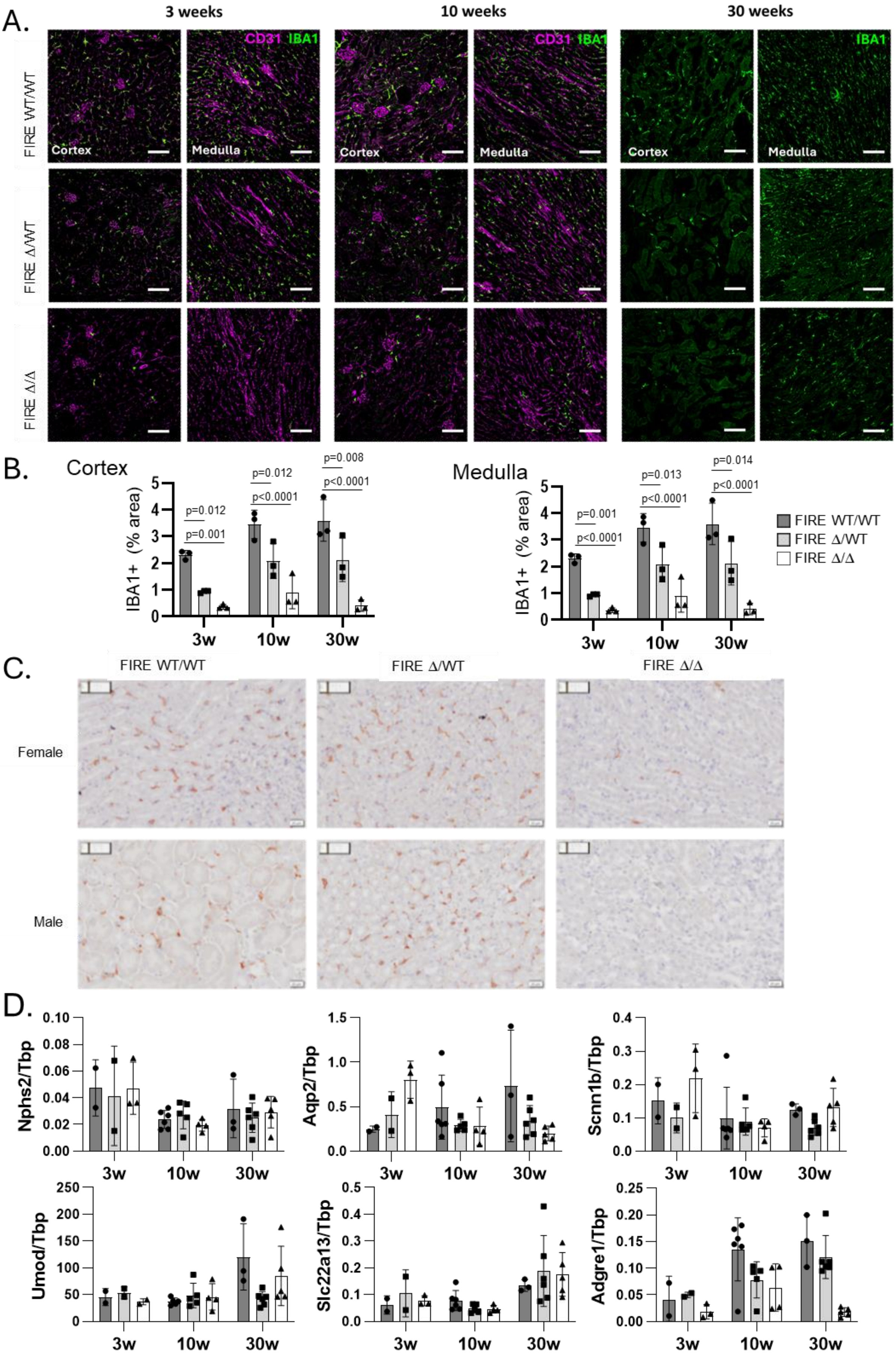

Development • Supplementary information

E.

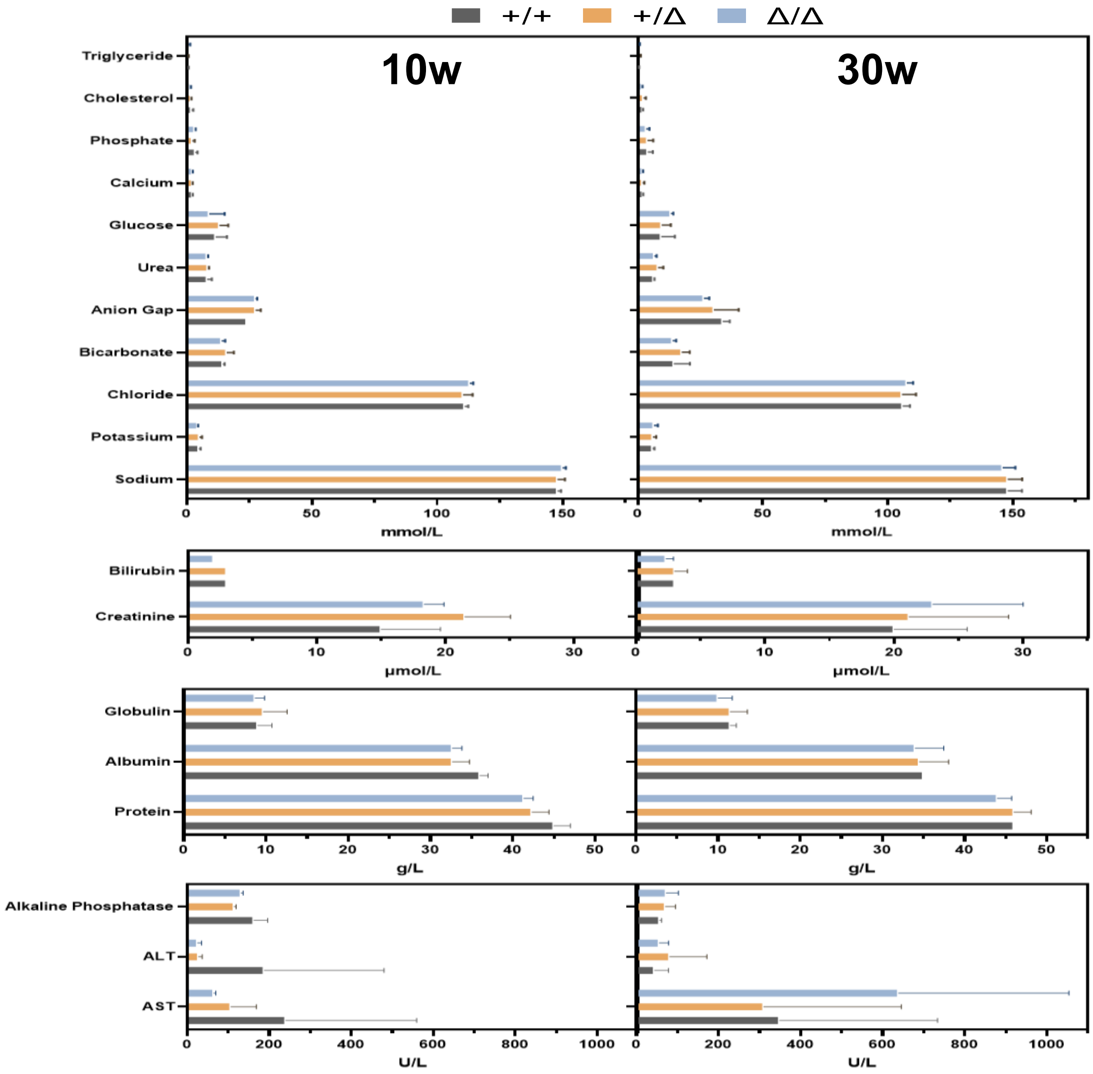

Fig. S7.

(A) Representative images of sections from WT, *Csf1r*$^{\Delta FIRE/+}$ and *Fireko* mice at 3, 10 or 30 wks of age stained for IBA1 and CD31. Scale bar 100µm.

(B) IBA1^{+} area in the cortex and medulla was quantified using image analysis. Data show mean and standard deviation, each datapoint is an individual mouse. 2 way Anova with Tukey's multiple comparison test.

(C) Representative images of kidney sections from WT, *Csf1r*$^{\Delta FIRE/+}$ and *Fireko* mice at 10 wks of age were stained for F4/80.

(D) Kidney mRNA expression of *Nphs2, Aqp2, Scnn1b, Umod, Slc22a13* and *Adgre1* was determined by qRT-PCR at 3, 10 and 30 wks of age as indicated

(E) Serum electrolytes, proteins, markers of liver (alanine aminotransferase (ALT), aspartate aminotransferase (AST)) and kidney (alkaline phosphatase, creatinine, urea) were measured in serum from WT, *Csf1r*$^{\Delta FIRE/+}$ and *Fireko* mice at 10 and 30 weeks as indicated. N=3/group.

Development • Supplementary information

Figure S8

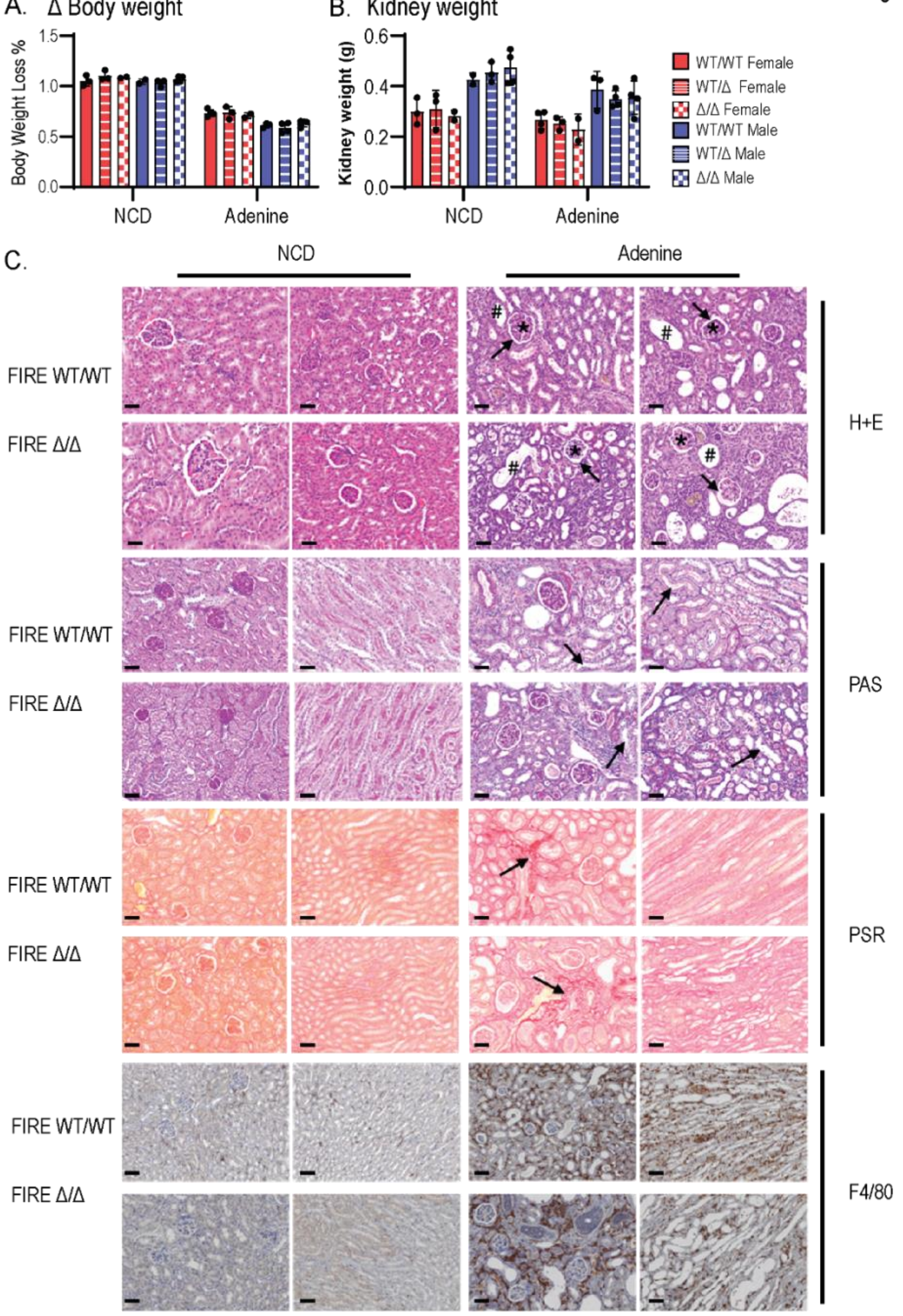

D.

Parameters	Males				Females			
	Chow		Adenine		Chow		Adenine	
	WT	ΔFIRE/ΔFIRE	WT	ΔFIRE/ΔFIRE	WT	ΔFIRE/ΔFIRE	WT	ΔFIRE/ΔFIRE
% Total renal affected area	0.0	0.0	81.7 ± 6.1	81.2 ± 12.5	0.0	0.0	79.4 ± 15.7	73.8 ± 6.3
% Tubular atrophy	0.0	0.0	35.8 ± 10.2	40.6 ± 5.0	0.0	0.0	55.6 ± 21.6	57.5 ± 9.6
% Tubules with Casts and cell debris	0.0	0.0	23.3 ± 12.1	25.0 ± 13.9	0.0	0.0	8.9 ± 5.6	7.5 ± 2.9
Apoptosis tubular epithelium	0.0	0.0	1.3 ± 0.5	1.2 ± 0.5	0.0	0.0	1.6 ± 0.5	1.2 ± 0.5
Glomerular sclerosis	0.0	0.0	0.0 ± 0.0	0.0 ± 0.0	0.0	0.0	0.0 ± 0.0	0.2 ± 0.5
Thickening bowman's capsule	0.0	0.0	21.7 ± 13.3	18.8 ± 11.3	0.0	0.0	0.0 ± 0.0	0.2 ± 0.5

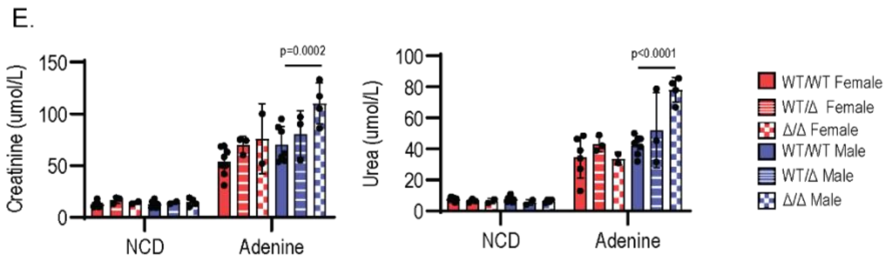

Fig. S8. Adenine-induced chronic kidney disease in *Fireko* mice. Male and female WT, *Csf1r*$^{\Delta FIRE/+}$ and *Fireko* (FIRE Δ/Δ) mice were fed normal chow (NCD) or a diet containing 0.2% adenine for 3 weeks. (A) Body weight loss during the treatment period. (B) Kidney weight at sacrifice. (C) Representative sections from male mice (N=5/group) from mice of indicated genotype and diet stained for H&E, periodic acid Schiff (PAS) stain, picrosirius red (PSR) or immunostained for F4/80. (D) Kidney pathology was scored from the H&E sections by a veterinary pathologist (RA) who was blinded to the genotype as described in Materials and Methods. N=5/group (E) Creatinine and urea was measured in serum taken at time of sacrifice on NCD or adenine diet as indicated. Each point is an individual animal. N=3-5/group. Data show mean and standard deviation. P values based on 2 way ANOVA with Tukey's multiple comparisons test.

Development • Supplementary information

Figure S9

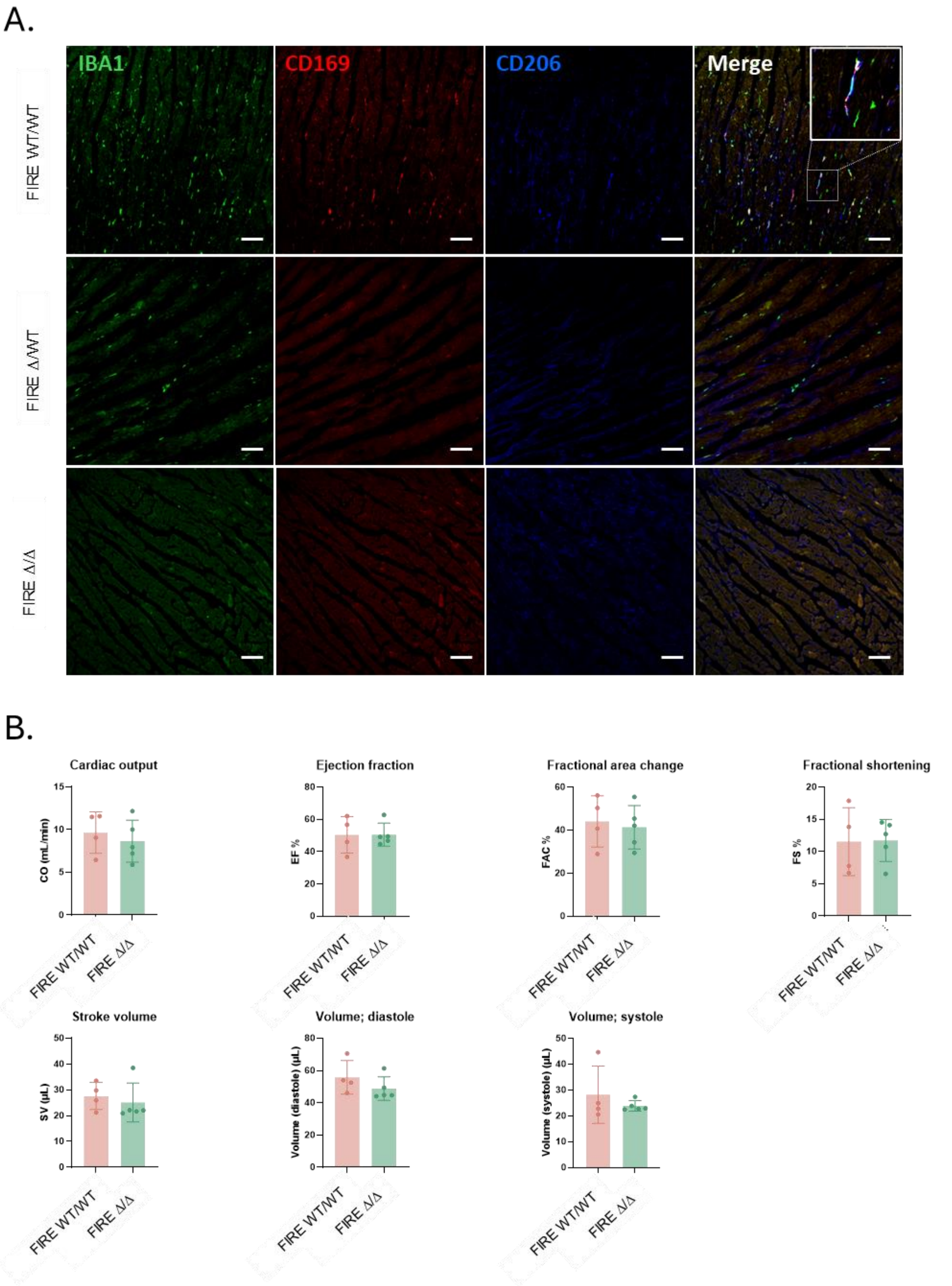

Fig. S9. Cardiac macrophage deficiency in *Fireko* mice does not impact steady state heart function

(A) Representative images of heart sections from WT, *Csf1r*$^{\Delta FIRE/+}$ and *Fireko* mice at 30 wks of age stained for IBA1, CD169 and CD206 as indicated. (B) Cardiac function was assisted in a cohort of adult male (13-19 wks) WT and *Fireko* mice. Each point is an individual animal.

Development • Supplementary information

Figure S10

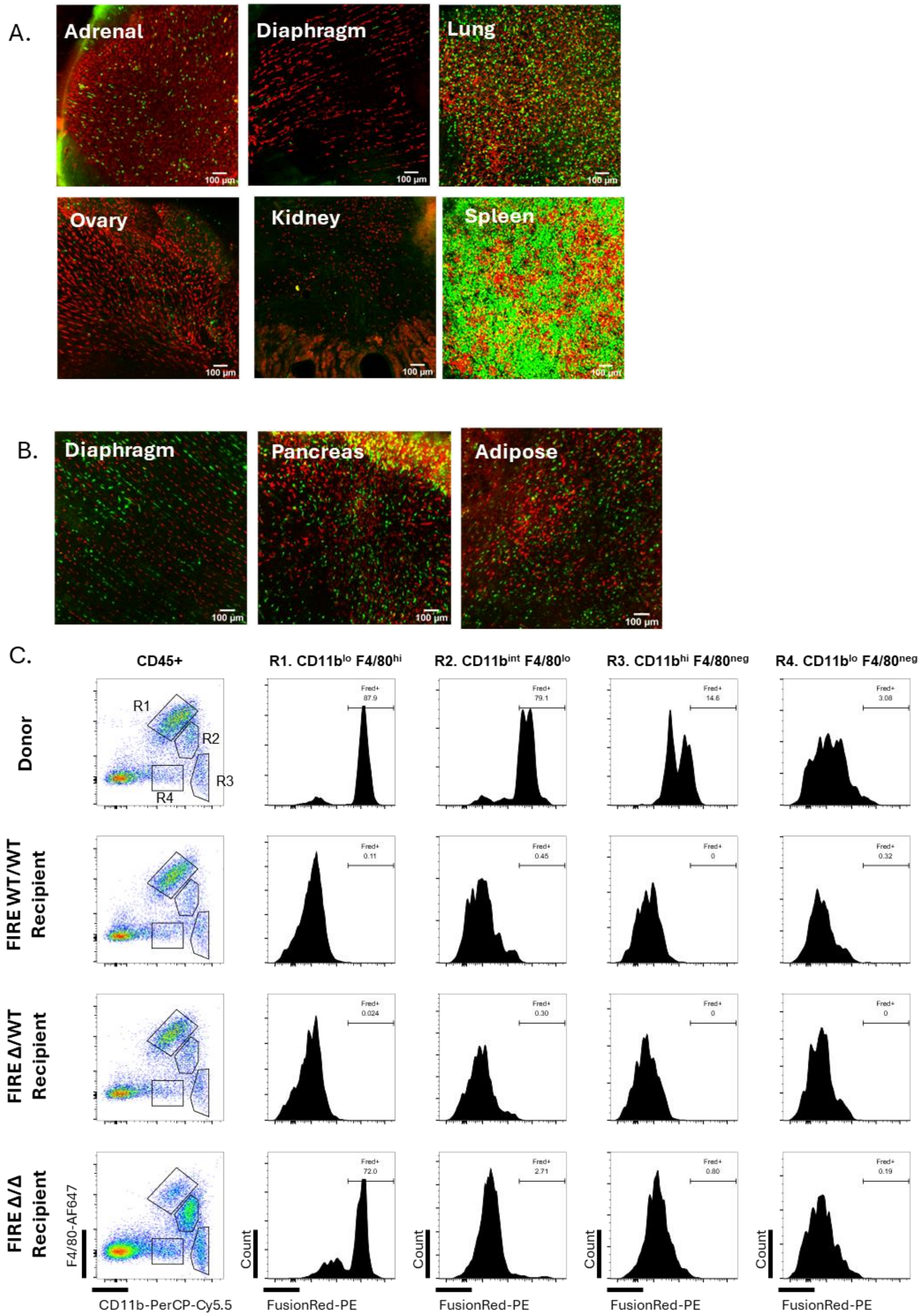

Development • Supplementary information

Fig. S10. Repopulation of tissue macrophages in *Fireko* mice.

Csf1r-EGFP^{+} WT, *Csf1r*$^{\Delta FIRE/+}$ and *Fireko* mice were injected i.p. at weaning with WT *Csf1r*-FRed donor cells and chimerism was assessed by whole mount imaging 12 wks post transfer. (A) Representative whole mount images of the tissues indicated in *Fireko* recipients.

(B) Whole mount images of tissues of *Csf1r*$^{\Delta FIRE/+}$ recipients in which donor chimerism was detected.

(C) Representative flow cytometry profiles of kidney macrophage populations in *Csf1r*-FRed donors and WT, *Csf1r*$^{\Delta FIRE/+}$ and *Fireko* bone marrow recipients.

Development • Supplementary information

Table S1. Sources of antibodies for Flow Cyometry

Flow antibody list			
**Antibody**	**Flurorochrome**	**Reference**	**Provider**
Anti-mouse CD11b	Percp-Cy5.5	Cat#101228	Biolegend
Anti-mouse Ly6G	BV785	Cat#127645	Biolegend
Anti-mouse Ly6C	BV421	Cat#128008	Biolegend
Anti mouse NK1.1	AF700	Cat#560515	BD Biosciences
Anti mouse CD135	APC	Cat#135310	Biolegend
Anti mouse F4/80	BUV395	Cat#565614	BD Biosciences
Anti mouse F4/80	A647	Cat#123122	Biolegend
Anti mouse CD115	BUV605	Cat#135517	Biolegend
Anti mouse c-kit	PECy7	Cat#135112	Biolegend
Anti-mouse MHC2	FITC	Cat#107605	Biolegend
Anti-mouse CD11c	APC-Cy7	Cat# 117323	Biolegend
Anti-Mouse CD319	BV711	Cat#747994	BD Biosciences
Anti mouse CD177	AF647	Cat#566599	BD Biosciences
Anti mouse Ly-6A/E	BV650	Cat#108143	Biolegend
Anti mouse CD150	BV711	Cat#115941	Biolegend
Anti mouse CD48	BV421	Cat#1103418	Biolegend
Anti mouse CD16/32	APC	Cat#101326	Biolegend
Anti mouse CD34	BV421	Cat#343610	Biolegend
Anti mouse CD71	PE-Cy7	Cat# 113812	Biolegend
Anti mouse TER119	APC	Cat#116212	Biolegend
Anti mouse KI67	A647	Cat#652406	BD Biosciences
Hoechst 33342	BV496	Cat# 561908	BD Pharmingen
Anti mouse B220	APC-Cy7	Cat# 103224	Biolegend
Anti mouse CD19	PE-Dazzle	Cat# 115554	Biolegend
Anti mouse CD3	APC	Cat#100236	Biolegend
Anti mouse CD4	Percp-Cy5.5	Cat# 100540	Biolegend
Anti mouse CD8	BV785	Cat# 100750	Biolegend
Anti mouse CD44	BV421	Cat#103040⍰	Biolegend
Anti mouse CD62L	PE-Cy7	Cat# 104418⍰	Biolegend
Biotin Anti-mouse Ly-6G/Ly-6C	N/A	Cat# 108404	Biolegend
Biotin Anti-mouse/human CD45R	N/A	Cat# 103204	Biolegend
Biotin Anti-mouse TER-119	N/A	Cat# 116204	Biolegend
Biotin Anti-mouse CD3 Antibody	N/A	Cat# 100244	Biolegend
Biotin Anti-mouse CD5 Antibody	N/A	Cat# 100604	Biolegend
Streptavidin	APC-Cy7	Cat# 405208	Biolegend
FIX & PERM Cell permeabilization kit		GAS003	Thermofisher

Table S2. Primer sequences

**Gene**	**Forward primer**	**Reverse primer**
Adgre1	CTGTCTGCTCAACCGTCAGGTA	AGAAGTCTGGGAATGGGAGCTAA
Hprt	GCAGTACAGCCCCAAAATGG	AACAAAGTCTGGCCTGTATCCAA
Tbp	CTCAGTTACAGGTGGCAGCA	ACCAACAATCACCAACAGCA
Gapdh	GACATGCCGCCTGGAGAAAC	AGCCCAGGATGCCCTTTAGT
Csf1r	CTGGGAGATCTTCTCGCTTGGT	CTCCAGGTCCCAGCAGGACT
Nphs2	AGGAAGAGCATTGCCCAAGA	CTGGCAGCCTCACATCCTTAAT
Aqp2	GCAAGTTTGATGATCACTGGGTCT	TCGAGGGGAACAGCAGGTAGT
Scnn1b	CGAATATCACCCTGAGCAGGAA	ATTGTTGGCTGGAGATTCCTCA
Umod	GGTCCCATAACACGACAAGGTG	GGCTGAGGGAAACAACAGCA
Slc22a13	GGCCTCAGTCCCTAAAGAAAGAGTT	CAACAAGGAGACCAGCCATGA
