# Supplementary material for: Depletion and replacement of tissue-resident macrophages in mice with germ-line deletion of a conserved enhancer in the Csf1r locus
Source: Development. 2026 Jul 15;153(13):dev205655. doi: 10.1242/dev.205655 (PMC13405229; doi:10.1242/dev.205655)
Supplement: Supplementary file 1 [file develop-153-205655_review_history.pdf]

Development

Depletion and replacement of tissue-resident macrophages in mice with germ-line deletion of a conserved enhancer in the *Csf1r* locus

Yajun Liu, Sebastien Jacquelin, Isis Taylor, Emma K. Green, Omkar L. Patkar, Sahar Keshvari, Ginell Ranpura, Conan J. O. O'Brien, Eline Jessen, Emma Maxwell, Rachel Allavena, Alexandre Gallerand, Stoyan Ivanov, Antony Adamson, Neil E. Humphreys, Kim M. Summers, Katharine M. Irvine and David A. Hume
DOI: 10.1242/dev.205655

Editor: James Briscoe

Review timeline

Original submission:	24 September 2025
Editorial decision:	14 November 2025
Resubmission:	10 March 2026
Editorial decision:	7 April 2026
First revision received:	13 May 2026
Editorial decision:	2 June 2026
Second revision received:	14 June 2026
Accepted:	15 June 2026

Original submission

First decision letter

MS ID#: dev.205266

MS Title: The impact of deletion of a conserved enhancer in the mouse Csf1r locus highlights functional redundancy in the mononuclear phagocyte system.

Authors: Yajun Liu; Sebastien Jacquelin; Isis Taylor; Omkar L. Patkar; Sahar Keshvari; Ginell Ranpura; Conan J. O. O'Brien; Emma K. Green; Eline Jessen; Emma Maxwell; Rachel Allavena; Alexandre Gallerand; Stoyan Ivanov; Antony Adamson; Neil E Humphreys; Kim M. Summers; Katharine M. Irvine; David A Hume

Article Type: Research Article

Dear Dr Hume,

I have now received all the referees reports on the above manuscript, and have reached a decision. The referees' comments are appended below.

The referees acknowledge that your study addresses a scientifically relevant question regarding FIRE enhancer regulation of Csf1r and macrophage development. However, they raise fundamental concerns that preclude publication at this time. The referees' opinion is that conclusions about monocytopoiesis, macrophage tissue distribution, and repopulation completeness are not adequately supported by the data. Specifically, the claims of macrophage absence rely on reporter imaging without orthogonal validation, risking false negatives. The conclusion of "complete" repopulation appears to contradict your own data showing incomplete replacement in multiple subsets and the proposed novel CSF1R-dependent progenitor population lacks sufficient experimental support. Given the nature of these concerns, and the length of time that it would

© 2026. Published by The Company of Biologists under the terms of the Creative Commons Attribution License (https://creativecommons.org/licenses/by/4.0/).

take to address these issues, I am afraid I have little choice other than to reject the paper at this stage.

However, I do recognise the potential importance of this work. I would therefore be prepared to consider as a new submission an extension of this study that contains new experiments, data and discussions and that address fully the major concerns of the referees. The work required goes beyond a standard revision of the paper. Please bear in mind that the referees (who may be different from the present reviewers) will assess the novelty of your work in the context of all previous publications, including those published between now and the time of resubmission.

Comments from the Reviewers:

Reviewer 1: SUMMARY OF THE ADVANCE MADE IN THIS PAPER AND ITS POTENTIAL SIGNIFICANCE TO THE FIELD

In this report, the authors employed the Fireko mouse model to address the effect of the Csf1r deltaFIRE mutation on monocytopoiesis, CSF1 responsiveness and regulation of Csf1r expression and signaling. They also assessed macrophage abundance across organs, function of renal and heart macrophages, and repopulation following adoptive transfer of wild-type cells in Fireko mice. The manuscript is well written, the experiments are well executed, the long discussion reflects the different aspects evaluated in this report. Few suggestions for improvement can be found below.

SUGGESTIONS TO AUTHORS

1. The study infers preserved monocytopoiesis based on steady-state monocyte counts without directly assessing production or egress rates from the bone marrow. However, the population snapshots shown in Figure 1 (progenitor frequencies and monocyte counts) cannot distinguish normal production from compensatory mechanisms such as prolonged monocyte lifespan, altered egress, or extramedullary production. Without kinetic measurements, the conclusion that monocytopoiesis proceeds independently of CSF1R in progenitors remains speculative. This limitation impacts the interpretation of Figure 1 datasets.
2. Several organ-wide "absence" claims rely primarily on reporter imaging without comprehensive, reporter-independent validation or quantitative cell counts. Dependence on Csf1r-EGFP reporter expression risks missing macrophages with reduced CSF1R or altered F4/80 expression, leading to false negatives, particularly in tissues such as pancreas, adipose, and skeletal muscle. As established in lineage tracing studies, reporter-based methods alone can underrepresent cell populations due to technical limitations. Orthogonal validation (e.g., alternative markers, immunostaining, or flow cytometry) is essential for robust conclusions. Without such controls, the assertion that these tissues lack macrophages may overstate depletion and weakens the inference of tissue-selective loss based solely on reporter signal. It is also important to note that Csf1r-EGFP reporter mice depend on FIRE enhancer activity, further influencing expression patterns.
3. The breadth and completeness of macrophage repopulation are predicted from qualitative imaging of selected tissues without systematic, quantitative coverage across organs and macrophage subsets. Some subsets remain host-derived (e.g., alveolar macrophages and Kupffer cells), and only the F4/80high population was replaced in kidney. Therefore, the claim of "complete" repopulation across all organ niches is not fully supported by the data. This concern is reinforced by known heterogeneity of tissue-resident macrophages, including functionally distinct subsets within individual organs, and by evidence that developmental origin influences repopulation dynamics. Furthermore, donor-derived brain macrophages exhibit altered marker expression and morphology, suggesting phenotypic differences that complicate the notion of equivalent replacement. Without organ-wide quantitative counts benchmarked to wild-type densities for each subset, generalizations such as "every organ" and "complete" repopulation are broader than the presented evidence supports.

Minor Comments
Page 6, line 6: Figure 1 does not show data for CMP and GMP, though these are mentioned in the text.
Page 7, line 1: Should state 37 °C instead of 4 °C.
Page 8, line 13: According to Figure 2, there is a decrease in MHCII high cells (not an increase) and an increase in MHCII low cells (not a decrease).

© 2026. Published by The Company of Biologists under the terms of the Creative Commons Attribution License (https://creativecommons.org/licenses/by/4.0/).

Page 8, line 17 and Figure 2 legend: E. coli should be italicized.
Page 20, line 11: Missing period at the end of the sentence.

Comments on Figures
Panel annotations vary (sometimes "A." vs "A" vs bold "A"); please maintain a consistent style.
Bar graphs: group distinctions use both shades of gray and symbols inconsistently; choose one system for clarity.
Figure 2 legend: Refers to "CSF1R MFI," while the figure shows "CD115 MFI"; harmonize terminology.
Figure 4C: Gene names should be italicized.
Figure 4B: Missing legend.
Figure 5A: Missing scale bar.
Figure 5B: Missing legend.
Figure 6: Scale bar length not specified.
Figure S4B: Scale bar length not specified.

Reviewer 2: This study investigates the role of the conserved FIRE enhancer within the Csf1r locus, which regulates macrophage development and CSF1R signalling. Mice lacking FIRE exhibit widespread loss of tissue resident macrophages, including microglia and macrophages in the heart, kidney, and other organs, though monocyte numbers remain intact. Using a new congenic Fireko line with a Csf1r-EGFP reporter, the authors show that FIRE is dispensable for Csf1r expression in GM-CSF-responsive cells and inflammatory macrophages but essential for CSF1-driven transcription. Mutation of the conserved AP1 site within FIRE further impaired CSF1 responsiveness, underlining that CSF1 signalling depends on the rate of CSF1R synthesis. Finally, macrophage-deficient tissues in Fireko mice could be repopulated by adoptive transfer of wild-type bone marrow cells without conditioning.

The study is interesting in principle but is superficial, descriptive and correlative in nature. There is a lack of real connection between the experiments done, from BM to peritoneum to kidney, with a last part dealing with repopulation with BM transfer. There is no real clear messages and some conclusions are quite strong with no real data supporting them. In this regard, the last part claiming the presence of a CSF1R-dependent macrophage progenitor population in the BM with high proliferative potential that gives rise directly to resident tissue macrophages is weak and require further work to be supported. A lot of cofounding factors such as the lack of comparators (monocytes?), the site of injection as well as the Fireko background could be a play.

The authors mentioned "F4/80high bone marrow resident macrophages associated with hematopoietic/erythroblastic islands are fragmented during tissue disaggregation and appear as remnants associated with unrelated cells on flow cytometry (Millard et al., 2021). Nevertheless, we detected a Ly6C-ve, F4/80+ve macrophage population in both WT and Fireko marrow that retained expression of CD115 in the mutant (Figure 1G,H)." What is this population? The progenitor mentioned before?

On "Mouse blood monocytes have been divided into classical and non-classical subpopulations based upon cell surface markers including Ly6C (Guilliams et al., 2018; Hettinger et al., 2013; Trzebanski et al., 2024)", the authors should rather refer to seminal studies that described such dichotomy for the first time?

Reviewer 3: SUMMARY OF THE ADVANCE MADE IN THIS PAPER AND ITS POTENTIAL SIGNIFICANCE TO THE FIELD

This manuscript presents a comprehensive extension of previous work on the Csf1rFIRE (Fireko) mouse line, expanding the characterization of macrophage deficiency phenotypes across tissues and incorporating AP1-element mutants as well as adoptive transfer experiments. The study addresses an important question for the mononuclear phagocyte community: how enhancer-level regulation of Csf1r controls macrophage development, maintenance, and functional responsiveness.

The topic is scientifically relevant and of interest to the macrophage field. The inclusion of both FIRE-deleted mice and AP1-site mutants provides mechanistic breadth, and the adoptive transfer-based repopulation data are valuable. However, the manuscript is currently very broad, with many independent models, tissues, assays, and readouts included. As a result, the paper reads somewhat

© 2026. Published by The Company of Biologists under the terms of the Creative Commons Attribution License (https://creativecommons.org/licenses/by/4.0/).

as a conglomeration of disparate components and would benefit from streamlining to clarify the central message and improve scientific cohesion.

Despite issues stated below, the dataset is rich and the core concept remains interesting. With significant revision, particularly improving quantitative rigor, clarifying claims, unifying nomenclature, adding missing controls, and focusing the narrative, the manuscript could make a meaningful contribution to understanding macrophage enhancer biology and tissue-specific CSF1R dependence.

SUGGESTIONS TO AUTHORS

Major points
1. the introduction reads partially like a review. please introduce only things that are important to understand this paper.
2. Figures 1G, H are not showing macrophage F4/80 signal the way it is described in the text
3. Figure 1: please provide representative gating for both WT and Fireko mice and show total numbers per leg/femure/tibia and not % of cells as showing % can be missleading.
4. Figure 1J: show cell numbers per ml of blood
5. the proper control for an CFS1-Fc injection is not saline but the corresponding IgG injection. therefore the results shown are interesting, but require repetition of proper controls
6. Figure 2B is impossible to interpret as the events shown differ so much between conditions. the same number of cells needs to be shown in each plot
7. Figure 2E: it seems that the addition of CSF2 also inhibits osteoclastogenesis in WT cells. It would be interesting to see whether not first the addition of Csf2, leading to the expression of Csf1r, and the subsequent addition of CSF1 would not rescue the penotype.
8. There is no Figure 3D. where is the increase of total yield shown?
9. "The peritoneal macrophage expansion in response to CSF1-Fc was significantly attenuated in the heterozygous mutant mice and the treatment did not overcome the macrophage deficiency of the Fireko." there is not data supporting this statement
10. Are the AP1 mice a new mouse line? if not, please add citation, if yes show the generation and validation of this line.
11. Figure 5A: figures are not of good enough quality to judge whether a microgliosis is present or not. could the authors please show high-resolution figures and add morphometry analyses for the different areas of the brain?
12.
13. Figure 5C: please include additional markers, e.g. CD11c or SiglecF for alveolar macrophages. it is not sufficient to conclude on macrophage subpopulations just by the cell shape
14. "Despite profound macrophage deficiency the kidneys of Fireko mice at all ages appear histologically normal with no evidence of glomerular deficiency or vascular impairment based on localization of the endothelial marker, CD31" this statement is not supported by data. a proper histopathological assessement needs to be performed. One cannot conclude anything on the CD31 staining as this is not of high resolution. Branching/volume of blood vascualture in 3D would need to be quantified to make such statements
15. Figure S5A: why was CD31 and other structures not stained in 30 week old mice?
16. Creatinine seems to be higher in Fireko. show single mice in bar graphs , it is compeltely unclear how many mice were use. were they male or female? which statistical tests were performed?
17. Figure S7C: how was pathology assessed? a pathologist should proved scoring of tissue during steady state and after injury
18. It would be of great interest to test whether the adoptive transfer can rescue the kidney pathology after injury to determine whether ontogeny plays a role in cellular / tissue responses

Minor points:

a) please remove the letters "ve" after - and + symbols in text and figures. this is irritating to read and is typically never used in other papers
b) Figure 1F: Bactin is probably beta-actin? correct this
c) "The responses of heterozygous mutant mice appeared intermediate between WT and Fireko.": this is only true for a part of readouts performed. Adjust sentence as the other values are either not altered or going even in the opposite direction

© 2026. Published by The Company of Biologists under the terms of the Creative Commons Attribution License (https://creativecommons.org/licenses/by/4.0/).

d) Figure 2a: what is the control? CSF1 WT mice are more than 100%, so this is not the control?
e) it is CD11b, not Cd11b. correct this
f) Please use everywhere the same naming of mice models, proteins etc. .e,g, Fireko, CSF1R FIRE KO/KO are just two of the names used for the same mouse model.
g) write into all images which antibody was used, not only in the legend
h) in the discussion the authors mention that macrophages in the intestine are continuously replenished from blood monocytes. However, this is certainly not the case for all parts of the gut. And looking into the citation given, this is not what is written in this review. The authors should correct this.
i) Figure 7B is not showing CD11b expression differences across genotypes

---

Resubmission

Response to Original Reviews

Reviewer 1: SUMMARY OF THE ADVANCE MADE IN THIS PAPER AND ITS POTENTIAL SIGNIFICANCE TO THE FIELD

In this report, the authors employed the Fireko mouse model to address the effect of the Csf1r deltaFIRE mutation on monocytopoiesis, CSF1 responsiveness and regulation of Csf1r expression and signaling. They also assessed macrophage abundance across organs, function of renal and heart macrophages, and repopulation following adoptive transfer of wild-type cells in Fireko mice. The manuscript is well written, the experiments are well executed, the long discussion reflects the different aspects evaluated in this report. Few suggestions for improvement can be found below.

We thank the reviewer for the positive comments.

SUGGESTIONS TO AUTHORS

1. The study infers preserved monocytopoiesis based on steady-state monocyte counts without directly assessing production or egress rates from the bone marrow. However, the population snapshots shown in Figure 1 (progenitor frequencies and monocyte counts) cannot distinguish normal production from compensatory mechanisms such as prolonged monocyte lifespan, altered egress, or extramedullary production. Without kinetic measurements, the conclusion that monocytopoiesis proceeds independently of CSF1R in progenitors remains speculative. This limitation impacts the interpretation of Figure 1 datasets.

We have restated the conclusion in the results more precisely.

"Monocytopoiesis does not absolutely require CSF1R expression in progenitor cells".

We have added data in new Fig S1 showing that monocytes are not depleted in the bone marrow, nor do they accumulate in the spleen in Fireko mice. We also demonstrate the shift in the relative proportions of Ly6Chigh/Ly6Clow subpopulations in marrow, spleen and blood.

In results and discussion we have placed our findings more firmly in context of prior knowledge of CSF1/CSF1R regulation of monocytopoiesis.

"*Fireko* mice lack *Csf1r* expression in bone marrow progenitors and monocytes and are unresponsive to CSF1 *in vitro* or *in vivo* but they are not monocyte-deficient in marrow or blood. This observation is consistent with previous evidence that a blocking anti-CSF1R antibody does not deplete monoblasts in the marrow {Sudo,1995} or blood monocytes {MacDonald, 2010;Louis, 2015}. "

In bone, *Fireko* mice are not osteoclast-deficient {Rojo, 2019}. The analysis in **Figure 1G** indicates that F4/80^{+} hematopoietic island macrophages are also retained and can express CSF1R, by contrast to Ly6C^{high} marrow monocytes. Our findings are actually consistent with two recent studies showing

© 2026. Published by The Company of Biologists under the terms of the Creative Commons Attribution License (https://creativecommons.org/licenses/by/4.0/).

that conditional deletion of *Csf1* expression in marrow adipogenic stromal cells leads to loss of osteoclasts and resident macrophages without affecting monocytes {Zhong, 2023;Inoue, 2023}.

2. Several organ-wide "absence" claims rely primarily on reporter imaging without comprehensive, reporter-independent validation or quantitative cell counts. Dependence on Csf1r-EGFP reporter expression risks missing macrophages with reduced CSF1R or altered F4/80 expression, leading to false negatives, particularly in tissues such as pancreas, adipose, and skeletal muscle. As established in lineage tracing studies, reporter-based methods alone can underrepresent cell populations due to technical limitations. Orthogonal validation (e.g., alternative markers, immunostaining, or flow cytometry) is essential for robust conclusions. Without such controls, the assertion that these tissues lack macrophages may overstate depletion and weakens the inference of tissue-selective loss based solely on reporter signal. It is also important to note that Csf1r-EGFP reporter mice depend on FIRE enhancer activity, further influencing expression patterns.

The Csf1r-EGFP transgene has been very widely used in both mice and rats and there is no evidence of acute regulation. It is a multicopy transgene inserted into the genome independently of the *Csf1r* locus, so there is no mechanism for direct effect of the *Fireko* on transgene expression.

The original and subsequent papers dealing with the *Fireko* provided FACS analysis and IHC for markers of subpopulations in brain, kidney, heart, skin and peritoneum. The submitted manuscript already contained IHC for F4/80 on multiple tissues complementing the detection of the EGFP transgene. We have added IHC/IF data with appropriate quantification for F4/80/IBA1 for multiple additional organs including pituitary, salivary gland, pancreas, adrenals, gonads and adipose (Figure S5). These findings add significant depth and novelty to the work.

The fact that the missing Csf1r-EGFP cells subpopulations in multiple tissues are replaced selectively by CSF1R-FRed expressing donor cells following BMT provides additional validation.

3. The breadth and completeness of macrophage repopulation are predicted from qualitative imaging of selected tissues without systematic, quantitative coverage across organs and macrophage subsets. Some subsets remain host-derived (e.g., alveolar macrophages and Kupffer cells), and only the F4/80high population was replaced in kidney. Therefore, the claim of "complete" repopulation across all organ niches is not fully supported by the data. This concern is reinforced by known heterogeneity of tissue-resident macrophages, including functionally distinct subsets within individual organs, and by evidence that developmental origin influences repopulation dynamics. Furthermore, donor-derived brain macrophages exhibit altered marker expression and morphology, suggesting phenotypic differences that complicate the notion of equivalent replacement. Without organ-wide quantitative counts benchmarked to wild-type densities for each subset, generalizations such as "every organ" and "complete".

Figure 6 compares the density and location of *Csf1r*-EGFP^{+} cells in multiple tissues of WT mice and FRed^{+} cells in *Fireko* mice receiving BMT. In the specific case of the brain both Flow Cytometry and Immunolocalization (Figures 7B, 7C) support the conclusion that the density of donor-derived cells in BMT recipient Fireko mice is similar to microglial density in a wild-type mouse but nevertheless they are functionally distinct. In the kidney, we show that the Cd11b^{low}/F4/80^{high} resident population that is absent in Fireko mice is selectively populated by FRed^{+} donor cells (Figure S10C).

The new data added in Figure S5 adds an additional dimension to the BMT study. The analysis of the brain had already indicated that brain-associated macrophages, which are retained in *Fireko* mice, are nevertheless replaced by donor cells. We now recognise that donor cells also replace the residual recipient macrophages in muscle, ovary, testis, salivary gland, pancreas and adipose.

The most important conclusion is that donor cell contribution does not involve sustained blood cell chimerism. The key sentence in the revised abstract now reads:

Tissue resident macrophages that were depleted in *Fireko* mice, including microglia, were replaced by donor-derived cells following intraperitoneal adoptive transfer of wild-type bone marrow at weaning.

We feel that this statement is fully supported by the data presented.

© 2026. Published by The Company of Biologists under the terms of the Creative Commons Attribution License (https://creativecommons.org/licenses/by/4.0/).

Minor Comments

Page 6, line 6: Figure 1 does not show data for CMP and GMP, though these are mentioned in the text.

Agreed. We have removed the comment in the text and added specific reference to expression of CSF1R in MPP3 as the rationale for the analysis.

Page 7, line 1: Should state 37 °C instead of 4 °C.

Actually this is correct but the text was misleading and has been corrected.
Page 8, line 13: According to Figure 2, there is a decrease in MHCII high cells (not an increase) and an increase in MHCII low cells (not a decrease).

Agreed. This has been corrected
Page 8, line 17 and Figure 2 legend: E. coli should be italicized.

Corrected
Page 20, line 11: Missing period at the end of the sentence.
Corrected
Comments on Figures

Panel annotations vary (sometimes "A." vs "A" vs bold "A"); please maintain a consistent style.
Corrected

Bar graphs: group distinctions use both shades of gray and symbols inconsistently; choose one system for clarity.
Figure 2 legend: Refers to "CSF1R MFI," while the figure shows "CD115 MFI"; harmonize terminology.
Figure 4C: Gene names should be italicized.
Figure 4B: Missing legend.
Figure 5A: Missing scale bar.
Figure 5B: Missing legend.
Figure 6: Scale bar length not specified.
Figure S4B: Scale bar length not specified.

Corrected

Reviewer 2: This study investigates the role of the conserved FIRE enhancer within the Csf1r locus, which regulates macrophage development and CSF1R signalling. Mice lacking FIRE exhibit widespread loss of tissue resident macrophages, including microglia and macrophages in the heart, kidney, and other organs, though monocyte numbers remain intact. Using a new congenic Fireko line with a Csf1r-EGFP reporter, the authors show that FIRE is dispensable for Csf1r expression in GM-CSF-responsive cells and inflammatory macrophages but essential for CSF1-driven transcription. Mutation of the conserved AP1 site within FIRE further impaired CSF1 responsiveness, underlining that CSF1 signalling depends on the rate of CSF1R synthesis. Finally, macrophage-deficient tissues in Fireko mice could be repopulated by adoptive transfer of wild-type bone marrow cells without conditioning.

The study is interesting in principle but is superficial, descriptive and correlative in nature. There is a lack of real connection between the experiments done, from BM to peritoneum to kidney, with a last part dealing with repopulation with BM transfer. There is no real clear messages and some conclusions are quite strong with no real data supporting them. In this regard, the last part claiming the presence of a CSF1R-dependent macrophage progenitor population in the BM with high proliferative potential that gives rise directly to resident tissue macrophages is weak and require further work to be supported. A lot of cofounding factors such as the lack of comparators (monocytes?), the site of injection as well as the Fireko background could be a play.

The selective macrophage deficiency in the *Fireko* mice has generated a great deal of interest in the macrophage community. This work covers a lot of ground. We do not feel the work is superficial, descriptive and correlative but we acknowledge that the take home messages were not

© 2026. Published by The Company of Biologists under the terms of the Creative Commons Attribution License (https://creativecommons.org/licenses/by/4.0/).

entirely clear. To make the logic and connectivity of the findings clearer we have changed the title, rewritten the abstract and changed the titles of each of the sections in the results to definitive statements of the conclusions.

We do agree that the nature of the progenitor in the marrow transfer experiments is unclear. We have moved this speculation from the introduction to the discussion and added reference to the common monocyte progenitor. Comparison with monocytes as donors is not practical given the numbers of Fireko mice we can generate but we have added reference to two recent studies on microglial repopulation by monocytes. Our data shows that repopulation is sustained without detectable contribution to monocytes.

The authors mentioned "F4/80high bone marrow resident macrophages associated with hematopoietic/erythroblastic islands are fragmented during tissue disaggregation and appear as remnants associated with unrelated cells on flow cytometry (Millard et al., 2021). Nevertheless, we detected a Ly6C-ve, F4/80+ve macrophage population in both WT and Fireko marrow that retained expression of CD115 in the mutant (Figure 1G,H)." What is this population? The progenitor mentioned before?

We agree that this was unclear and have modified the text accordingly. The issue is that resident macrophages in spleen and marrow are fragmented during tissue disaggregation and are attached as remnants to unrelated cells.

On "Mouse blood monocytes have been divided into classical and non-classical subpopulations based upon cell surface markers including Ly6C (Guilliams et al., 2018; Hettinger et al., 2013; Trzebanski et al., 2024)", the authors should rather refer to seminal studies that described such dichotomy for the first time?
We agree and we have altered the references accordingly.

Reviewer 3: SUMMARY OF THE ADVANCE MADE IN THIS PAPER AND ITS POTENTIAL SIGNIFICANCE TO THE FIELD

This manuscript presents a comprehensive extension of previous work on the Csf1rFIRE (Fireko) mouse line, expanding the characterization of macrophage deficiency phenotypes across tissues and incorporating AP1-element mutants as well as adoptive transfer experiments. The study addresses an important question for the mononuclear phagocyte community: how enhancer-level regulation of Csf1r controls macrophage development, maintenance, and functional responsiveness.

The topic is scientifically relevant and of interest to the macrophage field. The inclusion of both FIRE-deleted mice and AP1-site mutants provides mechanistic breadth, and the adoptive transfer-based repopulation data are valuable. However, the manuscript is currently very broad, with many independent models, tissues, assays, and readouts included. As a result, the paper reads somewhat as a conglomeration of disparate components and would benefit from streamlining to clarify the central message and improve scientific cohesion.

Despite issues stated below, the dataset is rich and the core concept remains interesting. With significant revision, particularly improving quantitative rigor, clarifying claims, unifying nomenclature, adding missing controls, and focusing the narrative, the manuscript could make a meaningful contribution to understanding macrophage enhancer biology and tissue-specific CSF1R dependence.

SUGGESTIONS TO AUTHORS

Major points
1. the introduction reads partially like a review. please introduce only things that are important to understand this paper.

We have substantially condensed the introduction and the discussion.
2. Figures 1G, H are not showing macrophage F4/80 signal the way it is described in the text

Agreed. We have modified the text to make this clearer.

© 2026. Published by The Company of Biologists under the terms of the Creative Commons Attribution License (https://creativecommons.org/licenses/by/4.0/).

3. Figure 1: please provide representative gating for both WT and Fireko mice and show total numbers per leg/femure/tibia and not % of cells as showing % can be missleading.

4. Figure 1J: show cell numbers per ml of blood
We have added the gating Figure within a new Figure S1.
We have added the following preamble to the results section.
Fireko mice are not osteopetrotic and the mutation has no significant effect on total white cell, red cell or platelet count expressed/ml blood or on relative neutrophil, lymphocyte or monocyte counts defined by automatic analyser {Rojo et al. 2019}.
Given this background, we do not feel that showing the number of cells as a percentage is misleading.
5. the proper control for an CFS1-Fc injection is not saline but the corresponding IgG injection. therefore the results shown are interesting, but require repetition of proper controls

We disagree. IgG would not be the appropriate control since CSF1-Fc is a fusion protein. The ability of CSF1 to produce monocytosis is not an issue; it was shown many years ago using recombinant CSF1 (PMID: 3053899) and the original description of CSF1-Fc activity demonstrated that the major effect of the fusion protein was to increase the half-life and hence the efficacy (PMID: 24962162). Arguably the saline injection control is not necessary but it eliminates non-specific effects of the stress of handling and injection. The key point is that Fireko mice are resistant to the effect of CSF1-Fc.

6. Figure 2B is impossible to interpret as the events shown differ so much between conditions. the same number of cells needs to be shown in each plot

We agree. The figure panel has been removed.
7. Figure 2E: it seems that the addition of CSF2 also inhibits osteoclastogenesis in WT cells. It would be interesting to see whether not first the addition of Csf2, leading to the expression of Csf1r, and the subsequent addition of CSF1 would not rescue the penotype.

That would seem like a diversion. What we conclude is that CSF2 is not the factor that permits OCL formation.
8. There is no Figure 3D. where is the increase of total yield shown?
This was an error. The figures were contained in the same panels as the response to thioglycolate. It has been corrected.
9. "The peritoneal macrophage expansion in response to CSF1-Fc was significantly attenuated in the heterozygous mutant mice and the treatment did not overcome the macrophage deficiency of the Fireko." there is not data supporting this statement

Data is in Figure 3B,C. Text and legend have been edited.
10. Are the AP1 mice a new mouse line? if not, please add citation, if yes show the generation and validation of this line.
Yes, it is new. The text has been modified to make this clear.

11. Figure 5A: figures are not of good enough quality to judge whether a microgliosis is present or not. could the authors please show high-resolution figures and add morphometry analyses for the different areas of the brain?

The images have been replaced with higher resolution which is sufficient to demonstrate that the heterozygous mutation does not alter the morphology of the microglia. We are showing a small reduction in microglia in heterozygous mice at 3 weeks, not a microgliosis. This is quantified in 3 brain regions in Panel 5B.
12.
13. Figure 5C: please include additional markers, e.g. CD11c or SiglecF for alveolar macrophages. it is not sufficient to conclude on macrophage subpopulations just by the cell shape.
We do not agree that additional markers are required to support the conclusions. The lack of effect of the Fireko on SiglecF+ AM analysed by Flow Cytometry was reported previously and is now referenced more explicitly. The expression of the *Csf1r*-EGFP transgene by AM is known. We have added reference to an earlier study in *Development* that used the *Csf1r*-EGFP transgenic line to

© 2026. Published by The Company of Biologists under the terms of the Creative Commons Attribution License (https://creativecommons.org/licenses/by/4.0/).

study macrophage development in the embryo and the early postnatal period (Tan & Krasnow, 2016).
14. "Despite profound macrophage deficiency the kidneys of Fireko mice at all ages appear histologically normal with no evidence of glomerular deficiency or vascular impairment based on localization of the endothelial marker, CD31" this statement is not supported by data. a proper histopathological assessement needs to be performed. One cannot conclude anything on the CD31 staining as this is not of high resolution. Branching/volume of blood vascualture in 3D would need to be quantified to make such statements
15. Figure S5A: why was CD31 and other structures not stained in 30 week old mice?

In the context of the adenine diet model, we showed that *Fireko* kidneys were of normal size and histologically indistinguishable as evaluated by a qualified veterinary pathologist. CD31 staining was included at 3 and 10 weeks with the expectation based upon analysis of CSF1R knockout rats that we might see an effect of macrophage deficiency on development of glomeruli or medulla. This is a qualitative assessment. The rationale has been clarified in the text,
16. Creatinine seems to be higher in Fireko. show single mice in bar graphs , it is compeltely unclear how many mice were use. were they male or female? which statistical tests were performed?
We decided to remove this Figure. The key variables, serum creatinine and urea, were measured in larger male and female cohorts analysed as controls in the context of the adenine diet.
17. Figure S7C: how was pathology assessed? a pathologist should proved scoring of tissue during steady state and after injury
This was provided in Materials & Methods. Pathology was assessed by a qualified Veterinary Pathologist who was blinded to the sample identity. This has been added to the legend.
18. It would be of great interest to test whether the adoptive transfer can rescue the kidney pathology after injury to determine whether ontogeny plays a role in cellular / tissue responses
We actually show that the absence of macrophages in the *Fireko* has a very limited impact on renal pathology. Hence, it would be difficult to show an effect of repopulation by adoptive transfer. We have added reference to the ontogeny of kidney macrophages.

Minor points:

a) please remove the letters "ve" after - and + symbols in text and figures. this is irritating to read and is typically never used in other papers
Done

b) Figure 1F: Bactin is probably beta-actin? correct this
Done
c) "The responses of heterozygous mutant mice appeared intermediate between WT and Fireko.": this is only true for a part of readouts performed. Adjust sentence as the other values are either not altered or going even in the opposite direction
Agreed. This is a minor point and the sentence has been removed.

d) Figure 2a: what is the control? CSF1 WT mice are more than 100%, so this is not the control?
In the original Figure, all results were normalised to one of the WT controls. We agree this is confusing. It was shown previously that bone marrow cells from *Fireko* mice do not grow in CSF1. The only take-home point in this Figure was to show that bone marrow cells from *Fireko* mice grow in CSF2. The more important point is that *Fireko* bone marrow cells grown in CSF2 express detectable CSF1R. We have removed panels A and B from the revised figure.

e) it is CD11b, not Cd11b. correct this

Corrected in Figures
f) Please use everywhere the same naming of mice models, proteins etc. .e,g, Fireko, CSF1R FIRE KO/KO are just two of the names used for the same mouse model.

We have modified to be consistent in the text. In Figures where WT, Het and homozygous mice are compared we have altered to consistently use FIRE WT/WT, FIRE Δ/WT and FIRE Δ/Δ

g) write into all images which antibody was used, not only in the legend

© 2026. Published by The Company of Biologists under the terms of the Creative Commons Attribution License (https://creativecommons.org/licenses/by/4.0/).

Done
h) in the discussion the authors mention that macrophages in the intestine are continuously replenished from blood monocytes. However, this is certainly not the case for all parts of the gut. And looking into the citation given, this is not what is written in this review. The authors should correct this.
We have clarified to refer specifically to lamina propria and the original reference has been added.

i) Figure 7B is not showing CD11b expression differences across genotypes
Agreed. This has been corrected.

First decision letter

MS ID#: dev.205655

MS TITLE: Depletion and replacement of tissue resident macrophages in mice with germ-line deletion of a conserved enhancer in the Csf1r locus.

AUTHORS: Yajun Liu, Sebastien Jacquelin, Isis Taylor, Emma K. Green, Omkar L. Patkar, Sahar Keshvari, Ginell Ranpura, Conan J. O. O'Brien, Eline Jessen, Emma Maxwell, Rachel Allavena, Alexandre Gallerand, Stoyan Ivanov, Antony Adamson, Neil E. Humphreys, Kim M. Summers, Katharine M. Irvine and David A. Hume

Dear Dr Hume,

I have now received all the referees' reports on the above manuscript, and have reached a decision. The referees' comments are appended below.

As you will see, the referees express interest in your work, but have some significant criticisms and recommend a substantial revision of your manuscript before we can consider publication. The referees recognise that some of the issues raised in the previous round of reviews have been addressed. Nevertheless, there remain significant criticisms that preclude publication of this version of the study. The revision is difficult to evaluate because changes are not marked in the manuscript (no track changes, highlighting, or line numbers) and Reviewer 2 has concerns about apparent inconsistencies between the response letter and the revised manuscript: data stated to have been removed remain present, while other data appear to have been removed rather than clarified. This raises concerns about the completeness and transparency of the presented evidence, particularly given ongoing debate in the field regarding macrophage contributions to tissue development. Multiple substantive points from the previous round remain inadequately addressed: macrophage identity in Figure 1G should be confirmed with appropriate markers; quantification of cell populations relies solely on percentages rather than total numbers, and monocyte gating strategies are incorrect and non-standard; the CSF1R-Fc experiment lacks an appropriate species-matched control protein, which is essential given its human origin and potential immunogenic effects in mice. Statistical testing is absent for key claims; alveolar macrophage identity has not been confirmed by co-staining; and data in Figure S7E showing a clear effect in KO males are unreferenced in the text. If you are able to revise the manuscript to address these points, which may involve further experiments, I will be happy to receive a revised version of the manuscript.

Your revised paper will be re-reviewed by one or more of the original referees, and acceptance of your manuscript will depend on your addressing satisfactorily the reviewers' major concerns. Please also note that Development will normally permit only one round of major revision. If it would be helpful, you are welcome to contact us to discuss your revision in greater detail. Please send us a point-by-point response indicating your plans for addressing the referees' comments, and we will look over this and provide further guidance.

Please attend to all of the reviewers' comments and ensure that you upload both a 'clean' version of your Word file, along with a highlighted version clearly showing where you have made changes in

© 2026. Published by The Company of Biologists under the terms of the Creative Commons Attribution License (https://creativecommons.org/licenses/by/4.0/).

the revised manuscript. Please avoid using 'Tracked changes' in Word files as these are lost in PDF conversion. I should be grateful if you would also provide a point-by-point response detailing how you have dealt with the points raised by the reviewers in the 'Response to Reviewers' box. If you do not agree with any of their criticisms or suggestions please explain clearly why this is so.

Reviewer 1

SUMMARY OF THE ADVANCE MADE IN THIS PAPER AND ITS POTENTIAL SIGNIFICANCE TO THE FIELD

In this report, the authors employed the Fireko mouse model to address the effect of the Csf1r deltaFIRE mutation on monocytopoiesis, CSF1 responsiveness and regulation of Csf1r expression and signaling. They also assessed macrophage abundance across organs, function of renal and heart macrophages, and repopulation following adoptive transfer of wild-type cells in Fireko mice.

SUGGESTIONS TO AUTHORS

The authors have addressed all of my comments by providing new data, improving the explanation of their results, and moderating their language.

Minor comments
Page 2: "Here we show that loss of CSF1R expression in Fireko mice is partly overcome by CSF2 in vitro and inflammatory recruitment in vivo"
Page 3: FIRE is abbreviated but not written out in full at first mention.
Page 4: Hydrocephalus was previously written in full, but here only the abbreviation is used. Please be consistent.
Page 6: In the sentence "shows the profile of CD115 detection in from WT and Fireko mice," a panel reference is missing. There also appears to be an extra "in."
Page 7: Osteoclast was previously written in full, but here only the abbreviation is used. In addition, both the full term and the abbreviation appear in the text. Please use one format consistently

Reviewer 2

The authors have partially revised the manuscript, but are not addressing many of the points previously made. Therefore, the major issues are pointed out below. Further, the absence of clearly indicated revisions in the manuscript is not acceptable. It is currently difficult to identify which changes have been made without comparing multiple document versions side by side. The authors should ensure that all revisions are clearly marked in the next version, for example by using track changes or by highlighting modified text. Please use also line numbers so that it is easy to refer to certain text parts

1. Figure 1G is still not showing F4/80+ macrophages. (see previous point 2)

2. Points 3+4 are simply discussed away. Not state-of-the art to show only % of cells. Total numbers are necessary as many other things but the lack of osteopetrosis can happen. Also, the new gating provided is by no means the correct way to gate on Ly6Chigh/lo monocytes. Please check recent papers and how to exclude CD11b and Ly6C expressing progenitors in the spleen and bone marrow and use positive markers for Ly6Clow monocytes, not this random gate shown in Figure S1B and C

3. Previous point 5 regarding CSF1R-Fc: one of the papers cited to argue against a control injection with another protein refer to a pig CSF1-Fc. But I assume that a human protein was used here.

4. The fact that similar approaches may have been published without appropriate controls does not justify their omission here. Proper controls are essential and should not be overlooked. The CSF1R-Fc used in this study is a fusion protein of human origin according to the methods, and therefore an appropriate species-matched control protein should be included, particularly given the potential impact on immune responses in mice.

© 2026. Published by The Company of Biologists under the terms of the Creative Commons Attribution License (https://creativecommons.org/licenses/by/4.0/).

5. Significance should be tested for this statement, especially if the data is shown in one plot "CSF1-Fc treatment caused an increase in total yield of lavage cells and an increase in

6. the % of large peritoneal macrophages within the lavage in WT mice."

7. Previous point 10 has not been fully addressed: show gene locus, type of mutation, genotyping protocols and validation of new AP1 mouse model, especially if this is the first paper that is using this new mouse line.

8. Previous point 13: not acceptable. Perform co-staining with at least 1 alveolar macrophage marker to show that the GFP signals overlaps 100% with this marker.

9. Previous point 16: the figure has not been removed, and is shown in S7E, where it should remain, or even go to the main figures as it shows a clear effect in KO male mice. This figure has been omitted in the text. It is questionable why the authors would want to remove such significant data?

10. Previous point 18: in light with my previous point, I would like to see this experiment.

First revision

Author response to reviewers' comments

Reviewer 1: SUMMARY OF THE ADVANCE MADE IN THIS PAPER AND ITS POTENTIAL SIGNIFICANCE TO THE FIELD

In this report, the authors employed the Fireko mouse model to address the effect of the Csf1r deltaFIRE mutation on monocytopoiesis, CSF1 responsiveness and regulation of Csf1r expression and signaling. They also assessed macrophage abundance across organs, function of renal and heart macrophages, and repopulation following adoptive transfer of wild-type cells in Fireko mice.

SUGGESTIONS TO AUTHORS

The authors have addressed all of my comments by providing new data, improving the explanation of their results, and moderating their language.

Minor comments
Page 2: "Here we show that loss of CSF1R expression in Fireko mice is partly overcome by CSF2 in vitro and inflammatory recruitment in vivo"
Page 3: FIRE is abbreviated but not written out in full at first mention.
Page 4: Hydrocephalus was previously written in full, but here only the abbreviation is used. Please be consistent.
Page 6: In the sentence "shows the profile of CD115 detection in from WT and Fireko mice," a panel reference is missing. There also appears to be an extra "in."
Page 7: Osteoclast was previously written in full, but here only the abbreviation is used. In addition, both the full term and the abbreviation appear in the text. Please use one format consistently

We thank the reviewer for the comments. All of these corrections have been made.

Reviewer 2: The authors have partially revised the manuscript, but are not addressing many of the points previously made. Therefore, the major issues are pointed out below. Further, the absence of clearly indicated revisions in the manuscript is not acceptable. It is currently difficult to identify which changes have been made without comparing multiple document versions side by side. The authors should ensure that all revisions are clearly marked in the next version, for example by using track changes or by highlighting modified text. Please use also line numbers so that it is easy to refer to certain text parts

© 2026. Published by The Company of Biologists under the terms of the Creative Commons Attribution License (https://creativecommons.org/licenses/by/4.0/).

We apologise for the lack of the track changes version. We understood this version would be considered as a new submission. We have added line numbers to the latest revised version and provided a track changes version.
In response to the reviewer's and editor's comments we have rewritten the abstract.
We have also condensed the discussion substantially.

The key findings in this study are:

- The effect of the *Fireko* on bone marrow on the C57BL/6 background is consistent with prior analysis on a mixed background.
- *Fireko* mice lack CSF1R in progenitors and marrow monocytes but monocytopoiesis and monocyte maturation are unaffected.
- CSF1R expression is retained in F4/80+ resident marrow macrophages which are not depleted in the mutant.
- CSF1R is expressed in *Fireko* mice at reduced levels in Ly6C^{low}/F4/80^{+} blood monocytes, in inflammatory macrophages and in macrophages grown in CSF2.
- *Fireko* mice are resistant to CSF1 *in vitro* and to a CSF1-Fc fusion protein *in vivo*.
- *Fireko* mice lack tissue-resident macrophages detectable using a *Csf1r*-EGFP transgene in multiple organs, extending previous analysis.
- Heterozygous *Fireko* impacts CSF1 response *in vitro* and postnatal expansion of tissue-resident macrophages.
- Mutation of the highly-conserved AP1 element within FIRE also impacts CSF1R responsiveness *in vitro* but does not recapitulate the *Fireko* phenotype.
- The absence of resident macrophages in kidney and heart has no detectable impact on development or physiological function.
- In a model of tubular injury, the absence of renal resident macrophages in the *Fireko* and lack of CSF1R expression in bone marrow has no impact on body weight loss, macrophage recruitment or histopathology.
- Resident macrophage populations that are absent in the *Fireko* mice are repopulated by donor-derived cells following transfer of wild-type bone marrow cells at weaning. Donor cells do not contribute to the blood monocyte pool.

We have addressed the concerns raised below. Some are matters of opinion where we disagree. Most importantly, none of the concerns invalidates any of the above conclusions.

RESPONSES

1. Figure 1G is still not showing F4/80+ macrophages. (see previous point 2)

We recognise that the relationship between Figures 1G and 1H was unclear in both the` text and the Figure legend. This has been corrected. The key conclusion is that the Ly6C^{-} cells that retain CD115 (CSF1R) in the *Fireko* marrow are also F4/80^{+} consistent with their identity as resident marrow macrophages.

2. Points 3+4 are simply discussed away. Not state-of-the art to show only % of cells. Total numbers are necessary as many other things but the lack of osteopetrosis can happen. Also, the new gating provided is by no means the correct way to gate on Ly6Chigh/lo monocytes. Please check recent papers and how to exclude CD11b and Ly6C expressing progenitors in the spleen and bone marrow and use positive markers for Ly6Clow monocytes, not this random gate shown in Figure S1B and C

In presenting the analysis of bone marrow, blood and spleen we failed to clearly indicate what was already known and the purpose of the analysis; to confirm that the basic myeloid phenotype of the Fireko was not substantially different on the inbred C57BL/6J background compared to the mixed background studied in the original line. We have rewritten parts of the results to make this clear.

The total bone marrow cellularity and the cellular composition of the marrow expressed as a %

© 2026. Published by The Company of Biologists under the terms of the Creative Commons Attribution License (https://creativecommons.org/licenses/by/4.0/).

are separate variables. In saying that the *Fireko* mice were not osteopetrotic in the previous text we meant to indicate that the total bone marrow cellularity is unchanged in the *Fireko* compared to WT. Bone marrow cellularity obtained by flushing the femur was quantified in the original description of the *Fireko* and was not affected by the mutation. We have confirmed this finding in the C57BL/6 line in new Figure S1B. We have added a more explicit statement that the marrow of the *Fireko* mouse is histologically normal and total number of bone marrow cells obtained by flushing the long bones is not changed.

Whilst we are aware that some groups choose to normalise results to cells/femur (e.g. PMID:39089257 (on which DAH is an author); PMID:35508166 (which we have added as a reference)), that is not universal. In those examples, there is evidence for a change in total cellularity as a consequence of conditional CSF1 deletion. In choosing to present data as cells/femur, authors conflate three variables, the technical variation in recovery of bone marrow cells, the genuine variation in bone marrow cellularity and the variation in cellular composition. We are interested in the effect of the *Fireko* on myeloid differentiation. In the absence of any significant change in total cellularity, we feel it is more informative to display the results in terms of cell composition, as for example the original paper identifying the common monocyte progenitor (PMID: 23812096; see Figure 1) or the more recent analysis of classical monocyte heterogeneity (PMID:38749446). We have made this rationale clear in the revised manuscript.

We recognise that there are other ways to gate monocytes and monocyte subsets but we disagree that there is a single “correct’ way. We failed to explain the rationale for the gating which has now been corrected in the text. In common with the original paper describing the *Fireko*, in the blood, marrow and spleen we have used F4/80 as an additional positive marker to distinguish the Ly6C^{low} subset of CD11b^{+}/Ly6G^{-} cells from NK cells and progenitors as previously described (PMID: 20682855). CD115 is commonly used as the most definitive marker of monocytes in both bone marrow and blood (e.g. PMID: 28514690; PMID: 23812096;PMID:38749446) but our purpose was to measure the effect of the *Fireko* on CSF1R expression. In the revised text and in Figure 1 and Figure S1 we have referred to populations of cells more explicitly in terms of the markers used. The key novel conclusion shown in Figure 1G and Figure 1K is that there is selective loss of CD115 from the Ly6C^{high} population in both BM and blood where expression is detected in Ly6C^{low} populations and retained in Ly6C^{-}/F4/80^{+} resident marrow macrophages.
The F4/80 gate in Figure S1B and S1C was not random. There are two issues with using positive markers to define non-classical monocytes in immune tissues. (1) markers such as CX3CR1 and CD43 are expressed by other cell types including tissue-resident macrophages; (2) Resident macrophages are fragmented during tissue disaggregation and the F4/80^{+} population on flow cytometry consists mainly of macrophage remnants attached to other cells (PMID:34818538). We defined Ly6C$^{low/-}$ monocytes as F4/80^{low} to exclude F4/80^{high} mature macrophages. However, in both BM and spleen the Ly6C^{-} population is likely made up primarily of F4/80^{+} remnants of disaggregated tissue- resident macrophages. We have therefore reanalysed the flow data to exclude the Ly6C^{-} cells. This does not change the conclusion, now restated more explicitly, that the lack of detectable CSF1R in progenitors and classical monocytes does not compromise the development of Ly6C^{low} monocytes. That conclusion is consistent with the original paper describing the *Fireko* and a preprint from co-authors (PMID: 40777406).

3. Previous point 5 regarding CSF1R-Fc: one of the papers cited to argue against a control injection with another protein refer to a pig CSF1-Fc. But I assume that a human protein was used here.

4. The fact that similar approaches may have been published without appropriate controls does not justify their omission here. Proper controls are essential and should not be overlooked. The CSF1R-Fc used in this study is a fusion protein of human origin according to the methods, and therefore an appropriate species-matched control protein should be included, particularly given the potential impact on immune responses in mice.

We do not agree with this comment.

© 2026. Published by The Company of Biologists under the terms of the Creative Commons Attribution License (https://creativecommons.org/licenses/by/4.0/).

The reviewer previously suggested we inject human IgG as a control. If we were trying to define the mechanism of action of CSF1-Fc, which we are not, a more relevant control might be a fusion between an inactive mutant human CSF1 and mouse Fc. If we could make such a protein, injection into mice would be a new experiment which would require animal ethics approval. We would still need to do a saline control.

CSF1-Fc is a drug. The proper control to determine whether a mouse has responded to CSF1-Fc is a mouse of the same age, sex and genotype that has not received CSF1-Fc but has received the vehicle, in this case saline. As with any drug, there is the potential for off target effects. To address that possibility, the additional information required is that the effect of drug administration is consistent with and dependent upon interaction with the drug target, in this case, CSF1R.

The administration of recombinant human CSF1 to mice was shown to cause selective monocytosis and expansion of tissue macrophages when the CSF1 gene was first cloned in the 1980s (PMID: 3053899) as a prelude to human clinical trials which confirmed the selective effects (reviewed in PMID: 34742624). Subsequently, injection of recombinant human CSF1 was shown to overcome the macrophage deficiency in the *Csf1*$^{op/op}$ mouse (PMID: 8050349). Pig CSF1 is also active in mice and we tested the pig CSF1-Fc in mice before going on to demonstrate that the same response was observed in pigs (PMID:27445344) and rats (PMID: 31397014). We used labelled pig CSF1-Fc ligand as an alternative to anti-CD115 to confirm the loss of CSF1R in monocytes of *Fireko* mice and *Csf1rko* rats (thereby confirming the binding specificity). We have also traced the fate of the AF647-labelled pig CSF1-Fc fusion protein in mice and shown that it traffics specifically to macrophages and binding is inhibited by anti-CSF1R (PMID: 29440354)

Fc fusion proteins are very widely used to increase circulating half-life of therapeutic proteins. Human-Fc fusions are commonly tested in rodents and the outcome is interpreted in terms of the known biology of the ligand. For example, human G-CSF Fc promoted granulocytosis in rats (PMID: 15145212) and is now in clinical use, human EPO-Fc was first tested as treatment for acute anaemia in rats (PMID: 23977338) and a human prolactin-Fc fusion promotes galactopoiesis in mice (PMID: 40792031). Each of these studies used a vehicle (saline) control.

To address this comment in the revised text, we have clarified in the Methods the precise nature of the recombinant protein (Human CSF1:mouse IgG2a Fc fusion with LALA mutation to prevent Fc receptor binding) we used and referenced the fact that human CSF1 is active on mouse CSF1R. The text now reads;

“Bone marrow cells from Fireko mice do not respond to CSF1 in vitro {Rojo et al, 2019}. To confirm the CSF1 unresponsiveness of Fireko mice, we injected a human CSF1- mouse IgG2A Fc fusion protein. Recombinant human CSF1 injection in mice causes monocytosis and expansion of tissue macrophages {Hume et al, 1988}; the fusion to Fc increases efficacy by extending the otherwise short circulating half-life. Administration of the mouse-human CSF1-Fc or a pig CSF1-Fc protein causes expansion of monocyte numbers in marrow, monocytosis, increased tissue macrophages and growth of the liver and spleen in WT mice {Gow et al., 2014;Keshvari et al., 2024}. “

The purpose of our experiment was to determine the effect of the *Fireko* on the response to CSF1 *in vivo* in light of the unresponsiveness of bone marrow cells *in vitro*. The conclusions are clear; the *Fireko* mice were resistant to CSF1-induced monocytosis and expansion of tissue macrophages. In effect, the *Fireko* mice provide additional evidence that the effect of CSF1-Fc is indeed dependent on activation of CSF1R.

5. Significance should be tested for this statement, especially if the data is shown in one plot "CSF1-Fc treatment caused an increase in total yield of lavage cells and an increase in
6. the % of large peritoneal macrophages within the lavage in WT mice."

We thank the reviewer for pointing this out. We have modified the results text to more accurately reflect the conclusions in Figure 3.

7. Previous point 10 has not been fully addressed: show gene locus, type of mutation,

© 2026. Published by The Company of Biologists under the terms of the Creative Commons Attribution License (https://creativecommons.org/licenses/by/4.0/).

genotyping protocols and validation of new AP1 mouse model, especially if this is the first paper that is using this new mouse line.

The complete protocol for the generation and genotyping of this mutation was provided in Materials and Methods. We have added additional details of the precise location of the mutation to the Methods and the nature of the mutation to the results text.

8. Previous point 13: not acceptable. Perform co-staining with at least 1 alveolar macrophage marker to show that the GFP signals overlaps 100% with this marker.

This demand is not justified in terms of understanding the effect of the *Fireko*. The reviewer is effectively suggesting that some alveolar macrophages, defined by SiglecF expression, might lack *Csf1r*-EGFP. The ubiquitous expression of *Csf1r*-EGFP by alveolar macrophages (AM) is not a point of contention.

Aside from the referenced paper by Tan and Krasnow, which used *Csf1r*-EGFP as a marker to study lung macrophage development, the original paper on the *Csf1r*-EGFP transgene showed uniform EGFP expression in macrophages obtained by bronchoalveolar lavage (PMID:1239359). The expression by AM and specificity was also confirmed in a study of the role of macrophages in postnatal alveolisation (PMID: 23560845). We have added references to these observations to the text. The same *Csf1r* promoter driving an mApple transgene labels 100% of SiglecF+ AM and has been used in live imaging of AM and interstitial macrophages in mouse lung (PMID: 39395172)

As we noted in previous response, the text already referred to the original description of the *Fireko* mice which showed that adults were not deficient in SiglecF+ alveolar macrophages on flow cytometry.

The text states clearly that at 3 weeks of age Fireko mice were deficient in *Csf1r*-EGFP$^+$ cells in the lung. Morphology and localisation within the airways are reasonable criteria to distinguish EGFP$^+$ AM from interstitial macrophages. In Fig S10 we show that in *Fireko* mice receiving WT bone marrow at 3 weeks, there was selective population of the stellate interstitial macrophages by donor-derived cells (CSF1R-FRed+), whereas the round cells within the airways (AM) remained EGFP+ (recipient origin).

"The text now reads "Similarly, in the lung, consistent with the selective reduction of interstitial *Csf1r*-GFP$^+$ cells at weaning (**Figure 5C**), donor cells populated the stellate interstitial population whereas, consistent with their retention in adult *Fireko* mice {Rojo et al, 2019}, the putative alveolar macrophages identified by more rounded morphology and location within the airways remained EGFP$^+$ (**Figure S10**)."

It may seem simple to confirm the overlap between SiglecF and *Csf1r*-EGFP even if it serves only to validate what is already known. However, the whole point of using the EGFP reporter is to enable whole mount imaging. Interstitial macrophages in the lung periphery are difficult to visualize in sections and poorly recovered by tissue disaggregation. IHC/IF to detect SiglecF is not possible on fresh whole mounts. To address this point we would need to generate a cohort of juvenile WT and *Fireko* mice and process them specifically to preserve both EGFP activity and SiglecF immunoreactivity and lung architecture. We do not believe this is necessary to support the conclusions and it could not be justified on our animal ethics approval.

9. Previous point 16: the figure has not been removed, and is shown in S7E, where it should remain, or even go to the main figures as it shows a clear effect in KO male mice. This figure has been omitted in the text. It is questionable why the authors would want to remove such significant data?

The original Figure S7E was in fact removed. It has now been restored as Figure S7E. It shows that there is no significant effect of the *Fireko* on any serum electrolytes, proteins, liver or renal function markers at 10 or 30 weeks. The lack of effect of the *Fireko* on steady state serum creatinine or urea is then replicated in a larger group in Figure S8E. The male-specific increase in

© 2026. Published by The Company of Biologists under the terms of the Creative Commons Attribution License (https://creativecommons.org/licenses/by/4.0/).

serum creatinine and urea was seen only on the adenine diet. The original text stated that "Disease severity was similar between genotypes histologically but circulating creatinine and urea levels were significantly elevated in male *Fireko* mice compared to WT (**Figure S8E**)." This may have given the impression that there was a male-specific effect of the *Fireko* on steady state renal function. This is not the case. To clarify, the text now reads; "Disease severity was similar between genotypes histologically but circulating creatinine and urea levels in adenine-treated male *Fireko* mice were increased to a greater extent compared to WT (**Figure S8E**)". We have also added specific reference to the known difference in the size of the kidney between males and females and more explicit reference to the lack of tubular inclusions. This finding contrasts to the recent study from He *et al* (2024) which indicated that macrophage depletion led to dysfunction in tubular clearance of particulate material.

10. Previous point 18: in light with my previous point, I would like to see this experiment.

"18. It would be of great interest to test whether the adoptive transfer can rescue the kidney pathology after injury to determine whether ontogeny plays a role in cellular / tissue responses"

There is no differential histopathology between the WT and *Fireko* mice on the adenine diet aside. The only effect of the *Fireko* mutation is a small additional increase in serum creatinine and urea in the male mice in response to tubular injury. That being the case it is difficult to see how the response could be rescued by bone marrow cell transfer. The question of renal macrophage ontogeny is rather complex and beyond the current paper. However, the comment reminded us of an omission. We have added a reference to the work of Salei et al. (PMID: 34518373) who reported that depletion of resident renal macrophages increased pathology associated with cisplatin exposure.

Second decision letter

MS ID#: dev.205655R1

MS TITLE: Depletion and replacement of tissue resident macrophages in mice with germ-line deletion of a conserved enhancer in the Csf1r locus.

AUTHORS: Yajun Liu, Sebastien Jacquelin, Isis Taylor, Emma K. Green, Omkar L. Patkar, Sahar Keshvari, Ginell Ranpura, Conan J. O. O'Brien, Eline Jessen, Emma Maxwell, Rachel Allavena, Alexandre Gallerand, Stoyan Ivanov, Antony Adamson, Neil E. Humphreys, Kim M. Summers, Katharine M. Irvine and David A. Hume

Dear Dr Hume,

I have now received all the referees reports on the above manuscript, and have reached a decision. The referees' comments are appended below.

The overall evaluation is positive and we would like to publish a revised manuscript in Development, provided that the referees' comments can be satisfactorily addressed. As you will see the referee would like an adjustment to a sentence in the Abstract so that it better reflects the findings. Please attend to all of the reviewers' comments in your revised manuscript and detail them in your point-by-point response. If you do not agree with any of their criticisms or suggestions explain clearly why this is so. If it would be helpful, you are welcome to contact us to discuss your revision in greater detail. Please send us a point-by-point response indicating your plans for addressing the referees' comments, and we will look over this and provide further guidance.

Reviewer 2

© 2026. Published by The Company of Biologists under the terms of the Creative Commons Attribution License (https://creativecommons.org/licenses/by/4.0/).

I thank the authors for the careful revision of the manuscript.
I still disagree to some extend that the kidney phenotype seen after injury is not worth following up, but see also that this may be beyond the scope of the current work. I therefore propose to change the following sentence in the abstract: "In a model of renal injury macrophage recruitment and histopathology were similar in WT and Fireko mice.", as indeed there were sex-specific differences observed

---

Second revision

Author response to reviewers' comments

Dear Professor Briscoe

Thank you for your response to our manuscript; “Depletion and replacement of tissue resident macrophages in mice with germ-line deletion of a conserved enhancer in the Csf1r locus” MS ID#: dev.205655.

As suggested we have made a minor change to the abstract whilst staying within the 180 word limit.

Yours sincerely

Third decision letter

MS ID#: dev.205655R2

MS TITLE: Depletion and replacement of tissue resident macrophages in mice with germ-line deletion of a conserved enhancer in the Csf1r locus.

AUTHORS: Yajun Liu, Sebastien Jacquelin, Isis Taylor, Emma K. Green, Omkar L. Patkar, Sahar Keshvari, Ginell Ranpura, Conan J. O. O’Brien, Eline Jessen, Emma Maxwell, Rachel Allavena, Alexandre Gallerand, Stoyan Ivanov, Antony Adamson, Neil E. Humphreys, Kim M. Summers, Katharine M. Irvine and David A. Hume

Dear Dr Hume,

I am happy to tell you that your manuscript has been accepted for publication in Development, pending our standard publication integrity checks.

© 2026. Published by The Company of Biologists under the terms of the Creative Commons Attribution License (https://creativecommons.org/licenses/by/4.0/).
